# Publisher Correction: Soft microgel networks stabilize and extend nozzle-free water jets

**DOI:** 10.1038/s41467-026-78674-8

**Published:** 2026-11-04

**Authors:** Atieh Razavi, Mehrzad Roudini, Andreas Winkler, Benno Liebchen, Regine von Klitzing, Suvendu Mandal, Amin Rahimzadeh

**Affiliations:** 1 Soft Matter at Interfaces, Department of Physics, Technische Universität Darmstadt, Darmstadt, Germany; 2 IFW Dresden, SAWLab Saxony, Group Acoustic Microsystems, Dresden, Germany; 3 Technische Universität Darmstadt, Darmstadt, Germany

**Keywords:** Gels and hydrogels, Chemical physics, Polymers

Correction to: *Nature Communications* https://doi.org/10.1038/s41467-026-76854-0, published online 22 August 2026

In the version of this article initially published, in the text directly above Eq. (1), there was a typographical error where the “similar to” symbol was missing from the text now reading “Equating these two energy scales (*E*_kin_ ∼ *E*_surf_).” The change has been made in the HTML and PDF versions of the article.

