# Supplementary material for: Soft microgel networks stabilize and extend nozzle-free water jets
Source: Nat Commun. 2026 Aug 22;17:8819. doi: 10.1038/s41467-026-76854-0 (PMC13499812; doi:10.1038/s41467-026-76854-0)
Supplement: Supplementary file 1 [file 41467_2026_76854_MOESM1_ESM.pdf]

Supplementary Information: Soft microgel networks stabilize and extend nozzle-free water jets

Atieh Razavi,[1] Mehrzad Roudini,[2] Andreas Winkler,[3] Benno Liebchen,[4]
Regine von Klitzing,[1] Suvendu Mandal,[4,*] and Amin Rahimzadeh[1,†]

[1]*Soft Matter at Interfaces, Department of Physics, Technische Universität Darmstadt, Hochschulstraße 8, 64289 Darmstadt, Germany*
[2]*IFW Dresden, SAWLab Saxony, Group Acoustic Microsystems, Helmholtzstr. 20, 01069 Dresden, Germany*
[3]*IFW Dresden, SAWLab Saxony, Acoustic Microsystems, Helmholtzstr. 20, 01069 Dresden, Germany*
[4]*Technische Universität Darmstadt, Karolinenplatz 5, 64289 Darmstadt, Germany*

Contents

I. Microgel characterizations 1

II. Overview of the jetting phenomenon. 4

III. Separation of Timescales: Interfacial Deformation and Surfactant Adsorption-Desorption Kinetics 5

IV. Interfacial shear rheology and interfacial dilatational elasticity 6

V. Flow profile during stretching 7

VI. Interfacial strain and jet velocity 8

VII. Mapping to experimental time scales 10

VIII. Simulated Surface Tension Dynamics of MG1 at both Expansion and Compression 10

IX. Polymeric reference system 10

X. Fabrication of the SAW devices 12

Supplementary References 13

I. Microgel characterizations

The terms "soft" and "stiff" microgels are defined quantitatively based on their elastic modulus, which is the most widely used and practically relevant measure of microgel stiffness [1–3]. To substantiate this classification, we have added new atomic force microscopy (AFM) indentation measurements of individual PNIPAM microgels in water deposited on a silicon substrate [see Fig. S1]. During indentation experiments, both the MG sample and the cantilever are immersed in water [see the inset in Fig. S1] at a controlled temperature of 20.0°C. After equilibration for at least 30 minutes, force–distance curves are obtained by indenting the cantilever tips into the adsorbed MG and the elastic modulus is extracted. The elastic modulus profiles extracted across the microgels clearly show that increasing cross-linker concentration leads to a systematic increase in stiffness.

The surface tension of microgel dispersions with varying cross-linker content was measured using a drop-shape analyzer. A pendant droplet was formed, and the surface tension was recorded at different dispersion concentrations of 0.01, 0.1, and 1 wt%, corresponding to Fig. S2a, S2b and S2c, respectively. The results show that softer microgels (MG1) reduce the surface tension more rapidly than the stiffer microgels (MG5 and MG10). Furthermore, increasing the microgel concentration accelerates the reduction in surface tension.

* suvendu.mandal@pkm.tu-darmstadt.de
† amin.rahimzadeh@pkm.tu-darmstadt.de

Shear viscosity measurements [Fig. S3] of microgel dispersions at different concentrations exhibit Newtonian behavior, with values comparable to that of pure water, indicating that dynamic viscosity does not significantly influence jet stability.

The choice of a surface pressure of 5 mN/m is based on our simulation data, which suggests that, upon sufficient elongation, the surface tension of the air-water interface, including stiff microgels, reaches approximately 65 mN/m [see Fig. 6 in the main text]. Previous studies [4] on PNIPAM microgels at fluid interfaces have shown that at a surface pressure of approximately 5 mN/m, the polymer shells of neighboring microgels are already in contact. Core-core contact typically occurs only at higher surface pressures, above 25 mN/m. To further support this, we have included additional AFM images of microgel monolayers prepared at 25 mN/m in the Supplementary Information [see Fig. S4]. In this compressed regime, where both shell-shell and core-core interactions are present, it becomes even more evident that individual soft microgels occupy a significantly larger interfacial area, nearly twice that of stiffer microgels, despite the higher particle number density of the latter. Specifically, stiffer microgels (MG10) exhibit a smaller individual interfacial footprint compared to softer microgels (MG1), due to the greater deformability and lateral spreading of the softer microgels at the interface. Consequently, to maintain the same surface pressure (i.e., the same reduction in surface tension), a larger number of stiffer microgels must adsorb per unit area compared to softer ones. This key observation from the AFM images highlights that softer microgels spread more extensively at the interface and, therefore, require fewer particles to achieve the same reduction in surface tension.

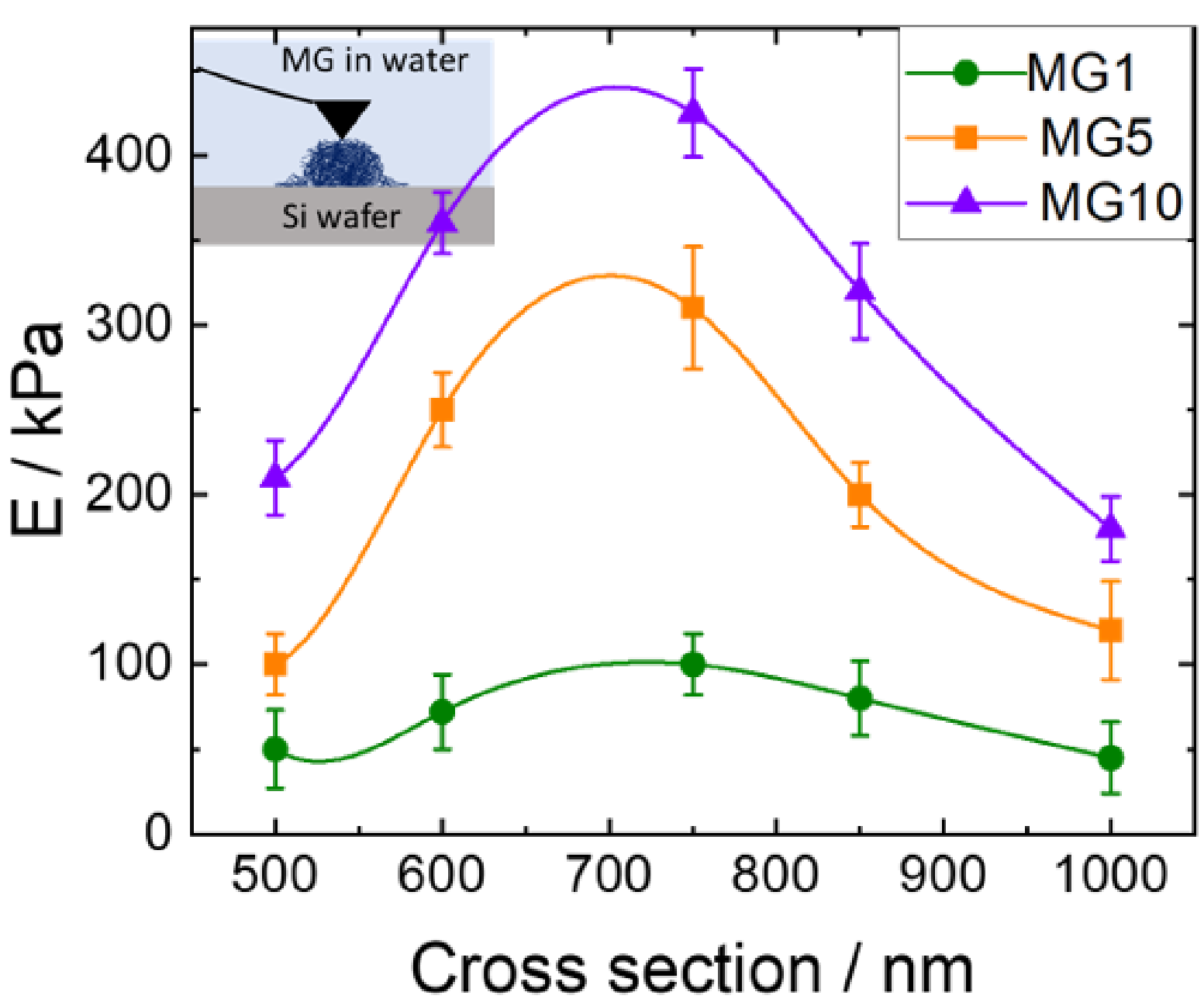

Figure S1. **Spatial variation of elastic modulus in PNIPAM microgels with different crosslinker densities.** Elastic modulus profiles across PNIPAM microgels with varying cross-linker densities, as measured by AFM indentation. The data show a systematic increase in stiffness with higher cross-linker concentration, with the modulus highest at the core and decreasing towards the periphery, consistent with the core-shell architecture of the microgels.

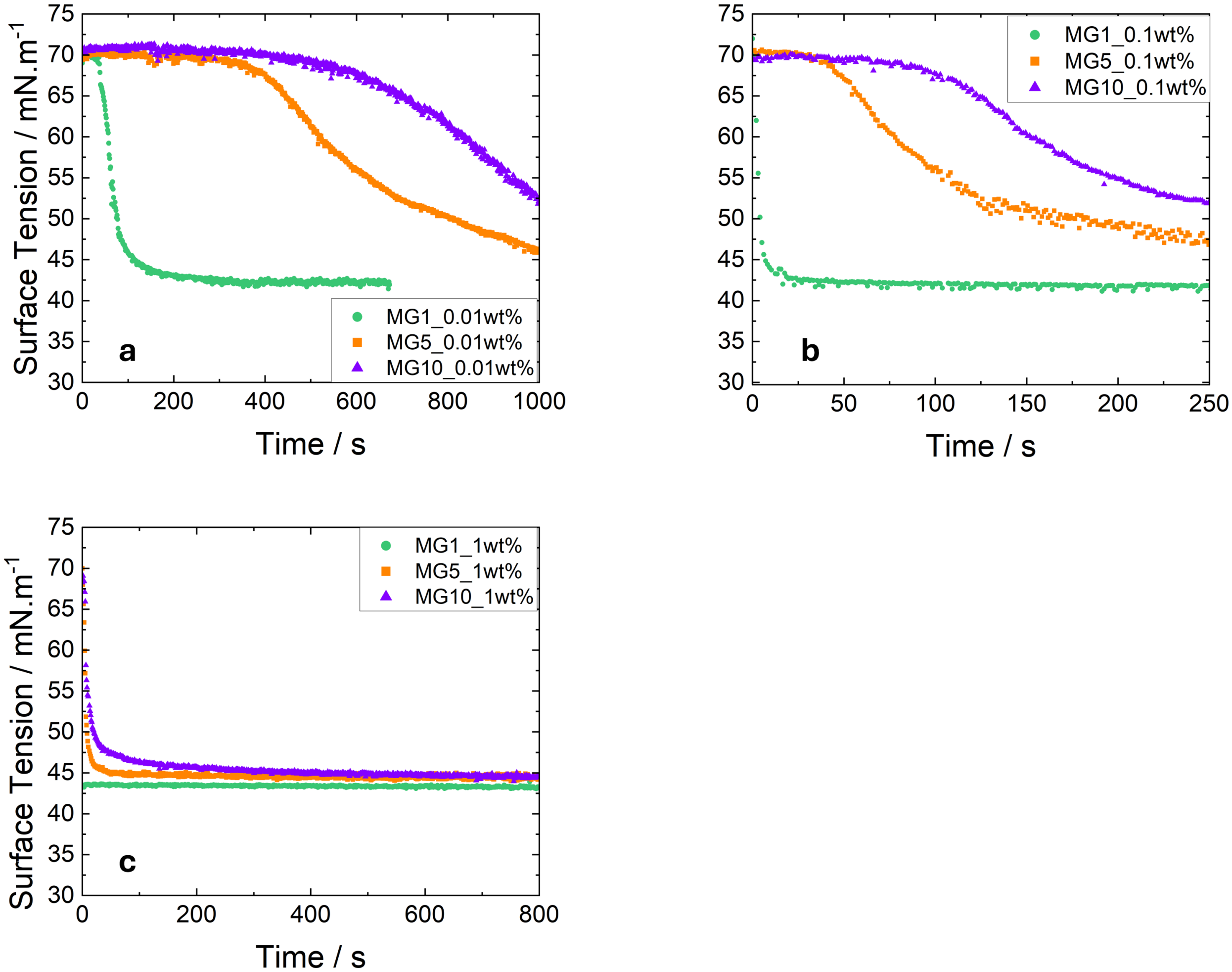

Figure S2. **Surface tension of microgel dispersions measured via pendant drop experiments.** Surface tension of PNIPAM dispersions at different cross-linker content (i.e., stiffness) and various concentrations of a) 0.01 wt%, b) 0.1 wt%, and c) 1 wt%. The time scale varies due to different kinetics at various concentrations. Jetting occurs at around 20 ms.

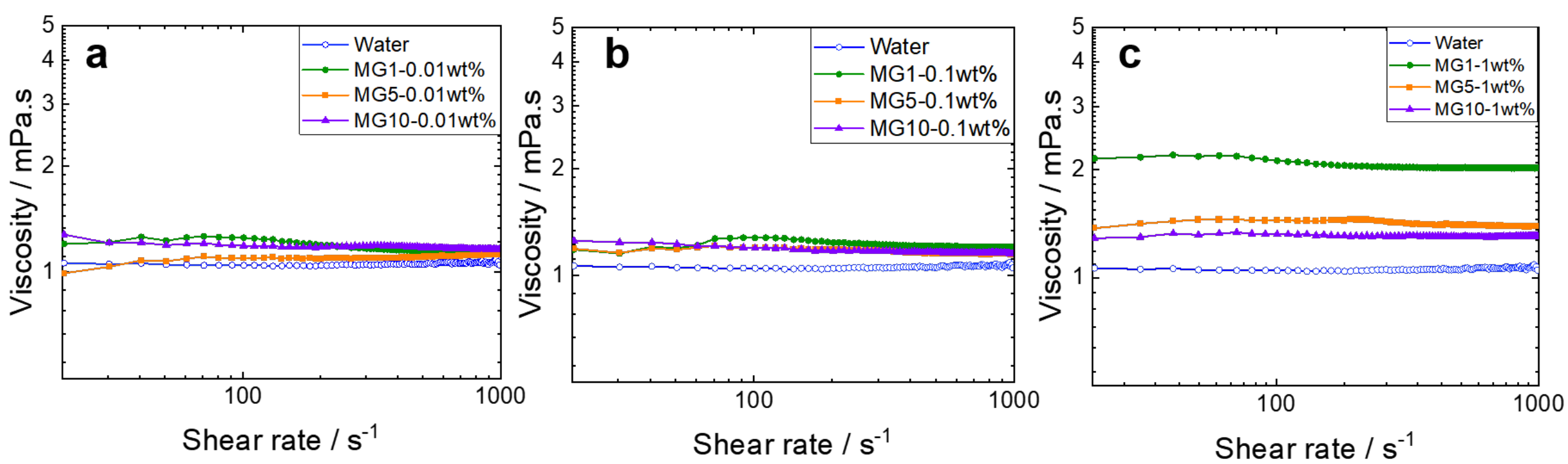

Figure S3. **Viscosity of microgel dispersions.** Shear viscosity of PNIPAM dispersions at different cross-linker content (i.e., stiffness) and various concentrations of a) 0.01 wt%, b) 0.1 wt%, and c) 1 wt% concentration.

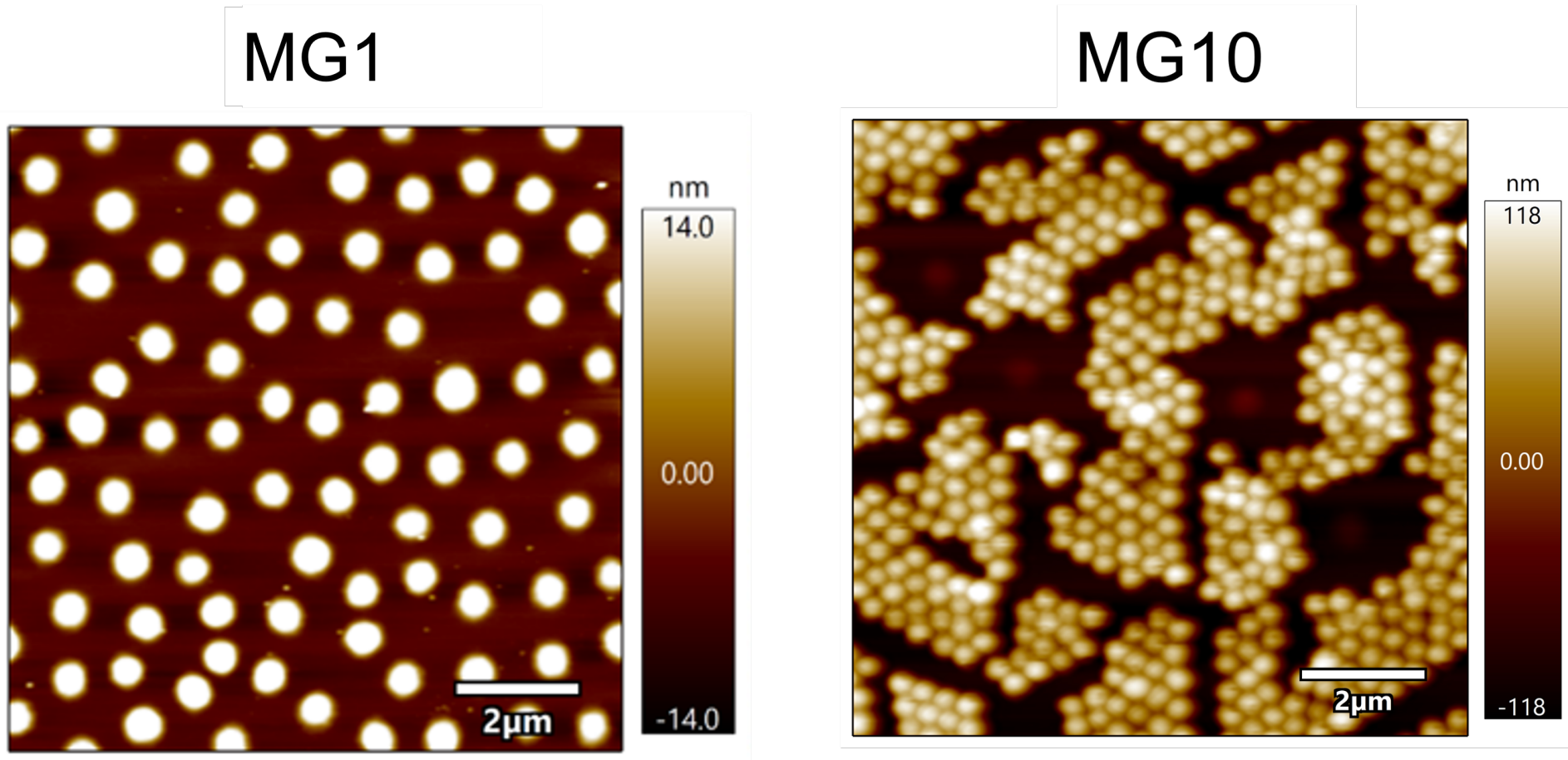

Figure S4. ***Ex-situ* AFM imaging of soft vs. stiff microgels at 25 mN/m surface pressure.** AFM images of microgels transferred ex situ onto a Si substrate using the Langmuir–Blodgett method at a surface pressure of approximately 25 mN/m, corresponding to (or close to) the onset of jetting. (a) Soft PNIPAM microgels and (b) stiff PNIPAM microgels. The soft microgels, with lower cross-linker density, display enhanced lateral deformability and a more spread, loosely organized arrangement. In contrast, the stiff microgels, possessing higher cross-linker density, appear less deformable and more densely packed under the same surface pressure.

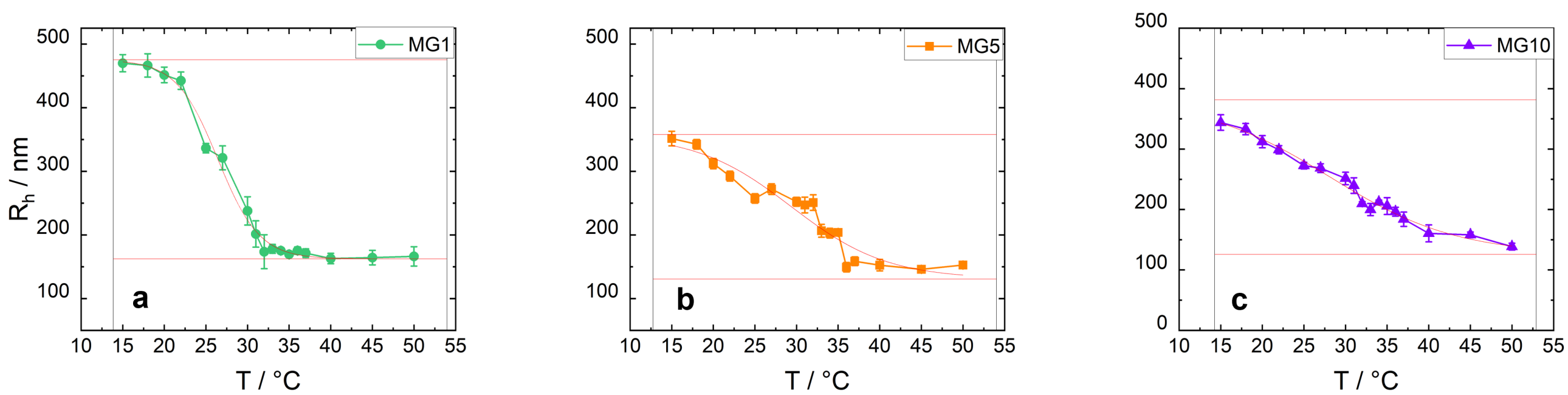

Figure S5. **Hydrodynamic radius at different temperatures** Hydrodynamic radius of PNIPAM microgels at different cross-linker contents of a) MG 1: 1 mol%, b) MG 5: 5 mol% and c) MG 10: 10 mol% cross-linker.

II. Overview of the jetting phenomenon.

Here, we present high-resolution time-resolved images of the jetting process for interfaces stabilized by soft microgels (MG1) [see Fig. S6]. The jet evolves dynamically over approximately 18 seconds, during which it maintains a stable cylindrical shape. Beyond this duration, Rayleigh–Plateau–like instabilities emerge, ultimately leading to jet breakup.

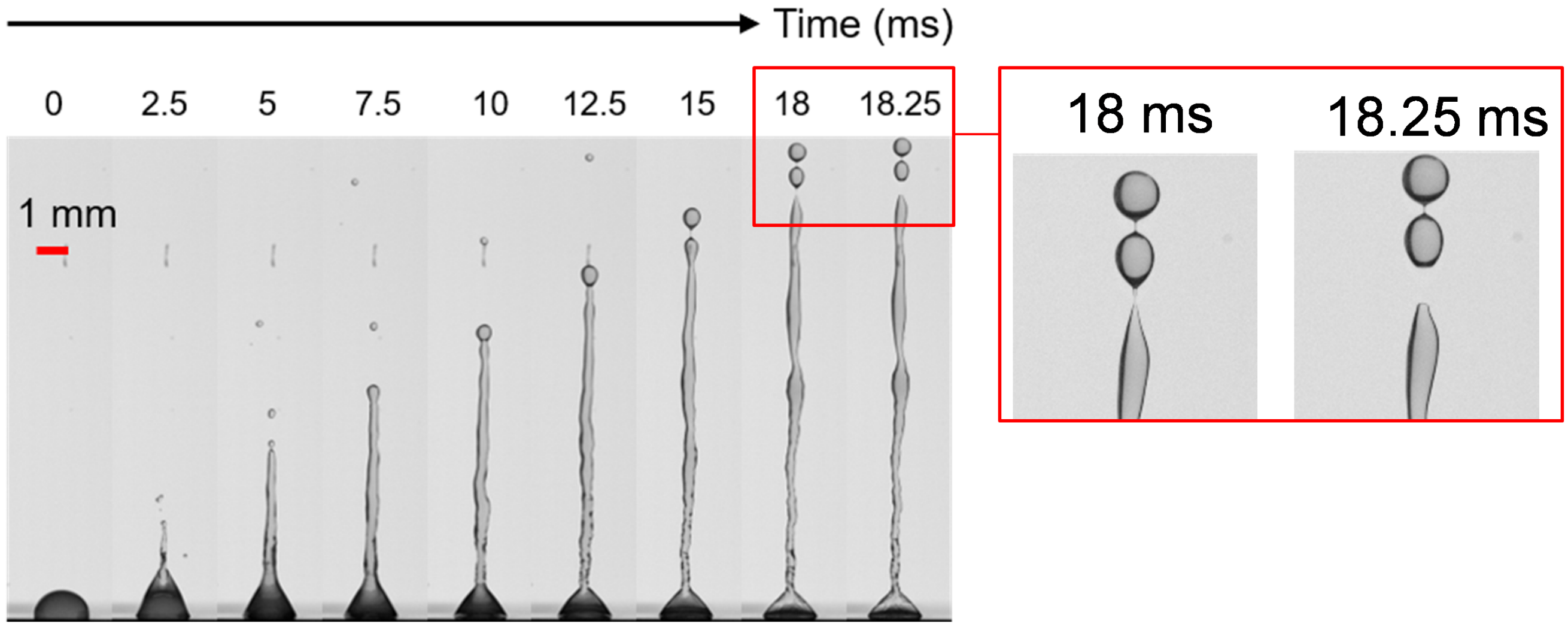

Figure S6. Jetting and break-up of a 1 wt% aqueous droplet containing microgels with 1 mol% cross-linker (MG1).

III. Separation of Timescales: Interfacial Deformation and Surfactant Adsorption-Desorption Kinetics

To clarify the separation of timescales between interfacial deformation rates ($1/\tau_{\mathrm{Jet}}$) and surfactant adsorption-desorption kinetics (τ_{ad}), we estimate the deformation rate using the jet diameter ($h \sim 0.2$ mm) and jetting velocity, yielding $\dot{\gamma} \approx u_0/h \approx 5000$ s^{-1} and a deformation timescale of $\tau_{\mathrm{Jet}} \sim 1/\dot{\gamma} \approx 0.2$ ms.

In contrast, cationic surfactants like C_{14}TAB and CTAB have adsorption-desorption times (τ_{ad}) on the order of seconds to minutes ($\tau_{\mathrm{ad}} \sim 1$s). Oscillating-drop experiments [5] show that while surfactant exchange rates increase with frequency, the re-equilibration timescale remains slower than the rapid interfacial deformation. This mismatch suggests that, under SAW-driven jetting occurring on millisecond timescales, surfactant monolayers cannot replenish fast enough between deformation events, leading to dynamic surfactant depletion instead of maintaining a uniformly covered, equilibrated layer. Experimental observations further support this: surfactant-stabilized jets, such as those with C14TAB, exhibit more irregular deformations, indicating that the interface cannot withstand rapid stretching [see Fig. S7].

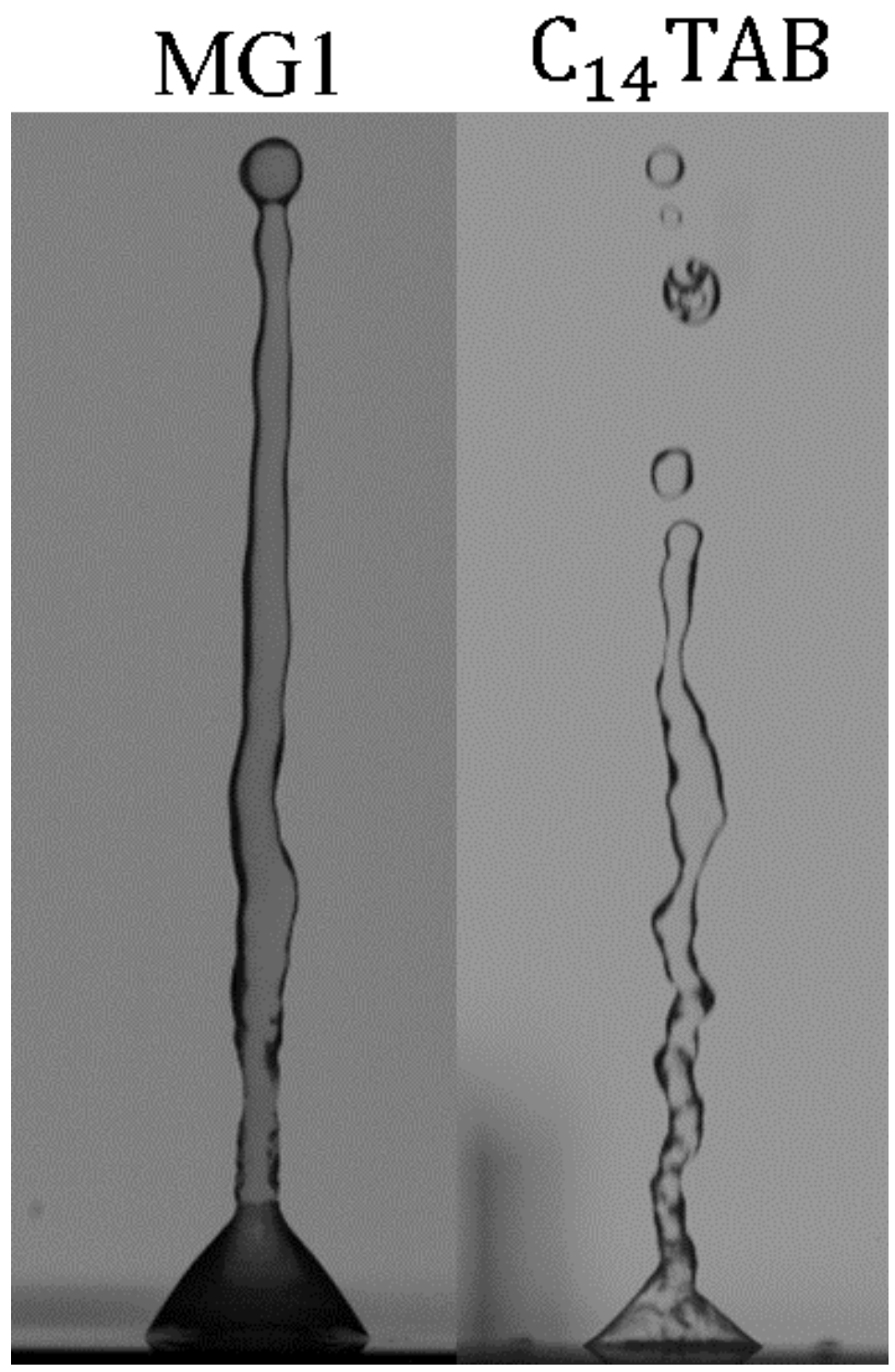

Figure S7. **Effect of surfactant C_{14}TAB on jet stabilization compared to microgels.** Representative snapshots illustrating the jet length for (left) 1wt% microgel dispersions with 1% crosslink density (MG1) and (right) for the cationic surfactant C_{14}TAB at concentrations well above the critical micelle concentration (CMC). The microgel-laden interface sustains a significantly longer and more stable jet. In contrast, the C_{14}TAB jet exhibits pronounced, irregular, and extreme interfacial deformations, indicating that the surfactant layer cannot efficiently smooth perturbations or withstand extensional stresses.

IV. Interfacial shear rheology and interfacial dilatational elasticity

We have performed new interfacial rheology measurements, including both interfacial shear rheology [see Fig. S8] and interfacial dilatational elasticity [see Fig. S9], for PNIPAM microgel–laden air–water interfaces. Both measurements show that interfaces stabilized by softer microgels exhibit higher dilatational elastic moduli and shear storage moduli than those formed by stiffer microgels. This behaviour indicates that softer microgels form interfacial layers with an enhanced ability to store elastic energy and to resist changes in interfacial area and shear deformations. Furthermore, interfacial shear rheology measurements show that networks formed by softer microgels sustain higher strains before yielding or loss of mechanical integrity. This indicates that softer microgels form more resilient, better-connected interfacial networks, capable of accommodating larger deformations before mechanical failure. However, we note that while these rheological measurements offer valuable insights into network robustness, they are not directly comparable to the jetting experiments. During SAW-driven jetting, the interface experiences extreme extensional strains, reaching over 200%, far beyond the deformation range typically encountered in standard interfacial rheology experiments. At such large strains, surface elasticity is no longer well-defined. Therefore, while interfacial rheology provides important information about the network's stability at smaller strains (below 10%), the jet stability in our system is ultimately governed by the interface's ability to maintain a low effective surface tension during rapid extension, rather than by its elastic moduli.

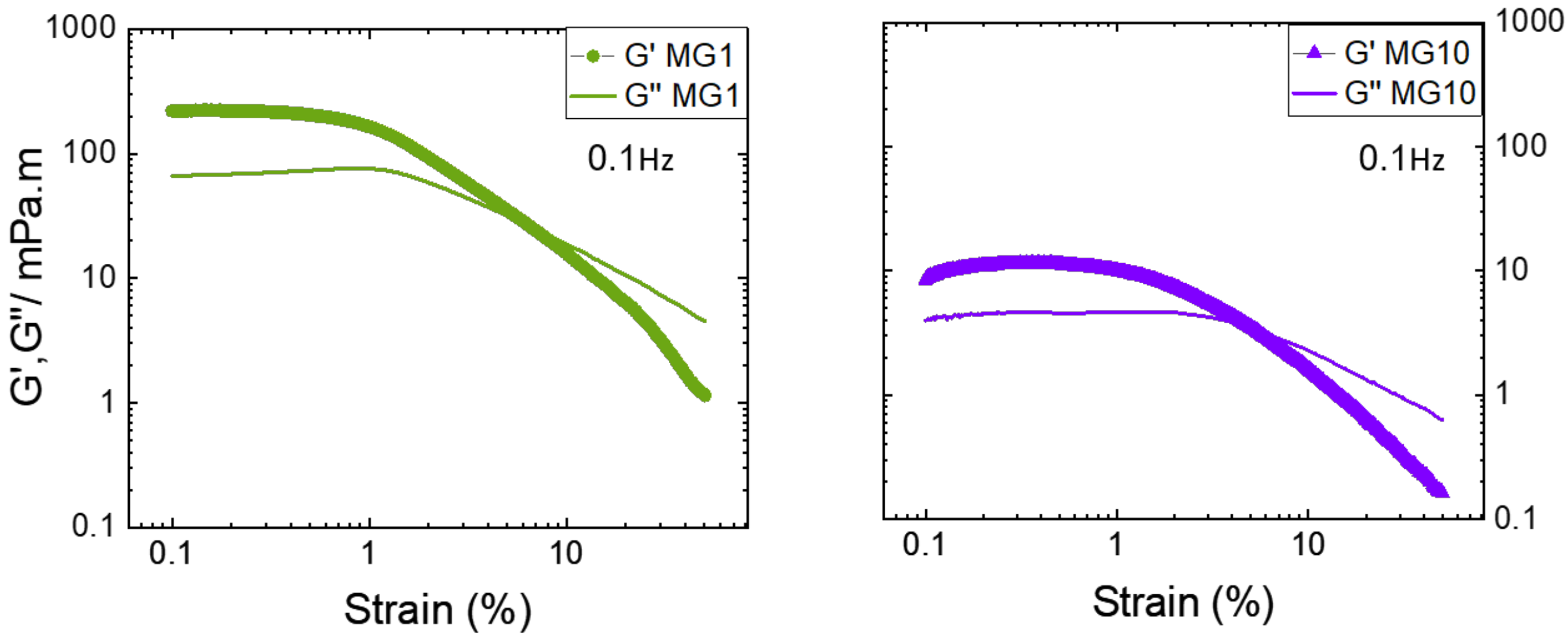

Figure S8. **Interfacial shear rheology of soft and stiff microgels.** The interfacial shear storage (G') and loss (G'') moduli were measured at a frequency of 0.1 Hz for soft and stiff microgels using 0.1 wt%. This concentration corresponds to a densely covered interface with an estimated surface pressure of $\sim$ 30 mN m^{-1}. Measurements were performed at this representative coverage, as the rheological setup is not coupled to a Langmuir trough for independent control of surface pressure.

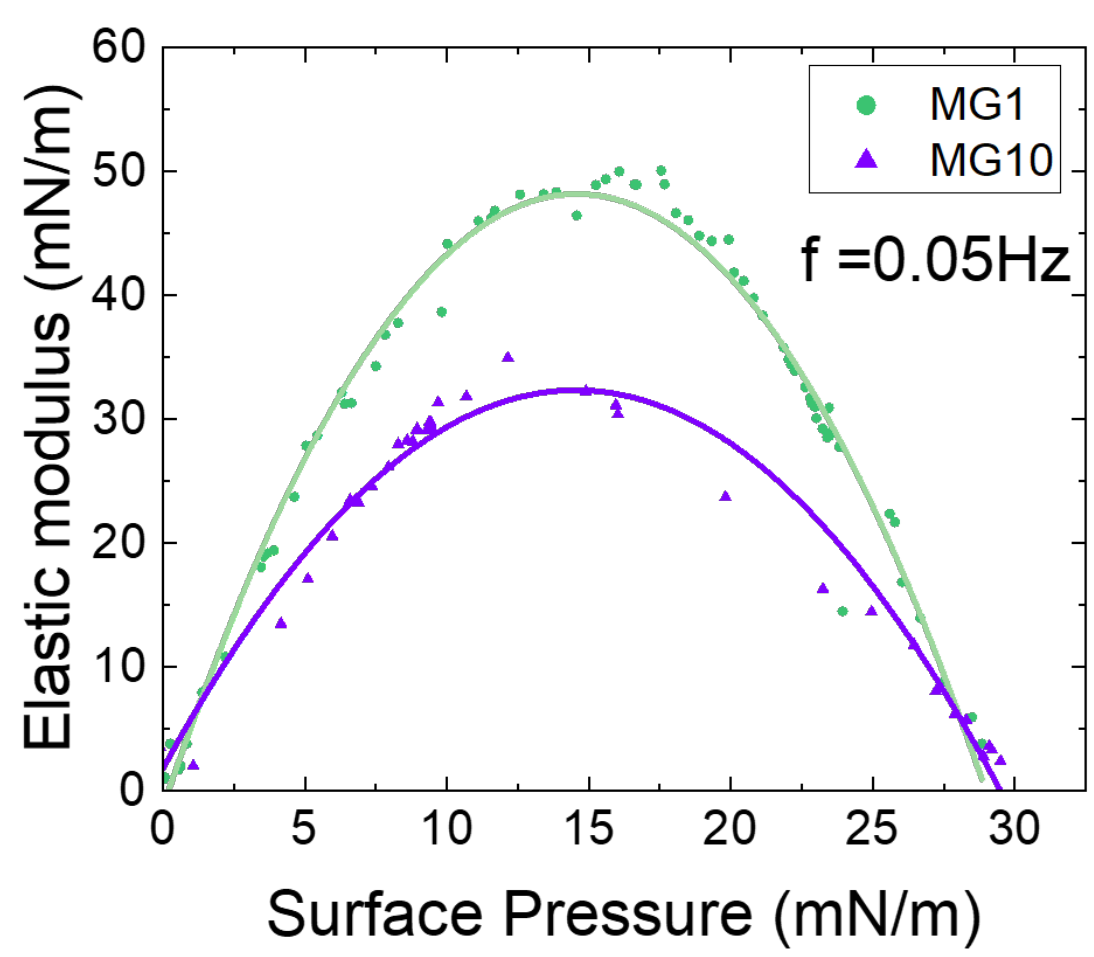

Figure S9. **Apparent dilatational elastic modulus as a function of surface pressure.** The elastic modulus, measured at the air–water interface using the oscillating drop method at a frequency of 0.05 Hz, is higher for the soft microgel (MG1) than the stiffer microgel (MG10).

V. Flow profile during stretching

To quantify the elongational stress peak, we calculated the elongational stress via pressure anisotropy, $\Delta p_{xz} = P_{zz} - P_{xx}$, following rheological models [6]. The elongational stress near the interface is directly related to surface tension via $\gamma = L_z \, \Delta p_{xz}$, where L_z is the sub-volume height [7]. We found that when the interface is exposed to air (as in MG10), the surface tension increases by approximately 10 mN/m compared to the microgel-covered interface

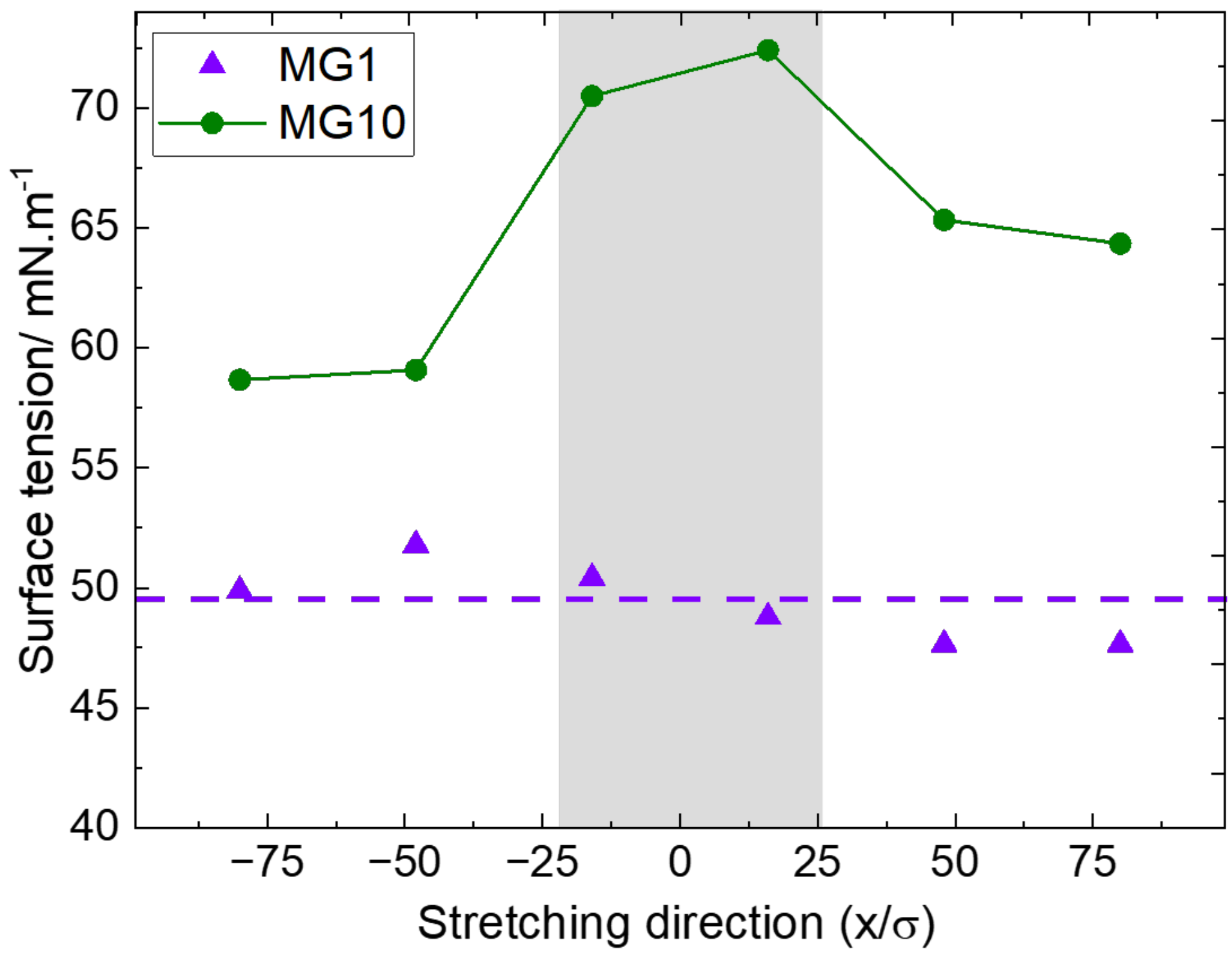

Figure S10. **Surface tension profiles of stiff (MG10) and soft (MG1) microgels during stretching of the interface.** Spatial variation of surface tension along the stretching axis. The grey region indicates the position of the bare air-water interface for stiff microgels. The surface tension at the center reaches the pure air-water interface value ($\gamma \approx 70$mN/m) for stiff microgels, while for soft microgels, the surface tension remains almost position-independent at $\gamma \approx 49$mN/m, which is significantly lower than the pure air-water surface tension.

[see Fig. S10]. In contrast, with soft microgels (MG1), surface tension remains stable over a wide range of stretching, maintaining a stable interface.

Although direct velocity measurements during jetting are experimentally inaccessible, we quantified instability by measuring the neck diameter evolution (Fig. S11), inspired by the previous experimental work [8]. For a pure water droplet, the neck diameter vanishes around 1.75ms, indicating rapid instability, whereas for the soft microgel-laden jet (MG1), breakup is delayed until approximately 4ms [see Fig. S11]. This significant delay highlights that soft microgel-covered interfaces are more resilient to elongational stresses.

VI. Interfacial strain and jet velocity

In the SAW-driven jetting process, the interfacial area of a sessile droplet undergoes significant expansion as the interface evolves from an initial area of approximately:

$$A_0 \sim \frac{1}{2}\pi R^2$$

to that of a cylindrical jet with surface area

$$A \sim \pi h L.$$

The corresponding areal strain can therefore be estimated as

$$\epsilon = \frac{\Delta A}{A_0} = \frac{A}{A_0} - 1 \approx \frac{2hL}{R^2} - 1.$$

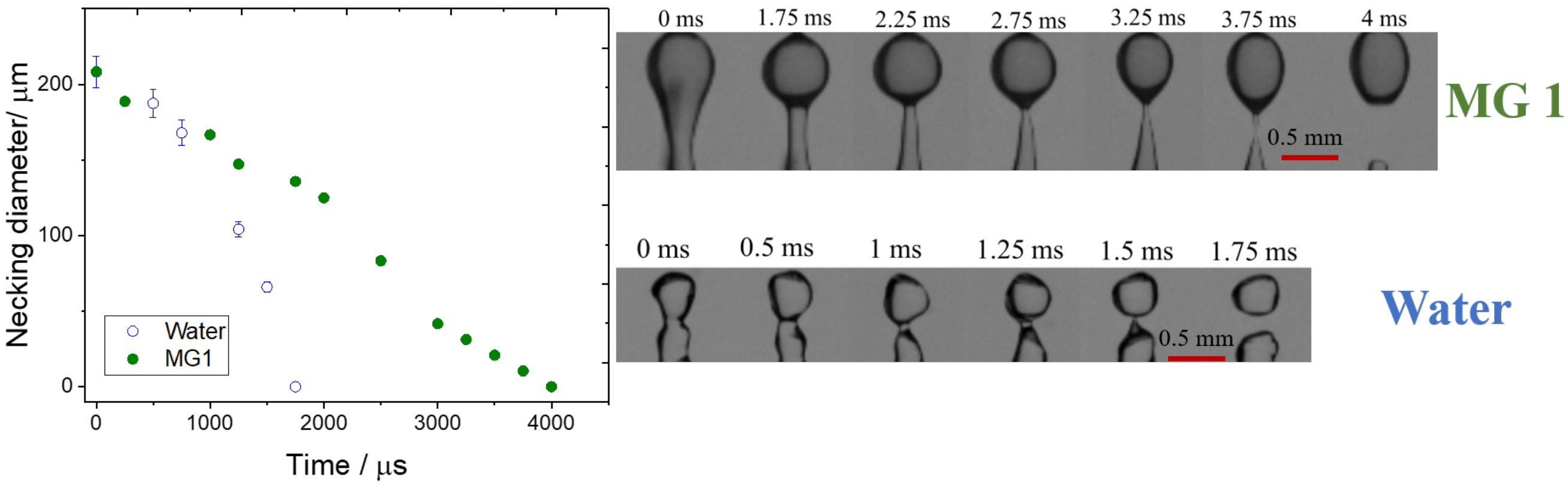

Figure S11. **Necking dynamics at the onset of jet breakup for pure water and soft microgels (MG1).** (Left) Time evolution of the neck diameter measured after the jet reaches its maximum length, illustrating the development of the Rayleigh–Plateau instability. (Right) Corresponding snapshots of the necking process. The pure water jet breaks into droplets when the neck diameter collapses at approximately (≈ 1.75ms), whereas, for soft microgels (MG1), the neck diameter remains nonzero until approximately 4ms. This delayed necking reflects the enhanced resistance of the microgel-stabilized interface to capillary breakup, resulting in a longer jet prior to fragmentation.

Using typical experimental values for the jet diameter ($h \sim 0.2$ mm), droplet base radius ($R \sim 1$ mm), and jet length ($L/R \approx 8$), the interfacial strain reaches over 200%, placing the system well beyond the elastic regime. While microgel stiffness indeed plays an important role in determining interfacial rheological properties, surface elasticity typically persists only at strains below approximately 10%. At larger strains, the microgel network leaves the elastic regime and undergoes a yielding, such that surface elasticity can no longer be treated as a well-defined property. Under such extreme and rapid interfacial deformations, surface elasticity does not remain a controlling parameter during jet elongation. Additionally, a low Capillary number ($\mathrm{Ca} = \mu u_0/\gamma \approx 0.01$, $\mu \sim \mathcal{O}(10^{-3}\ Pa.s)$, $u_0 \sim \mathcal{O}(1\ m\,s^{-1})$ and $\gamma \sim \mathcal{O}(10^{-1}\ Nm^{-1})$) indicates that viscous effects are negligible compared to surface tension. Therefore, the surface tension γ is the relevant interfacial parameter in the scaling analysis.

We agree with the reviewer that the derived scaling relation is descriptive rather than fully predictive, as the jet velocity is taken as a given parameter. Predicting the jet velocity in SAW-driven jetting is itself a complex problem, as it depends on the coupling between acoustic streaming, droplet geometry, and nonlinear interfacial dynamics, and is therefore beyond the scope of the present work. Tan *et al.* [9] introduced an acoustic forcing term to the leading-order axisymmetric jet momentum balance equation and predicted that the characteristic jet velocity can be estimated as:

$$u_0 = \sqrt{2L_j\,(F_s^y - g)},$$

where F_s^y is the acceleration associated with acoustic streaming, g is the gravitational acceleration, and L_j is the jet length. The acoustic streaming force can be approximated as

$$F_s^{(y)} \approx \alpha_0 \beta\, \dot{\xi}_{x_3}^{\,2}\, \mathrm{Re}_A,$$

where α_0 is the acoustic attenuation coefficient, β is the coefficient of nonlinearity of the liquid, $\dot{\xi}_{x_3}$ is the surface displacement velocity of the transducer, and Re_A is the acoustic Reynolds number.

We note that this formulation is not strictly predictive either, as the jet velocity depends explicitly on the jet length L_j, which is itself an outcome of the jetting process. Nevertheless, the expression provides a useful order-of-magnitude estimate.

Using representative parameters reported in Ref. (14) for water droplets, we take a surface displacement amplitude of $\xi_0 \sim 10$ nm and a driving frequency of $f \sim 64$ MHz, which gives a surface displacement velocity of

$$\dot{\xi}_{x_3} = 2\pi f \xi_0 \sim 4\ \mathrm{m\,s^{-1}}.$$

Assuming a typical jet length of $L_j \sim 10$ mm and neglecting gravity compared to the acoustic streaming contribution, this leads to an estimated jet velocity on the order of

$$u_0 \sim 1\ \mathrm{m\,s^{-1}},$$

which is consistent with experimentally observed SAW-driven jet velocities reported in the literature.

Due to the complexity of the underlying acousto-fluidic physics, we use the experimentally observed initial jet velocity, set by tuning the input SAW power, typically around $u_0 \sim 1\ \mathrm{m\,s^{-1}}$, as the relevant input parameter for the scaling analysis.

VII. Mapping to experimental time scales

Inspired by pioneering simulation works [10, 11], we studied 10 nm microgels up to a time of $0.1\,\mu$s, which is the current computational limit. This simulated microgel size is approximately 50 times smaller than the experimental microgels (500 nm). This size difference leads to two key consequences: (1) Experimental microgels cover 50^2 larger surface area compared to the simulated microgels, and (2) larger microgels, with longer dangling polymers, exhibit stronger entanglement effects. According to de Gennes' Nobel-winning work [12], the disentanglement time scales with the cube of the polymer length. Therefore, for our 50 times larger experimental microgels, the disentanglement time would increase by a factor of 50^3. Consequently, if we could simulate microgels 50 times larger, the disentanglement time would be approximately:

$$0.1\,\mu\mathrm{s} \times 50^3 \approx 12\,\mathrm{ms}. \tag{1}$$

Notably, our experimental jetting experiments were conducted for 10 ms, suggesting that the timescales of our simulations align well with the experimental results, providing a consistent comparison between the two.

VIII. Simulated Surface Tension Dynamics of MG1 at both Expansion and Compression

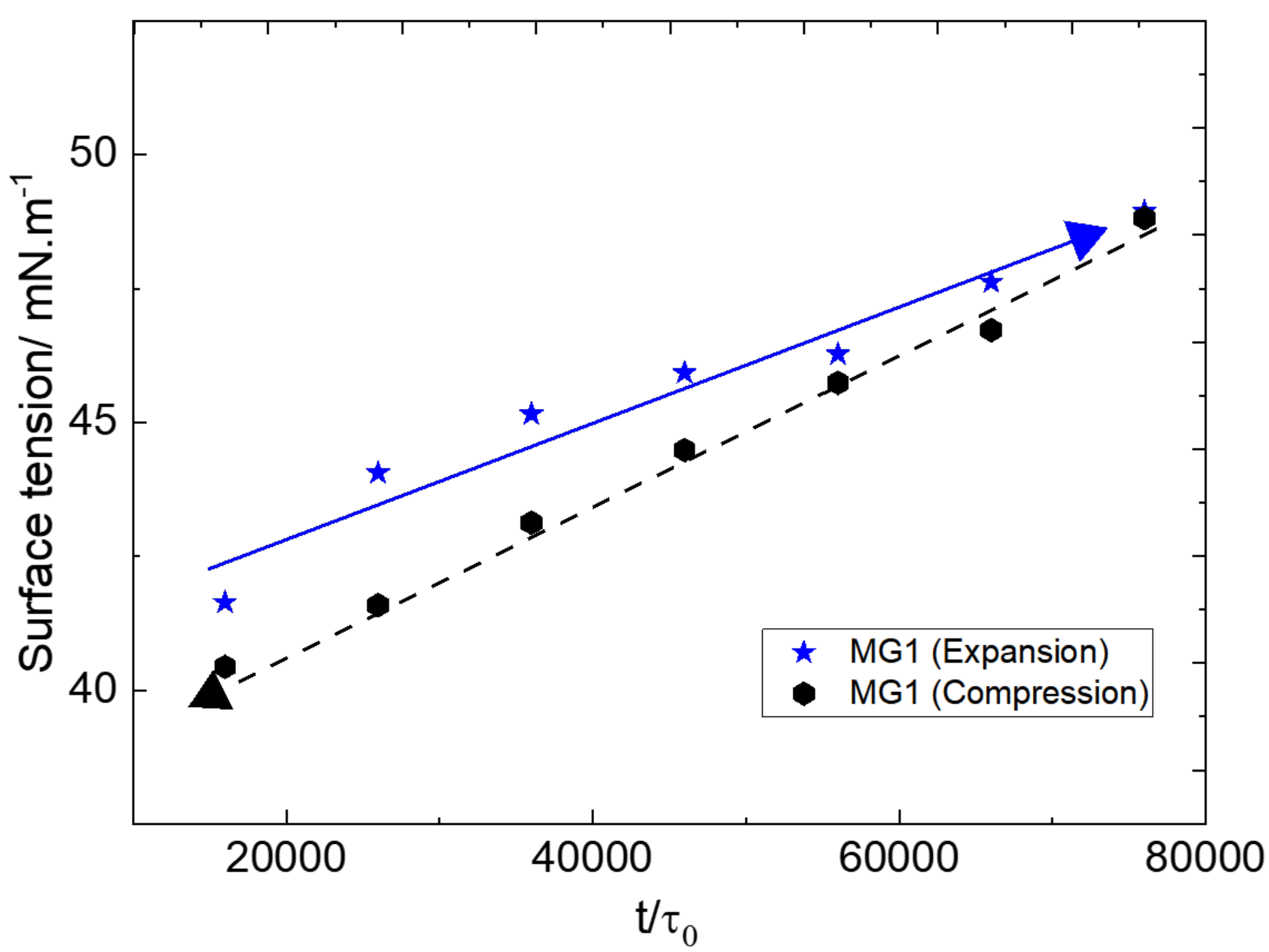

Figure S12. The time evolution of the dynamic surface tension for microgels with the lowest cross-linker density (MG1) shows a slight discrepancy between compression and expansion.

IX. Polymeric reference system

We performed experiments with an additional polymeric reference system to further clarify the role of polymer chemistry, interfacial activity, bulk polymer effects, and microgel architecture. PEG was selected as a polymeric

reference because, unlike PNIPAM, it exhibits only weak surface activity. The 35 kDa PEG is particularly relevant because its molecular weight is comparable to that of the LPNIPAM used in this study ($M_w \approx 40$ kDa).

As shown in Fig. S13(a), 35 KDa PEG solution at a concentration of 1 wt% exhibits equilibrium surface tensions of approximately 62 mN/m, whereas both LPNIPAM and MG1 reduce the surface tension to approximately 43 mN/m. Correspondingly, Fig. S13(b,c) shows that the PEG solution produces substantially shorter jets than either LPNIPAM or MG1. Despite having a molecular weight comparable to that of LPNIPAM, PEG-35 kDa behaves much more similarly to water than to the PNIPAM-based systems.

These observations do not isolate every contribution independently; however, they provide an important framework for distinguishing the role of PNIPAM-mediated interfacial activity from the mere presence of dissolved polymer chains. These mechanisms are proposed schematically in Fig. S14.

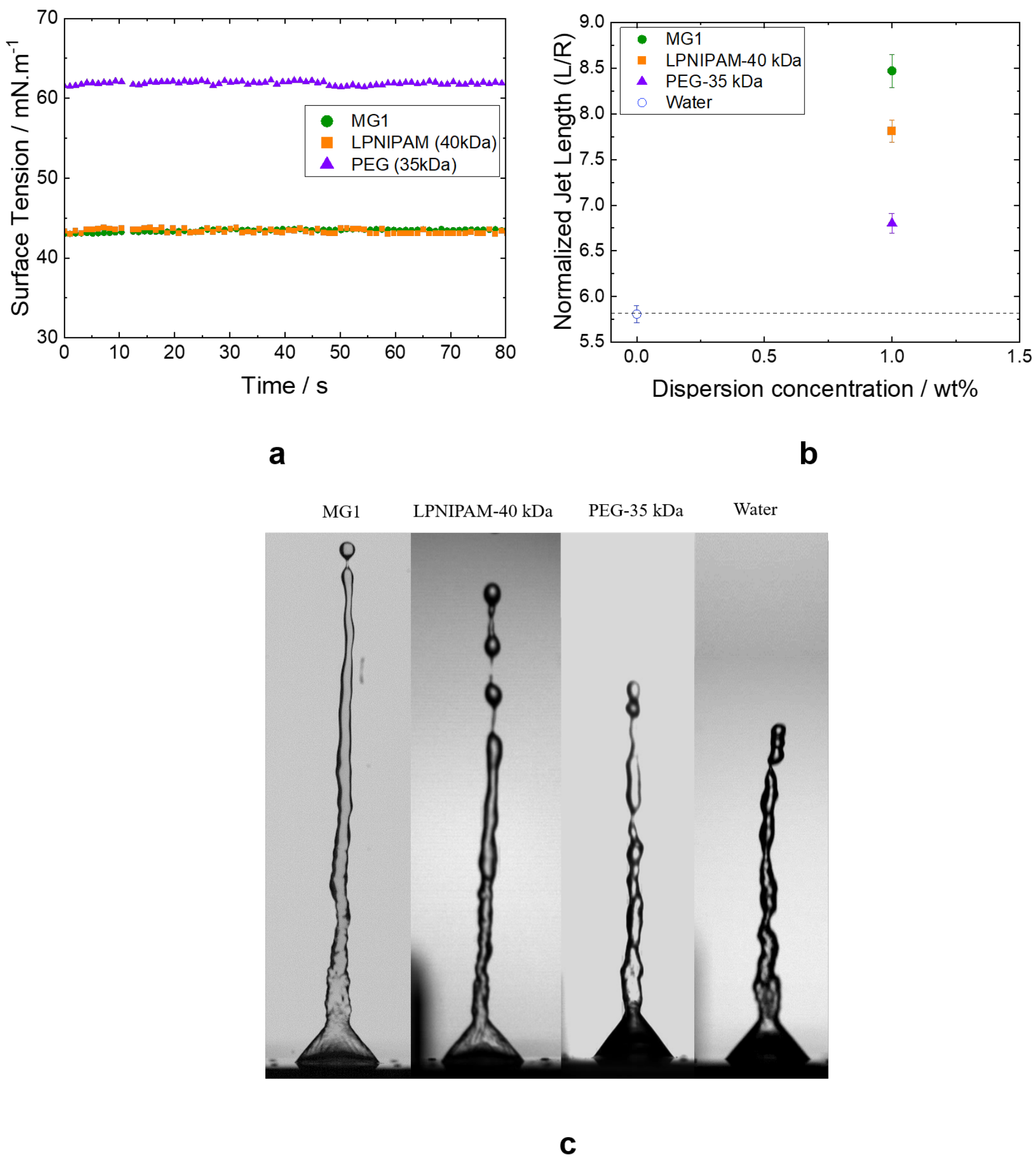

Figure S13. **Additional polymeric reference systems.** (a) Surface tension kinetics of softest microgel (MG1), Linear PNIPAM (M_w=40 kDa) as well as PEG (M_w=35 kDa) solutions at 1 wt% solution concentration. (b) The comparison of the normalized jet length across these systems. (c) The snapshot of the maximum jet length for MG1, linear PNIPAM and PEG solutions as well as water.

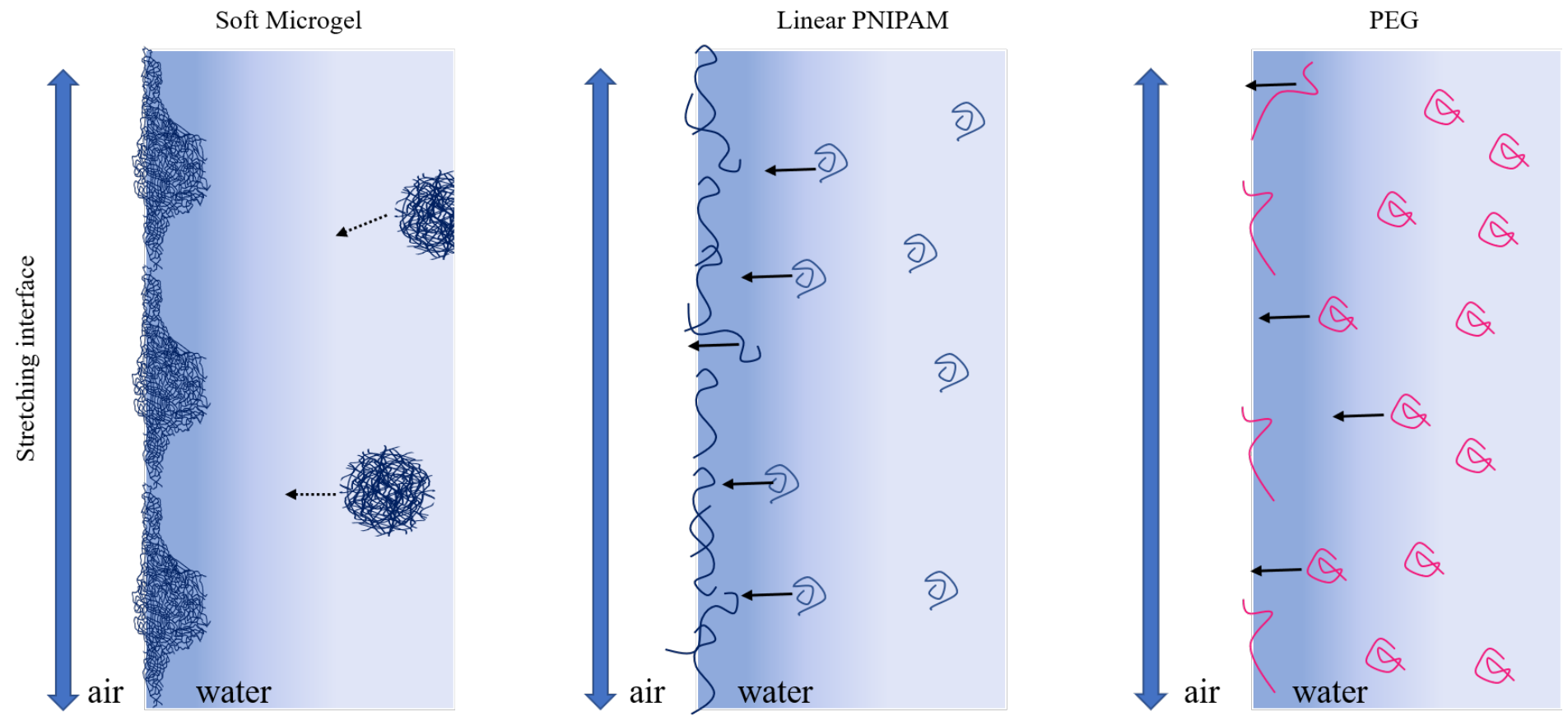

Figure S14. **Schematic illustration of the proposed stabilization mechanism.** The left panel shows a soft PNIPAM microgel adsorbed at the air–water interface. Owing to its deformability, the microgel spreads extensively at the interface, maintaining a low surface tension, while its large size results in relatively slow diffusion from the bulk (dotted arrows). The middle panel depicts linear PNIPAM chains, which diffuse rapidly from the bulk (solid arrows) and exhibit high surface activity, enabling efficient adsorption at the interface. In contrast, linear PEG chains (right panel) also diffuse rapidly but possess substantially lower surface activity, resulting in reduced adsorption and lower surface density than linear PNIPAM chains at the air–water interface. Microgel elements adapted from [13] and modified.

X. Fabrication of the SAW devices

Focused surface acoustic wave (SAW) devices were fabricated at the wafer level on single-side-polished 128° YX-cut lithium niobate ($LiNbO_3$) substrates, with SAW propagation along the X direction. Prior to photolithographic processing, the $LiNbO_3$ substrates were sequentially cleaned in acetone and isopropanol using an ultrasonic bath for 5 min each. The substrates were subsequently dried under a nitrogen stream and baked on a hot plate at 120 °C for 5 min. Residual organic contaminants were removed by O_2–plasma treatment for 5 min to improve photoresist adhesion.

For fabrication of the interdigital transducers (IDTs), the image-reversal photoresist AZ 5214E (MicroChemicals GmbH, Germany) was spin-coated at 4000 rpm for 35 s, resulting in a resist thickness of approximately 1.5 μm. The coated wafer was prebaked at 105 °C for 3 min and subsequently exposed using a maskless lithography system (MLA100, Heidelberg Instruments GmbH, Germany) at a wavelength of 365 nm with an exposure dose of 18 $\mathrm{mJ\,cm^{-2}}$. An image-reversal bake was then carried out at 120 °C for 2 min, followed by a flood exposure at 375 nm for 30 s. The resist was developed in AZ 726 MIF (MicroChemicals GmbH, Germany) for approximately 23 s and rinsed with deionized water.

The IDT electrodes consisted of a 5 nm titanium adhesion layer followed by a 295 nm aluminium layer. Both layers were deposited by electron-beam evaporation at deposition rates of approximately 1 $\mathrm{\AA\,s^{-1}}$ for Ti and 4 $\mathrm{\AA\,s^{-1}}$ for Al. The focused IDTs were designed with an acoustic wavelength of 60 μm, corresponding to an operating frequency of approximately 64.4 MHz. The curved electrode geometry focuses the generated Rayleigh-type surface acoustic wave into a localized focal region for liquid excitation.

Following metal deposition, lift-off was performed by immersing the wafer in a 1:1 mixture of isopropanol and acetone for approximately 90 min. During lift-off, the container was placed on a laboratory shaker operating at 150 rpm to facilitate removal of the photoresist and excess metal. The wafer was subsequently rinsed with acetone, followed by isopropanol, and dried under a nitrogen stream.

A 500 nm SiO_2 passivation layer was subsequently deposited over the wafer by reactive RF magnetron sputtering. The SiO_2 coating protects the metallic IDT structures against mechanical damage and corrosion while providing electrical isolation between the electrodes and liquids applied to the chip surface. To expose the electrical contact pads, a second photolithography step was carried out using the same resist processing conditions, followed by ion-beam etching to selectively remove the SiO_2 layer from the contact-pad regions. The SiO_2 etching was performed for approximately 10 min, after which the remaining photoresist was removed by lift-off.

Finally, the processed wafer was diced into individual SAW devices for subsequent experimental use.

Supplementary References

[1] S. Backes and R. Von Klitzing, Nanomechanics and nanorheology of microgels at interfaces, Polymers **10**, 978 (2018).

[2] A. Aufderhorst-Roberts, D. Baker, R. J. Foster, O. Cayre, J. Mattsson, and S. D. Connell, Nanoscale mechanics of microgel particles, Nanoscale **10**, 16050 (2018).

[3] G. Li, I. Varga, A. Kardos, I. Dobryden, and P. M. Claesson, Temperature-dependent nanomechanical properties of adsorbed poly-nipam microgel particles immersed in water, Langmuir **37**, 1902 (2021).

[4] M.-C. Tatry, E. Laurichesse, J. Vermant, V. Ravaine, and V. Schmitt, Interfacial rheology of model water–air microgels laden interfaces: Effect of cross-linking, J. Colloid Interface Sci. **629**, 288 (2023).

[5] J. Yang, K. Yu, T. Tsuji, R. Jha, and Y. Y. Zuo, Determining the surface dilational rheology of surfactant and protein films with a droplet waveform generator, J. Colloid Interface Sci. **537**, 547 (2019).

[6] T. C. O'Connor, N. J. Alvarez, and M. O. Robbins, Relating chain conformations to extensional stress in entangled polymer melts, Phys. Rev. Lett. **121**, 047801 (2018).

[7] L. Heitmeier and T. Voigtmann, Shear-rate dependent surface tension of glass-forming fluids, Phys. Rev. Lett. **136**, 068203 (2026).

[8] A. S. Utada, A. Fernandez-Nieves, H. A. Stone, and D. A. Weitz, Dripping to jetting transitions in coflowing liquid streams, Phys. Rev. Lett. **99**, 094502 (2007).

[9] M. K. Tan, J. R. Friend, and L. Y. Yeo, Interfacial jetting phenomena induced by focused surface vibrations, Phys. Rev. Lett. **103**, 024501 (2009).

[10] F. Camerin, M. Fernández-Rodríguez, L. Rovigatti, M.-N. Antonopoulou, N. Gnan, A. Ninarello, L. Isa, and E. Zaccarelli, Microgels adsorbed at liquid–liquid interfaces: A joint numerical and experimental study, ACS Nano **13**, 4548 (2019).

[11] S. Bochenek, F. Camerin, E. Zaccarelli, A. Maestro, M. M. Schmidt, W. Richtering, and A. Scotti, In-situ study of the impact of temperature and architecture on the interfacial structure of microgels, Nature Commun. **13**, 3744 (2022).

[12] P.-G. De Gennes, Reptation of a polymer chain in the presence of fixed obstacles, J. Chem. Phys. **55**, 572 (1971).

[13] S. Stock, L. Mirau, M. Rutsch, S. Wismath, M. Kupnik, R. von Klitzing, and A. Rahimzadeh, Ultrasound-induced adsorption of acousto-responsive microgels at water–oil interface, Adv. Sci. **11**, 2305395 (2024).
