# Supplementary material for: Soft microgel networks stabilize and extend nozzle-free water jets
Source: Nat Commun. 2026 Aug 22;17:8819. doi: 10.1038/s41467-026-76854-0 (PMC13499812; doi:10.1038/s41467-026-76854-0)
Supplement: Supplementary file 2 [file 41467_2026_76854_MOESM2_ESM.pdf]

Description of Additional Supplementary Files

File Name: Supplementary Movie 1
Description: Jetting with pure water. A 10-µl water droplet forms a jet under SAW excitation and breaks up after reaching its maximum length (10.82 mm).

File Name: Supplementary Movie 2
Description: Jetting with microgels. A 10-µl soft microgel (MG1) droplet forms a jet under SAW excitation and breaks up after reaching its maximum length (15.59 mm).

File Name: Supplementary Movie 3
Description: Computer simulation of soft microgels. During elongation, two soft microgels (MG2) remain interconnected, maintaining continuous coverage of the air–water interface.

File Name: Supplementary Movie 4
Description: Computer simulation of stiff microgels. Under identical conditions, two stiff microgels (MG10) lose contact and disentangle, exposing bare air–water interface.
