# Supplementary material for: Soft microgel networks stabilize and extend nozzle-free water jets
Source: Nat Commun. 2026 Aug 22;17:8819. doi: 10.1038/s41467-026-76854-0 (PMC13499812; doi:10.1038/s41467-026-76854-0)
Supplement: Supplementary file 3 [file 41467_2026_76854_MOESM7_ESM.pdf]

nature portfolio

Peer Review File

Soft microgel networks stabilize and extend nozzle-free water jets

Corresponding Author: Dr Amin Rahimzadeh

This file contains all reviewer reports in order by version, followed by all author rebuttals in order by version.

Version 0:

Reviewer comments:

Reviewer #1

(Remarks to the Author)
The authors investigate how the nanoscale softness of PNIPAM microgels stabilizes surface acoustic wave driven, nozzle-free water jets. They show experimentally that soft microgels (low cross-linker density) extend jet lengths by up to 44% compared to pure water. The mechanism is probed via dissipative particle dynamics simulations, which suggest that polymer entanglements in soft microgels maintain reduced effective surface tension under rapid interfacial strain. A scaling argument balancing injected kinetic energy with surface energy rationalizes the observed jet elongation. The work highlights a biocompatible, surfactant-free strategy for stabilizing high-strain jets, with implications for printing and drug delivery. While the study is interesting, major revisions are needed to improve clarity and rigor. Key issues include insufficient justification for experimental parameters, a lack of data points, and unclear characterization of rheological properties. Additional experiments, such as testing more surfactants at varying concentrations, are necessary, along with a deeper discussion of interfacial rheology and jet stability. Please see the following comments for the authors to improve the manuscript.

Comment 1: The authors state that a cross-link concentration of 1% corresponds to soft microgels and 10% to stiff microgels. Could the authors clarify which specific physical property is being used to define these classifications of "soft" and "stiff" microgels? That will be very meaningful for practical applications.
Comment 2: Could the authors clarify the rationale for selecting a lateral pressure of 5 mN/m for the interfacial organization experiments using Langmuir–Blodgett decomposition? What makes this pressure particularly relevant or appropriate?
Comment 3: Figure 2 suggests that conventional surfactants are less effective than microgels. To strengthen this conclusion, could the authors kindly consider testing additional surfactants and a broader range of concentrations? In particular including the surfactant concentration above the CMC?
Comment 4: Could the authors provide evidence to support the claim that extreme deformation rates driven by surface acoustic waves cause conventional surfactants to desorb? For instance, any time scale comparison that can be performed here, considering the desorption time scale of the surfactants? That will help to justify "…interfacial deformation occurs so rapidly that surfactants desorb before providing lasting stabilization"
Comment 5: In the scaling analysis, could the authors clarify whether surface elasticity and surface viscosity at the microgel-laden air-water interface play a role in jet stability? Since the microgels coat the jet surface and modify interfacial rheology, this aspect warrants further discussion. In addition, this scaling argument assumes velocity is given, so the relation is descriptive rather than predictive. I suggest the authors clarify the assumptions and limitations.
Comment 6: In the scaling analysis, it seems to the reviewer that the kinetic energy from the SAW to the droplet should contribute to an increase of surface energy, which should be estimated as the difference between the surface energy of the jet and the surface energy of the droplet. Can the author clarify this point?
Comment 7: For Figure S3 in the supplemental materials, is there a specific reason why the plot is not presented in a logarithmic scale? Additionally, are there other rheological properties of the PINPAM microgel water solution, such as viscoelasticity, that could be reported? Comparing these rheological data to previous studies would provide valuable contexts.
Comment 8: In Figure 3, how can it be determined that the microgels fully cover the jet surface? From the images, it appears that the cross-link concentration of 10% covers more of the jet surface area compared to the 1% concentration. Could the authors elaborate on this observation?
Comment 9: Could the authors clarify why a cross-link concentration of 1% can be used in experiments but not in simulations? Furthermore, how do the simulation results compare to the experimental findings?
Comment 10: In Figure 4, the authors show a schematic for the surfactant-laden jet ejection. Can the authors add a

schematic to contrast it with the microgel-laden jet ejection, which is the main topic in the current study?

(Remarks on code availability)
N/A

Reviewer #2

(Remarks to the Author)
The manuscript by Razavi et al. describes how soft PNIPAM microgels at liquid–air interfaces suppress capillary breakup in surface-acoustic-wave driven water jets. The authors combine experiments, simulations, and a scaling model to show that low-crosslink density microgels extend jet length by up to ~44 % compared to controls. They hypothesize that softer, less crosslinked microgels form entangled interfacial networks that remain cohesive under strong elongational flows, thereby resisting surface tension–driven breakup.

The study is exciting and convincingly demonstrates the potential of engineering liquid interfaces with microgel networks. At the same time, the mechanistic picture remains experimentally not fully complete: while the theoretical model supports the hypothesis, direct validation is limited, and other mechanistic explanations cannot yet be entirely ruled out given the complexity of the problem. Further experiments directly addressing these alternative possibilities will therefore be essential to firmly establish the proposed mechanism.

1. Flow profile within the jet: To understand how soft microgels extend jetting, it is necessary to resolve the flow profile inside the jet. Which forces act on the liquid interface, and at which positions do elongational stresses peak? Such measurements or simulations would be critical to link hydrodynamic stresses directly to interfacial stabilization.

2. Interfacial rheology in shear and dilation: Interfacial rheology is the natural experimental method to test the proposed mechanism. Shear and dilatational rheology could determine whether softer microgels increase not only the elasticity of the interface but also its yielding and failure thresholds. Establishing a correlation between rheological parameters (elastic modulus, dilatational modulus, relaxation time, yield stress) and jet extension would strongly support the authors' explanation.

3. Microgel monolayer rupture upon jet formation: The microgel monolayer likely ruptures at the onset of jet formation, as the rapid creation of new interface cannot be sustained solely by a pre-adsorbed layer. It remains an open question whether microgels dynamically adsorb to freshly created interfaces during jetting, and whether differences in adsorption kinetics between soft and stiff particles contribute to the observed stabilization. Addressing this point could help disentangle whether network elasticity or fast adsorption is the dominant factor.

4. Surfactants: On page 1, the authors write: "Yet in SAW-driven systems, interfacial deformation occurs so rapidly that surfactants desorb before providing lasting stabilization." This is a central statement, but no citation is provided. References or data supporting this claim should be included to justify the argument.

5. Range of tested systems: The authors tested three microgels with crosslinking densities from 1 mol-% to 10 mol-% and used CTAB as a reference. It would be useful to discuss whether the effect could be further enhanced by using ultra-low crosslinked microgels, which might form even more deformable and entangled networks. Similarly, a comparison to other surfactant systems, such as non-ionic surfactants or polymeric stabilizers (e.g. linear PNIPAM), could help distinguish whether the observed trends are unique to microgels or reflect a more general property of polymeric interfaces.

(Remarks on code availability)

Reviewer #3

(Remarks to the Author)
The manuscript by Razavi et al. presents acoustically driven nozzle-free jets by tuning the softness of microgels that can control interfacial resilience and extend jet length up to a considerable amount. The work is of potential interest for surfactant-free, biocompatible strategy in case of precision printing and needle-free drug delivery. It is well written with detailed experimental and simulation methods and results.

The manuscript is suitable for publication after the authors address my following comments.

1. It is mentioned, microgels are constructed from a self-assembled network using patchy particles. Equilibration of the 1% cross-linked network is slow. The authors should explain a bit more how the microgels are self-assembled and why equilibration of 1% network takes long time.

2. Considering Fig. 6, if the authors simulate long time, will MG2 reach to the results of MG5 and MG10, the bare air-water tension?

3. Comparing Fig. 6 and S2, Fig. 6 shows low tension to bare tension with time, whereas Fig S2 shows opposite. If

simulation is done in reverse directions from lateral stretched box to backwards (Fig. 5, from b to a and d to c), what will be the surface tension dynamics?

4. The time scales in Fig. 6 and S2 are different. Is it possible to map the time-scale for simulation and experimental results?

5. The authors should mention these works, Höfken et al. Rheologica Acta, 2025, doi.org/10.1007/s00397-025-01503-0 and Ruiz-Franco et al. Adv. Mater. Interfaces 2025, 12, 2500242, DOI: 10.1002/admi.202500242, regarding the change of viscoelastic properties of the interface through adsorption of microgels.

(Remarks on code availability)
There is no code available in that link.

Version 1:

Reviewer comments:

Reviewer #1

(Remarks to the Author)
I thank the authors taking time to address my concerns, and I appreciate the authors' additional clarification regarding the interfacial rheology and the proposed scaling law. The analysis of the elastic modulus and interfacial properties of the microgels is substantial and helpful. I recommend publication of the manuscript as it is.

(Remarks on code availability)

Reviewer #2

(Remarks to the Author)
The revised manuscript provides additional experiments and simulations that strengthen the overall presentation. Nevertheless, several aspects of the mechanistic interpretation remain insufficiently resolved. In particular, the current discussion largely attributes jet stabilization to interfacial effects associated with deformable microgel networks. However, alternative mechanisms related to bulk polymer physics are not fully considered and should be addressed more carefully.

The dimensional analysis assumes that viscous effects are negligible based on a small Capillary number calculated using the shear viscosity of water. However, this argument may not fully capture the rheology of polymer-containing systems. It is well established that dilute polymer solutions can exhibit substantially enhanced extensional viscosities while maintaining shear viscosities close to that of the solvent (Anna and McKinley, J. Rheol. 45, 115–138, 2001; Campo-Deaño and Clasen, J. Non-Newtonian Fluid Mech. 165, 1688–1699, 2010). Under strong elongational flows, such as those occurring during jet formation and thinning, even small concentrations of polymer can generate elastic stresses that delay capillary breakup. In this context, considering only the shear viscosity may not fully exclude potential viscoelastic contributions arising from extensional stresses in polymer-containing systems.

A related aspect concerns the experiments with linear PNIPAM. Linear polymer chains typically exhibit comparatively weak interfacial viscoelastic responses relative to particle-laden interfaces such as microgel monolayers. Nevertheless, the manuscript reports that linear PNIPAM produces jets with lengths reaching approximately 95% of those obtained with the softest microgels. This observation raises the question of whether the observed jet stabilization can be attributed solely to interfacial network mechanics, or whether contributions from polymeric material present in the bulk phase—such as dissolved polymer chains or microgels dispersed in the bulk—might also play a role.

The comparison to pure water and a molecular surfactant provides a useful reference point. However, given the similar jet lengths observed for linear PNIPAM and soft microgels, it may also be helpful to consider and discuss additional polymeric controls. In particular, a clearer description of the linear PNIPAM conditions (e.g., concentration) and their relation to the microgel experiments could help place these observations in context. Clarifying the relative contributions of interfacial microgel mechanics and possible bulk polymer effects would substantially strengthen the mechanistic interpretation of the results.

(Remarks on code availability)

Reviewer #3

(Remarks to the Author)
The Authors addressed all points I mentioned. Therefore I recommend the manuscript for publication.

(Remarks on code availability)

Version 2:

Reviewer comments:

Reviewer #2

(Remarks to the Author)
I thank the authors for their detailed response and for the additional discussion of possible bulk extensional polymer stresses. This has improved the manuscript. However, I remain concerned that the main mechanistic issue is not fully resolved.

Suitable polymeric reference systems are important for the interpretation, particularly given indications that non-crosslinked PNIPAM can produce jet lengths comparable to those obtained with the softest microgels. This directly bears on whether the effect is specific to soft microgel network mechanics or instead reflects a broader polymer-mediated interfacial stabilization mechanism.

The current comparison between microgels and molecular surfactants is valuable, but it does not provide a sufficiently complete reference framework to separate the roles of polymer chemistry, interfacial adsorption, possible bulk contributions, and microgel architecture. Additional polymeric reference systems would be needed to support the broader mechanistic conclusion.

I therefore remain unconvinced that the manuscript, in its present form, supports its central mechanistic conclusion that soft microgel networks are the key mechanism of jet stabilization.

(Remarks on code availability)

Version 3:

Reviewer comments:

Reviewer #2

(Remarks to the Author)
The authors have addressed all comments satisfactorily. The additional reference experiments clearly support the proposed interpretation and strengthen the manuscript. I therefore recommend acceptance.

(Remarks on code availability)

Open Access This Peer Review File is licensed under a Creative Commons Attribution 4.0 International License, which

permits use, sharing, adaptation, distribution and reproduction in any medium or format, as long as you give appropriate credit to the original author(s) and the source, provide a link to the Creative Commons license, and indicate if changes were made.
In cases where reviewers are anonymous, credit should be given to 'Anonymous Referee' and the source.
The images or other third party material in this Peer Review File are included in the article's Creative Commons license, unless indicated otherwise in a credit line to the material. If material is not included in the article's Creative Commons license and your intended use is not permitted by statutory regulation or exceeds the permitted use, you will need to obtain permission directly from the copyright holder.
To view a copy of this license, visit https://creativecommons.org/licenses/by/4.0/

1. Reply to Referee #1

> **Referee #1:** The authors investigate how the nanoscale softness of PNIPAM microgels stabilizes surface acoustic wave driven, nozzle-free water jets. They show experimentally that soft microgels (low cross-linker density) extend jet lengths by up to 44% compared to pure water. The mechanism is probed via dissipative particle dynamics simulations, which suggest that polymer entanglements in soft microgels maintain reduced effective surface tension under rapid interfacial strain. A scaling argument balancing injected kinetic energy with surface energy rationalizes the observed jet elongation. The work highlights a biocompatible, surfactant-free strategy for stabilizing high-strain jets, with implications for printing and drug delivery. While the study is interesting, major revisions are needed to improve clarity and rigor. Key issues include insufficient justification for experimental parameters, a lack of data points, and unclear characterization of rheological properties. Additional experiments, such as testing more surfactants at varying concentrations, are necessary, along with a deeper discussion of interfacial rheology and jet stability. Please see the following comments for the authors to improve the manuscript.
>
> Comment 1: The authors state that a cross-link concentration of 1% corresponds to soft microgels and 10% to stiff microgels. Could the authors clarify which specific physical property is being used to define these classifications of "soft" and "stiff" microgels? That will be very meaningful for practical applications.

We sincerely thank the Referee for their thoughtful and positive assessment of our manuscript. We greatly appreciate the suggestion to clarify the classification of "soft" and "stiff" microgels, which we have now addressed more explicitly and added a new Fig. R1.

In this work, the terms “soft” and “stiff” microgels are defined quantitatively based on their elastic modulus, which is the most widely used and practically relevant measure of microgel stiffness in the literature (Backes *et al.*, *Polymers* 10 (9), 2018, Aufderhorst-Roberts *et al.*, *Nanoscale* 10, 2018, Li *et al.*, *Langmuir* 37 (5), 2021). To substantiate this classification, we have added new atomic force microscopy (AFM) indentation measurements of individual PNIPAM microgels in water deposited on a silicon substrate [see Fig. R1 below]. During indentation experiments, both the MG sample and the cantilever are immersed in water [see the inset in Fig. R1] at a controlled temperature of 20°C. After equilibration for at least 30 minutes, force–distance curves are obtained by indenting the cantilever tips into the adsorbed MG and the elastic modulus is extracted. The elastic modulus profiles extracted across the microgels clearly show that increasing cross-linker concentration leads to a systematic increase in stiffness. In all cases, the modulus is highest in the core region and decreases toward the periphery, consistent with the well-known core–shell architecture of PNIPAM microgels.

Quantitatively, microgels with 10% cross-linker (MG10) exhibit the highest elastic modulus across the particle, followed by MG5, while microgels with 1% cross-linker (MG1) show the lowest modulus values throughout the particle. This establishes a clear and physically meaningful distinction between stiff (MG10), intermediate (MG5), and soft (MG1) microgels.

We added the following discussion in the main text:

‘*This classification as "soft" or "stiff" is based on their elastic modulus, which can be measured using atomic force microscopy (AFM) indentation of individual PNIPAM microgels in water, deposited on a silicon substrate. The elastic modulus profile reveals a systematic increase in stiffness with higher cross-linker concentrations. In all cases, the modulus is highest at the core of the microgel and gradually decreases toward the periphery, consistent with the well-established core-shell structure of PNIPAM microgels [see Fig. S1]. Quantitatively, microgels with 10% cross-linker (MG10) exhibit the highest elastic modulus across the particle, followed by MG5, while microgels with 1% cross-linker (MG1) show the lowest modulus values throughout the particle. This establishes a clear and physically meaningful distinction between stiff (MG10), intermediate (MG5), and soft (MG1) microgels.* ’

We added the following information in the SI.

‘*The terms “soft” and “stiff” microgels are defined quantitatively based on their elastic modulus, which is the most widely used and practically relevant measure of microgel stiffness [1–3]. To substantiate this classification, we have added new atomic force microscopy (AFM) indentation measurements of individual PNIPAM microgels in water deposited on a silicon substrate [see Fig. S1]. During indentation experiments, both the MG sample and the cantilever are immersed in water [see the inset in Fig. S1] at a controlled temperature of 20°C. After equilibration for at least 30 minutes, force–distance curves are obtained by indenting the cantilever tips into the adsorbed MG and the elastic modulus is extracted. The elastic modulus profiles extracted across the microgels clearly show that increasing cross-linker concentration leads to a systematic increase in stiffness.* ’

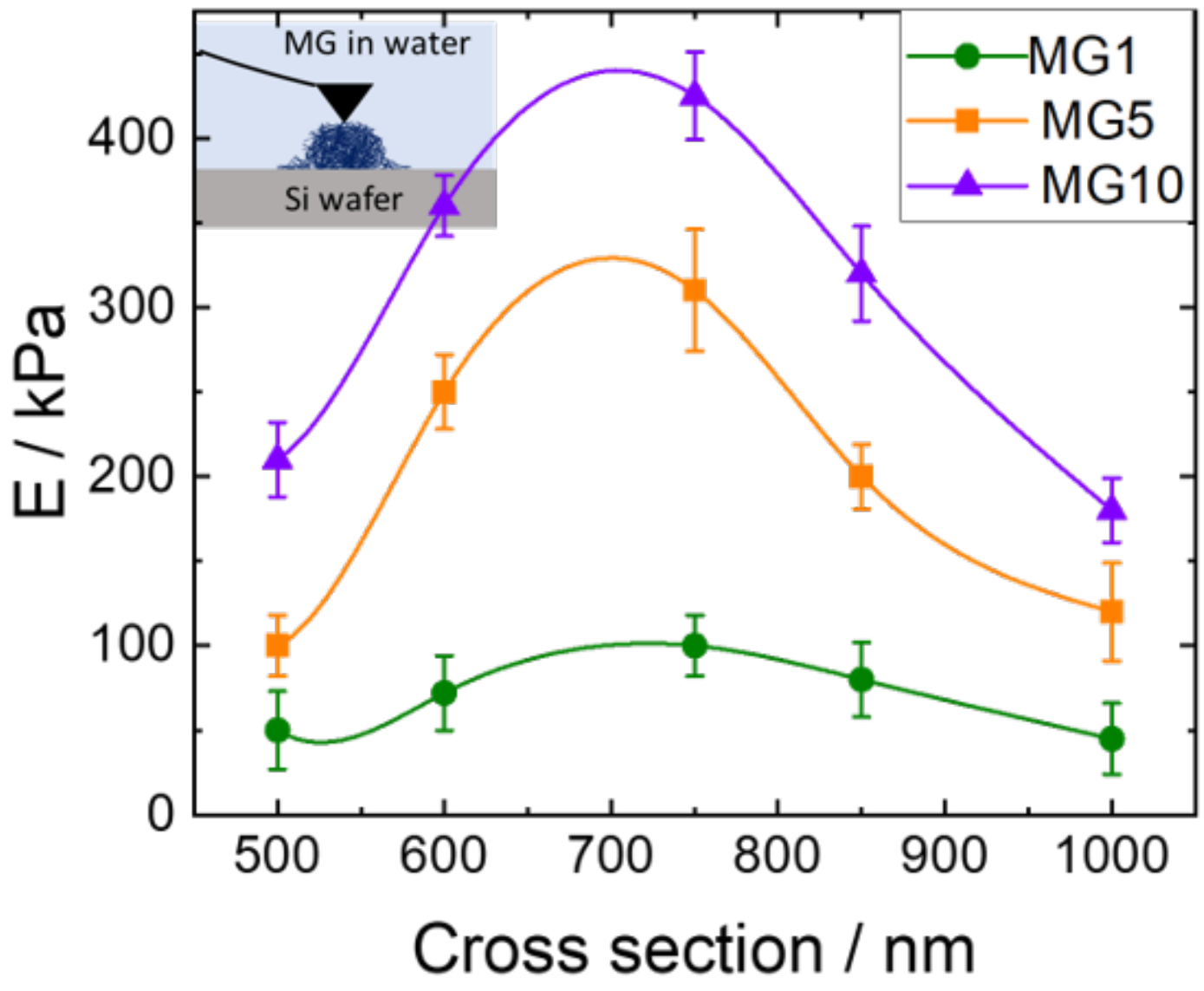

Figure R1: **Spatial variation of elastic modulus in PNIPAM microgels with different crosslinker densities.** Elastic modulus profiles across PNIPAM microgels with varying cross-linker densities, as measured by AFM indentation. The data show a systematic increase in stiffness with higher cross-linker concentration, with the modulus highest at the core and decreasing towards the periphery, consistent with the core-shell architecture of the microgels.

> **Referee #1:** Comments 2: Could the authors clarify the rationale for selecting a lateral pressure of 5 mN/m for the interfacial organization experiments using Langmuir–Blodgett decomposition? What makes this pressure particularly relevant or appropriate?

Reply: The choice of a surface pressure of 5 mN/m is motivated by our simulation data, which suggest that upon sufficient elongation, the surface tension of the air-water interface, including stiff microgels, reaches approximately 65 mN/m [see Fig. R2 below]. Hence, the surface pressure at this point is $\gamma_0 - \gamma \approx 5$ mN/m, where γ_0 represents the surface tension of the pure air-water interface (approximately 70 mN/m). This surface pressure of 5 mN/m corresponds to a physically relevant, non-equilibrium state typically encountered during the stretching phase of the jet, rather than at the onset of jet formation.

In contrast, at the onset of jet formation, the droplet interface is densely covered (i.e., less core-core distance) by microgels, resulting in a significantly higher surface pressure of approximately $25 - 30$ mN/m, as validated by both experiments and simulations [see Fig. R2]. In this initial regime, microgels are highly compressed, and both shell–shell and core–core contacts are expected. To further clarify this distinction, we have included Langmuir–Blodgett images acquired at approximately 25 mN/m in the revised SI [see Fig. R3], which depict the highly compressed interfacial state corresponding to conditions near jet initiation.

We have updated the main text accordingly.

'*.., selected to reproduce the interfacial conditions expected during jet elongation.* '

Here is the updated SI.

'*The choice of a surface pressure of* 5 *mN/m is based on our simulation data, which suggests that, upon sufficient elongation, the surface tension of the air-water interface, including stiff microgels, reaches approximately 65 mN/m [see Fig. 6 in the main text]. Previous studies [4] on PNIPAM microgels at fluid interfaces have shown that at a surface pressure of approximately* $5\ \mathrm{mN/m}$*, the polymer shells of neighboring microgels are already in contact. Core-core contact typically occurs only at higher surface pressures, above* $25\ \mathrm{mN/m}$*. To further support this, we have included additional AFM images of microgel monolayers prepared at* $25\ \mathrm{mN/m}$ *in the supplementary Information [see Fig. S4]. In this compressed regime, where both shell-shell and core-core interactions are present, it becomes even more evident that individual soft microgels occupy a significantly larger interfacial area, nearly*

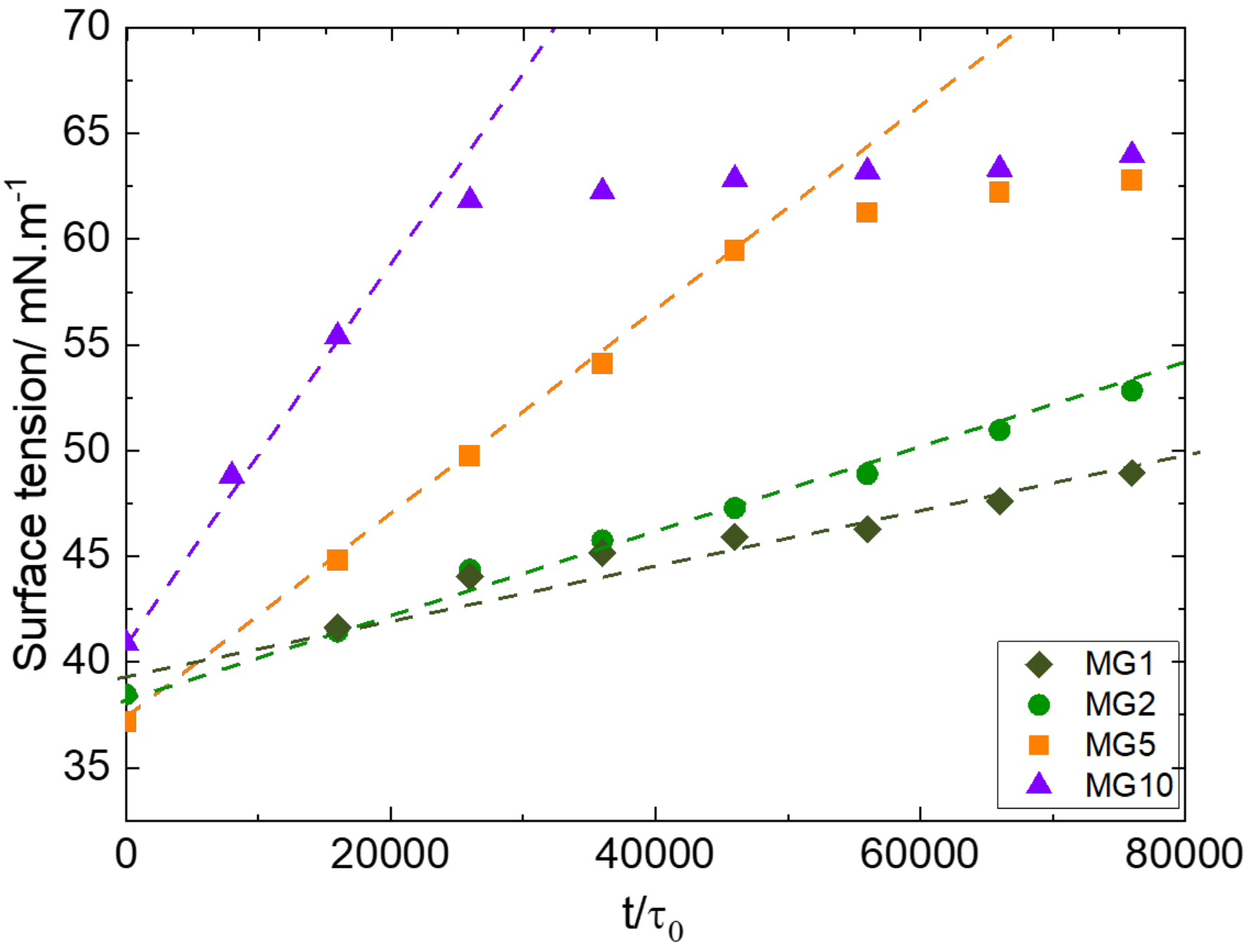

Figure R2: **Simulated surface tension dynamics.** Dynamic surface tension as a function of time for microgels with different cross-linker densities. The plot illustrates how varying the cross-linker concentration influences the rate at which the surface tension evolves, highlighting the distinct behaviors of soft (low cross-linker density) and stiff (high cross-linker density) microgels during interfacial stretching.

twice that of stiffer microgels, despite the higher particle number density of the latter. Specifically, stiffer microgels (MG10) exhibit a smaller individual interfacial footprint compared to softer microgels (MG1), due to the greater deformability and lateral spreading of the softer microgels at the interface. Consequently, to maintain the same surface pressure (i.e., the same reduction in surface tension), a larger number of stiffer microgels must adsorb per unit area compared to softer ones. This key observation from the AFM images highlights that softer microgels spread more extensively at the interface and, therefore, require fewer particles to achieve the same reduction in surface tension.].'

> **Referee #1:** Comments 3: Figure 2 suggests that conventional surfactants are less effective than microgels. To strengthen this conclusion, could the authors kindly consider testing additional surfactants and a broader range of concentrations? In particular including the surfactant concentration above the CMC?

Reply: Inspired by the Referee's suggestions, we have performed additional jetting experiments using the conventional surfactant C_{14}TAB at concentrations above its critical micelle concentration (CMC). Specifically, we tested surfactant concentrations at 1 CMC and 10 CMC [see Fig. R4]. Despite the significant increase in surfactant concentration, C_{14}TAB remains substantially less effective than PNIPAM microgels in extending the jet length. At 10 CMC, C_{14}TAB showed only a modest improvement ($\approx 11\%$) in jet length compared to pure water, while soft microgels (MG1) at 1 wt% dispersion concentration achieved a 44% increase compared to pure water [see Fig. R4 below]. This result suggests that the limited jet stabilization provided by molecular surfactants cannot be explained solely by insufficient surface coverage, but rather reflects intrinsic limitations of their interfacial response under high extensional strain.

While a systematic comparison across a broad range of surfactants with varying charge, molecular architecture, and adsorption kinetics would certainly be of interest, such an extensive study lies beyond the scope of the present work. Our objective here is not to exhaustively benchmark all surfactants, but rather to demonstrate that even at concentrations well above the CMC, a representative fast-adsorbing surfactant cannot reproduce the jet stabilization achieved by soft, biocompatible microgels.

Furthermore, C_{14}TAB is a cationic surfactant and is therefore expected to exhibit relatively fast adsorption kinetics compared to neutral or anionic surfactants [Paria *et al.*, *Ind. Eng. Chem. Res.* 44 (9), 2005]. The fact that even

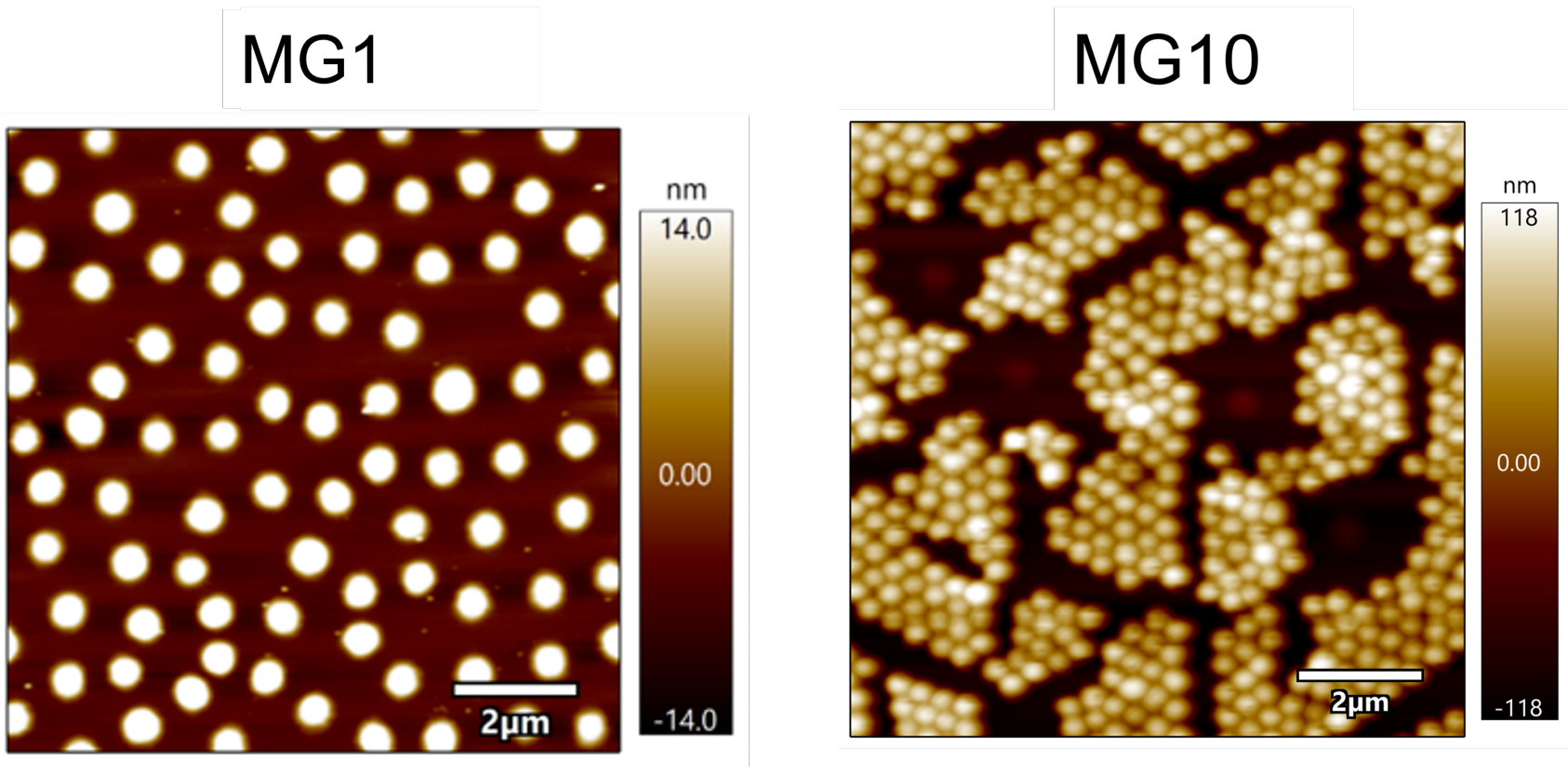

Figure R3: ***Ex-situ* AFM imaging of soft vs. stiff microgels at 25 mN/m surface pressure.** AFM images of microgels transferred ex situ onto a Si substrate using the Langmuir–Blodgett method at a surface pressure of approximately 25 mN/m, corresponding to (or close to) the onset of jetting. (a) Soft PNIPAM microgels and (b) stiff PNIPAM microgels. The soft microgels, with lower cross-linker density, display enhanced lateral deformability and a more spread, loosely organized arrangement. In contrast, the stiff microgels, possessing higher cross-linker density, appear less deformable and more densely packed under the same surface pressure.

such a fast-adsorbing surfactant fails to achieve stabilization comparable to that of microgels strongly supports our central conclusion: polymer-based interfacial stabilizers perform better under the rapid, high-strain interfacial dynamics relevant to jet formation. In particular, microgels can sustain non-equilibrium interfacial stresses through their deformable, polymeric architecture, which is not accessible to small-molecule surfactants.

We have clarified this point in the revised manuscript.

'*Even at higher concentrations (10 CMC), we observe a moderate improvement (≈ 11%) in jet length compared to pure water.* '

'*Furthermore, C_{14}TAB is a cationic surfactant and is therefore expected to exhibit relatively fast adsorption kinetics compared to neutral or anionic surfactants [47]. The fact that even such a fast-adsorbing surfactant fails to achieve stabilization comparable to that of microgels strongly supports our central conclusion: polymer-based interfacial stabilizers perform better under the rapid, high-strain interfacial dynamics relevant to jet formation [see Fig. S7].* '

> **Referee #1:** Comments 4: Could the authors provide evidence to support the claim that extreme deformation rates driven by surface acoustic waves cause conventional surfactants to desorb? For instance, any time scale comparison that can be performed here, considering the desorption time scale of the surfactants? That will help to justify "…interfacial deformation occurs so rapidly that surfactants desorb before providing lasting stabilization"

Reply: We thank the Referee for the insightful suggestion. Our claim that extreme deformation rates driven by surface acoustic waves (SAWs) cause conventional surfactants to desorb is supported by a clear separation of timescales between the interfacial deformation rate ($1/\tau_{\rm Jet}$) and surfactant adsorption–desorption kinetics ($\tau_{\rm ad}$). Estimating the deformation rate from the jet diameter ($h \sim 0.2\,$mm) and jetting velocity, we find $\dot{\gamma} \approx u_0/h \approx 5000\text{s}^{-1}$, which yields a deformation timescale $\tau_{\rm Jet} \sim 1/\dot{\gamma} \approx 0.2\,$ms.

In contrast, cationic surfactants like C_{14}TAB and CTAB have characteristic adsorption–desorption times ($\tau_{\rm ad}$) typically on the order of seconds to minutes ($\tau_{\rm ad} \sim 1$s). Oscillating-drop experiments (Yang *et al.*, *J. Colloid Interface Sci.* 537, 2019) show that while surfactant exchange rates increase with frequency, the timescale for surfactant re-equilibration remains longer than that of rapid interfacial deformation. This mismatch between the adsorption–desorption timescale and the deformation timescale suggests that under SAW-driven jetting conditions, which occur on millisecond timescales, the surfactant monolayer cannot replenish itself fast enough between successive deformation events. As a result, the interface most likely undergoes dynamic surfactant depletion, rather than maintaining a uniformly covered, equilibrated surfactant layer during jet elongation. This behavior is further supported by experimental observations of jet morphology: surfactant-stabilized jets, like those with C_{14}TAB, exhibit more irregular interfacial deformations, indicating that the interface cannot sustain a resistance under rapid

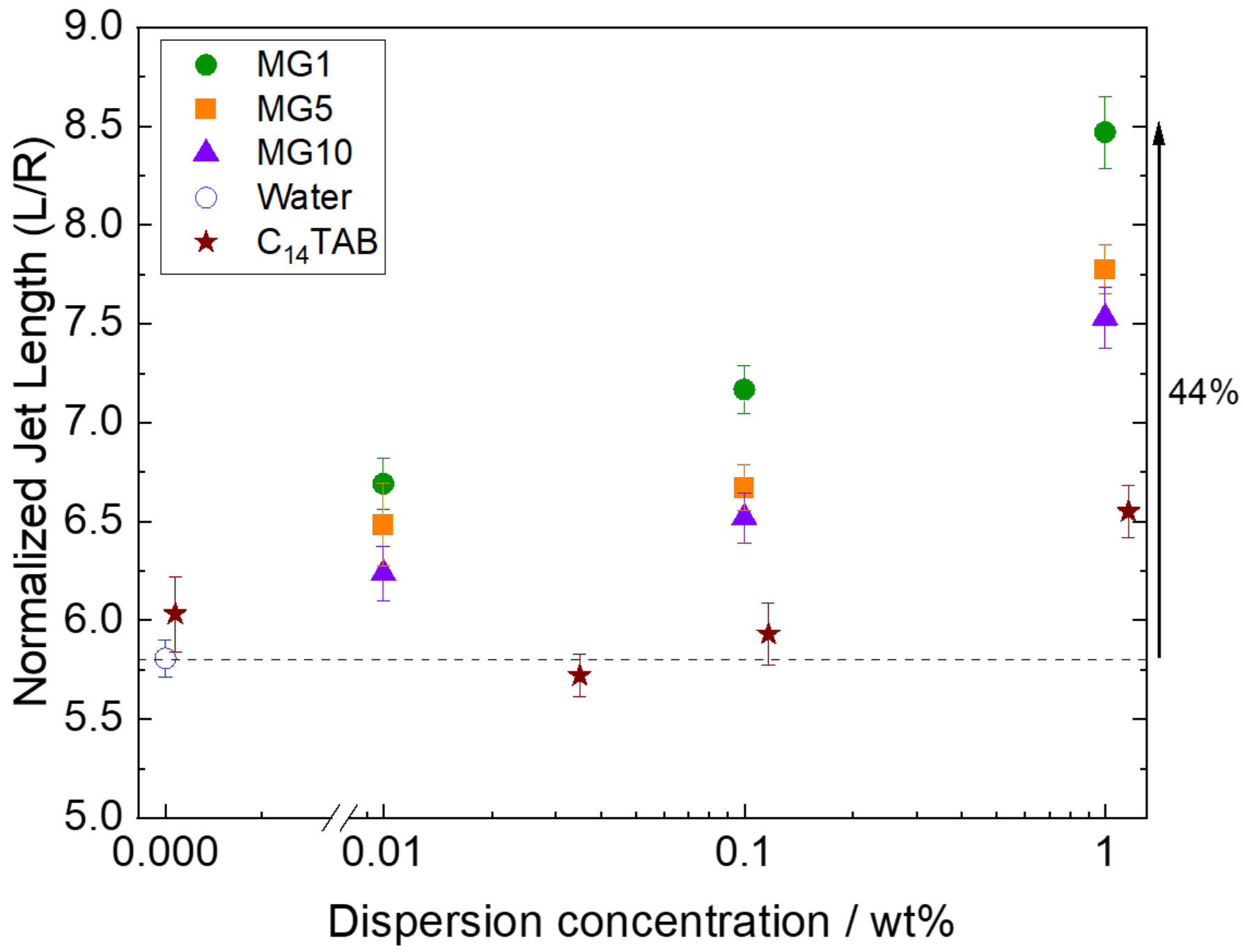

Figure R4: **Effect of concentration and interfacial stabilizer type on normalized jet length.** Maximum jet length normalized by the droplet's initial baseline (L/R) as a function of liquid concentration. Data are shown for pure water (concentration = 0, also shown with the dashed line), C_{14}TAB solutions at four different concentrations of about 0.01, 0.3, 1 and 10 CMC, and microgel dispersions with varying stiffness (different cross-linkerdensities): MG1, MG5, and MG10, across their respective aqueous concentrations.

stretching [see Fig. R5]. In contrast, microgels exhibit extremely large adsorption (desorption) energies at fluid interfaces, such that their removal from the interface on the millisecond timescale of SAW-driven jetting is highly unlikely compared to molecular surfactants. Therefore, microgel-stabilized jets remain smoother, indicative of a more mechanically robust interface that resists deformation even under extreme strain rates.

Thus, the pronounced mismatch between surfactant adsorption–desorption timescales and SAW-induced deformation rates provides a consistent explanation for why conventional surfactants fail to provide lasting stabilization, while soft microgels remain effective.

We have added the following discussion in SI:

'*To clarify the separation of timescales between interfacial deformation rates ($1/\tau_{\text{Jet}}$) and surfactant adsorption-desorption kinetics (τ_{ad}), we estimate the deformation rate using the jet diameter ($h \sim 0.2$ mm) and jetting velocity, yielding $\dot{\gamma} \approx u_0/h \approx 5000\ s^{-1}$ and a deformation timescale of $\tau_{\text{Jet}} \sim 1/\dot{\gamma} \approx 0.2$ ms.*

In contrast, cationic surfactants like C_{14}TAB and CTAB have adsorption-desorption times (τ_{ad}) on the order of seconds to minutes ($\tau_{\text{ad}} \sim 1$s). Oscillating-drop experiments [5] show that while surfactant exchange rates increase with frequency, the re-equilibration timescale remains slower than the rapid interfacial deformation. This mismatch suggests that, under SAW-driven jetting occurring on millisecond timescales, surfactant monolayers cannot replenish fast enough between deformation events, leading to dynamic surfactant depletion instead of maintaining a uniformly covered, equilibrated layer. Experimental observations further support this: surfactant-stabilized jets, such as those with C14TAB, exhibit more irregular deformations, indicating that the interface cannot withstand rapid stretching [see Fig. S7]'

> **Referee #1:** Comments 5: In the scaling analysis, could the authors clarify whether surface elasticity and surface viscosity at the microgel-laden air-water interface play a role in jet stability? Since the microgels coat the jet surface and modify interfacial rheology, this aspect warrants further discussion. In addition, this scaling argument assumes velocity is given, so the relation is descriptive rather than predictive. I suggest the authors clarify the assumptions and limitations.

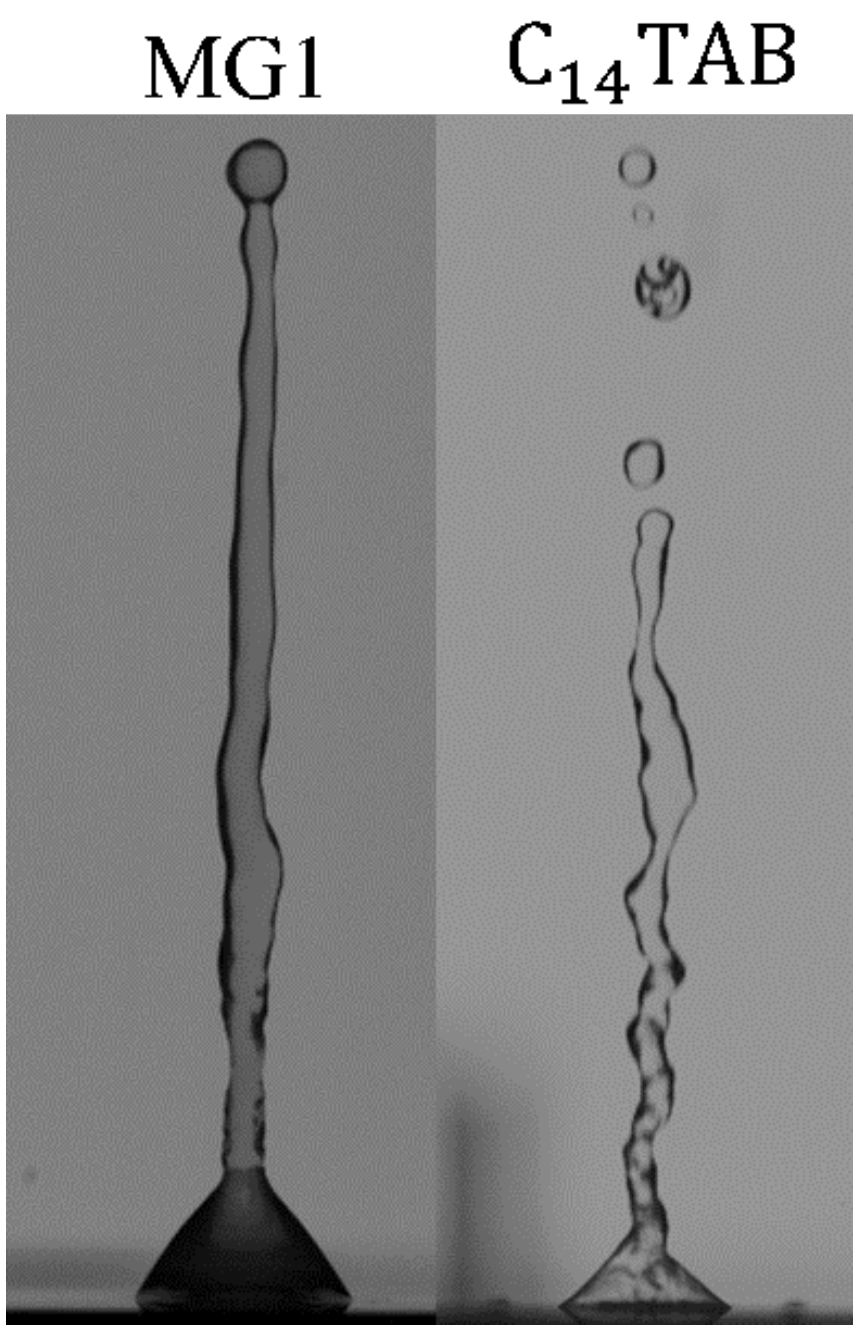

Figure R5: **Effect of surfactant C_{14}TAB on jet stabilization compared to microgels.** Representative snapshots illustrating the jet length for (left) 1wt% microgel dispersions with 1% crosslink density (MG1) and (right) for the cationic surfactant C_{14}TAB at concentrations well above the critical micelle concentration (CMC). The microgel-laden interface sustains a significantly longer and more stable jet. In contrast, the C_{14}TAB jet exhibits pronounced, irregular, and extreme interfacial deformations, indicating that the surfactant layer cannot efficiently smooth perturbations or withstand extensional stresses.

Reply:

We thank the Referee for this insightful comment. In the context of the SAW-driven jetting process, the interfacial area of a sessile droplet undergoes significant expansion as the interface evolves from an initial area of approximately:

$$A_0 \sim \frac{1}{2}\pi R^2$$

to that of a cylindrical jet with surface area

$$A \sim \pi h L.$$

The corresponding areal strain can therefore be estimated as

$$\epsilon = \frac{\Delta A}{A_0} = \frac{A}{A_0} - 1 \approx \frac{2hL}{R^2} - 1. \quad (1)$$

Using typical experimental values for the jet diameter ($h \sim 0.2$ mm), droplet base radius ($R \sim 1$ mm), and jet length ($L/R \approx 8$), the interfacial strain reaches over 200% [see Eq. 1], placing the system well beyond the elastic regime. While microgel stiffness indeed plays an important role in determining interfacial rheological properties [see our response to Comment 7], surface elasticity typically persists only at strains below approximately 10%. At larger strains, the microgel network leaves the elastic regime and undergoes a yielding, such that surface elasticity can no longer be treated as a well-defined property. Under such extreme and rapid interfacial deformations, surface elasticity does not remain a controlling parameter during jet elongation. Additionally, a low Capillary number ($\mathrm{Ca} = \mu u_0/\gamma \approx 0.01$, $\mu \sim \mathcal{O}(10^{-3}\ Pa.s)$, $u_0 \sim \mathcal{O}(1\ m\,s^{-1})$ and $\gamma \sim \mathcal{O}(10^{-1}\ Nm^{-1})$) indicates that viscous effects are negligible compared to surface tension. Therefore, the surface tension γ is the relevant interfacial parameter in the scaling analysis.

We agree with the reviewer that the derived scaling relation is descriptive rather than fully predictive, as the jet velocity is taken as a given parameter. Predicting the jet velocity in SAW-driven jetting is itself a complex problem, as it depends on the coupling between acoustic streaming, droplet geometry, and nonlinear interfacial dynamics, and is therefore beyond the scope of the present work. Tan *et al.* ref.(14) introduced an acoustic forcing term to the leading-order axisymmetric jet momentum balance equation and predicted that the characteristic jet velocity can be estimated as:

$$u_0 = \sqrt{2L_j\,(F_s^y - g)},$$

where F_s^y is the acceleration associated with acoustic streaming, g is the gravitational acceleration, and L_j is the jet length. The acoustic streaming force can be approximated as

$$F_s^{(y)} \approx \alpha_0 \beta\, \dot{\xi}_{x_3}^2\, \mathrm{Re}_A,$$

where α_0 is the acoustic attenuation coefficient, β is the coefficient of nonlinearity of the liquid, $\dot{\xi}_{x_3}$ is the surface displacement velocity of the transducer, and Re_A is the acoustic Reynolds number.

We note that this formulation is not strictly predictive either, as the jet velocity depends explicitly on the jet length L_j, which is itself an outcome of the jetting process. Nevertheless, the expression provides a useful order-of-magnitude estimate.

Using representative parameters reported in Ref. (14) for water droplets, we take a surface displacement amplitude of $\xi_0 \sim 10$ nm and a driving frequency of $f \sim 64$ MHz, which gives a surface displacement velocity of

$$\dot{\xi}_{x_3} = 2\pi f \xi_0 \sim 4\,\mathrm{m\,s^{-1}}.$$

Assuming a typical jet length of $L_j \sim 10$ mm and neglecting gravity compared to the acoustic streaming contribution, this leads to an estimated jet velocity on the order of

$$u_0 \sim 1\,\mathrm{m\,s^{-1}},$$

which is consistent with experimentally observed SAW-driven jet velocities reported in the literature.

Due to the complexity of the underlying acousto-fluidic physics, we use the experimentally observed initial jet velocity, set by tuning the input SAW power, typically around $u_0 \sim 1\,\mathrm{m\,s^{-1}}$, as the relevant input parameter for the scaling analysis.

We have added the following discussion in our revised SI.

‘*In the SAW-driven jetting process, the interfacial area of a sessile droplet expands significantly, evolving from an initial area of* $A_0 \sim \frac{1}{2}\pi R^2$ *to a cylindrical jet with surface area* $A \sim \pi h L$*. The corresponding areal strain is estimated as* $\epsilon = \frac{A}{A_0} - 1 \approx \frac{2hL}{R^2} - 1$*. Using typical experimental values for jet diameter (*$h \sim 0.2\,\mathrm{mm}$*), droplet base radius (*$R \sim 1\,\mathrm{mm}$*), and jet length (*$L/R \approx 8$*), the interfacial strain exceeds* 200%*, placing the system well beyond the elastic regime. While microgel stiffness is important for interfacial rheology, surface elasticity typically persists only at strains below* 10%*. At larger strains, the microgel network enters a yielding phase, and surface elasticity is no longer a well-defined property. Thus, under such rapid and extreme interfacial deformations, surface elasticity no longer governs the jet elongation.* ’

‘*Tan et al. [14] introduced an acoustic forcing term to the leading-order axisymmetric jet momentum balance equation and predicted that the characteristic jet velocity can be estimated as: ...*

...Due to the complexity of the underlying acousto-fluidic physics, we use the experimentally observed initial jet velocity, set by tuning the input SAW power, typically around $u_0 \sim 1\,\mathrm{m\,s^{-1}}$*, as the relevant input parameter for the scaling analysis.* ’

> **Referee #1:** Comments 6: In the scaling analysis, it seems to the reviewer that the kinetic energy from the SAW to the droplet should contribute to an increase in surface energy, which should be estimated as the difference between the surface energy of the jet and the surface energy of the droplet. Can the author clarify this point?

Reply: Dimensional analysis indicates that in this regime, both viscous and gravitational effects are negligible, as evidenced by the small Capillary and Bond numbers. Under these conditions, the jet dynamics are predominantly governed by the balance between inertia and surface tension.

The entire kinetic energy imparted by the SAWs to the droplet, which can be expressed as $\rho V u_0^2 \sim \rho \pi R^3 u_0^2/6$, where V is the volume of the droplet and R is its diameter, is assumed to be fully converted into surface energy during the formation of the cylindrical jet. The surface energy associated with the jet is given by $E_{\mathrm{surf}} \sim \gamma \pi h L$, where h is the jet diameter and L is its length. By equating the kinetic energy and surface energy ($E_{\mathrm{kin}} \sim E_{\mathrm{surf}}$), we obtain the scaling relation: $L/R \sim \rho u_0^2 R^2/(6\gamma h)$. This relation predicts jet lengths of the order of $\mathcal{O}(1\,\mathrm{cm})$, which aligns well with our experimental observations.

We have discussed this issue in our manuscript.

‘*Dimensional analysis [6,14] suggests that viscous and gravitational effects are negligible in this regime: the Capillary number,* $\mathrm{Ca} = \mu u_0/\gamma \sim 0.01$*, represents the ratio of viscous stress (*$\mu u_0/h$*) to capillary stress (*γ/h*) and indicates that viscous dissipation is weak; similarly, the Bond number,* $\mathrm{Bo} = \rho g h^2/\gamma \sim 0.001$*, compares gravitational stress (*$\rho g h$*) to capillary stress (*γ/h*) and indicates that gravity has minimal influence. Under these conditions, jet dynamics are governed primarily by a balance between inertia and surface tension. Hence, the entire kinetic energy imparted by the SAW to the droplet,* $E_{\mathrm{kin}} \sim \rho \pi R^3 u_0^2/6$*, is converted into the surface energy needed to form a cylindrical jet of diameter* h *and length* L*, given by* $E_{\mathrm{surf}} \sim \gamma \pi h L$*. Equating these two energy scales (*$E_{\mathrm{kin}} \sim E_{\mathrm{surf}}$*) yields the scaling relation*

$$\frac{L}{R} \sim \frac{\rho u_0^2 R^2}{6\gamma h}, \tag{2}$$

which predicts that, for fixed ρ*,* u_0*,* h*, and* R*, a reduction of the surface tension* γ *produces longer jets.* ’

> **Referee #1:** Comments 7: For Figure S3 in the supplementary materials, is there a specific reason why the plot is not presented in a logarithmic scale? Additionally, are there other rheological properties of the PINPAM microgel water solution, such as viscoelasticity, that could be reported? Comparing these rheological data to previous studies would provide valuable contexts.

Reply: We thank the Referee for the helpful suggestions. Figure S3 has been replotted using a logarithmic scale to provide a clearer representation of the data across the full range of shear rates.

Inspired by the Referee’s suggestion to report additional rheological properties, we have performed new interfacial rheology measurements, including both interfacial shear rheology [see Fig. R6] and interfacial dilatational elasticity [see Fig. R7], for PNIPAM microgel–laden air–water interfaces. Both measurements show that interfaces stabilized by softer microgels exhibit higher dilatational elastic moduli and shear storage moduli than those formed by stiffer microgels. This behaviour indicates that softer microgels form interfacial layers with an enhanced ability to store elastic energy and to resist changes in interfacial area and shear deformations. Furthermore, interfacial shear rheology measurements show that networks formed by softer microgels sustain higher strains before yielding or

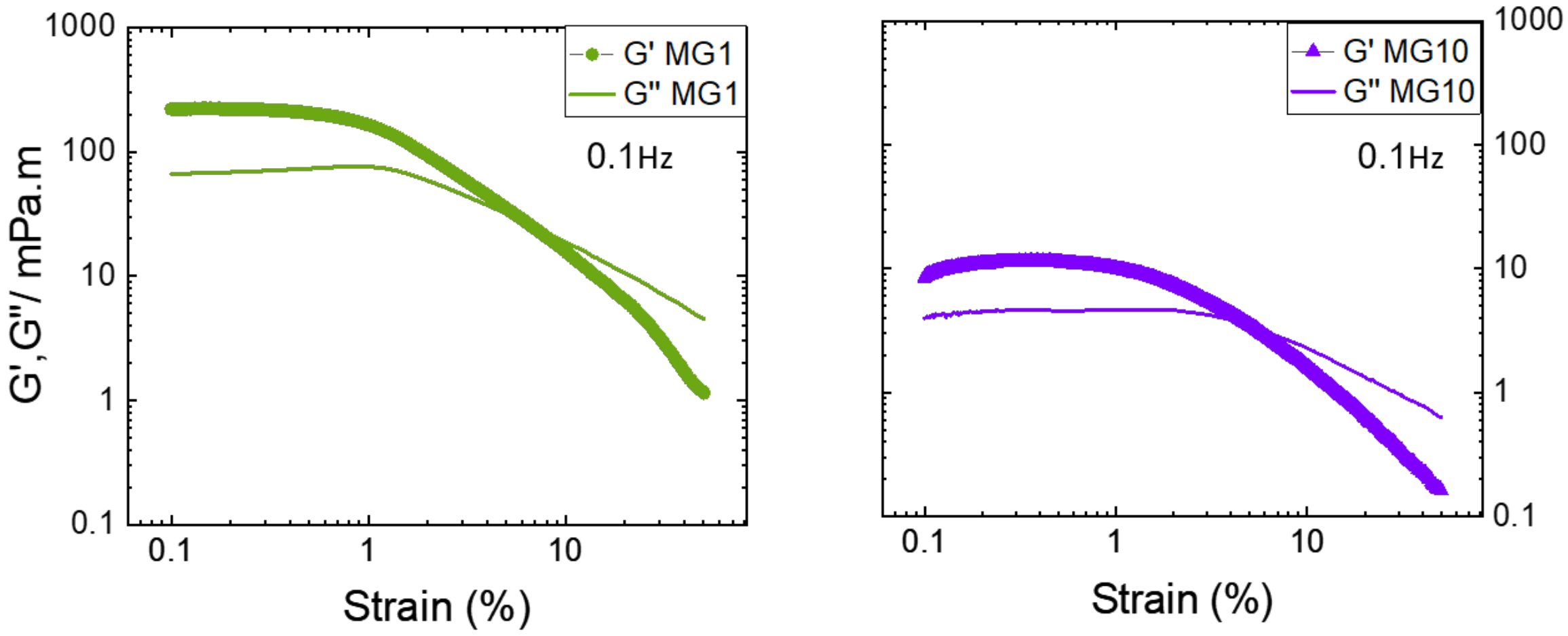

Figure R6: **Interfacial shear rheology of soft and stiff microgels.** The interfacial shear storage (G') and loss (G'') moduli were measured at a frequency of 0.1 Hz for soft and stiff microgels using 0.1 wt%. This concentration corresponds to a densely covered interface with an estimated surface pressure of $\sim 30\ \mathrm{mN\ m^{-1}}$. Measurements were performed at this representative coverage, as the rheological setup is not coupled to a Langmuir trough for independent control of surface pressure.

loss of mechanical integrity. This indicates that softer microgels form more resilient, better-connected interfacial networks, capable of accommodating larger deformations before mechanical failure.

However, we note that while these rheological measurements offer valuable insights into network robustness, they are not directly comparable to the jetting experiments. During SAW-driven jetting, the interface experiences extreme extensional strains, reaching over 200% (see Eq. 1) far beyond the deformation range typically encountered in standard interfacial rheology experiments. At such large strains, surface elasticity is no longer well-defined. Therefore, while interfacial rheology provides important information about the network's stability at smaller strains (below 10%), the jet stability in our system is ultimately governed by the interface's ability to maintain a low effective surface tension during rapid extension, rather than by its elastic moduli.

We have included this discussion in our main text.

'*This raises the question of whether jet stability is controlled by the viscoelastic properties [48, 49] of microgel-laden air–water interfaces, which are influenced by the softness of individual microgels. Supplementary interfacial rheology measurements, including interfacial shear [see Fig. S8] and dilatational experiments [see Fig. S9], show that interfaces stabilized by softer microgels exhibit higher dilatational elastic and shear storage moduli and sustain larger strains before yielding, indicating the formation of more resilient interfacial networks. However, these measurements probe the linear viscoelastic regime, typically at strains below 10%, whereas SAW-driven jetting subjects the interface to extreme extensional deformations exceeding 200%. Under such large, rapid extensions, the interfacial layer is driven far beyond the regime where small-strain elastic moduli remain well-defined. Consequently, jet stability in our system is governed primarily by the interface's ability to maintain a low effective surface tension during rapid elongation rather than by its small-strain viscoelastic moduli.* '

> **Referee #1:** Comments 8: In Figure 3, how can it be determined that the microgels fully cover the jet surface? From the images, it appears that the cross-link concentration of 10% covers more of the jet surface area compared to the 1% concentration. Could the authors elaborate on this observation?

Reply: Each PNIPAM microgel consists of a dense core and a less dense shell, which contains dangling polymers. Due to the finite spatial resolution of AFM, the shell regions cannot be directly resolved. As a result, the apparent gaps between the microgel particles in the AFM images do not indicate uncovered areas of the interface, but rather reflect unresolved shell regions.

Previous studies [Tatry *et al. J. Colloid Interface Sci.* 629 (228), 2023] on PNIPAM microgels at fluid interfaces have shown that at a surface pressure of approximately $5\ \mathrm{mN/m}$, the polymer shells of neighboring microgels

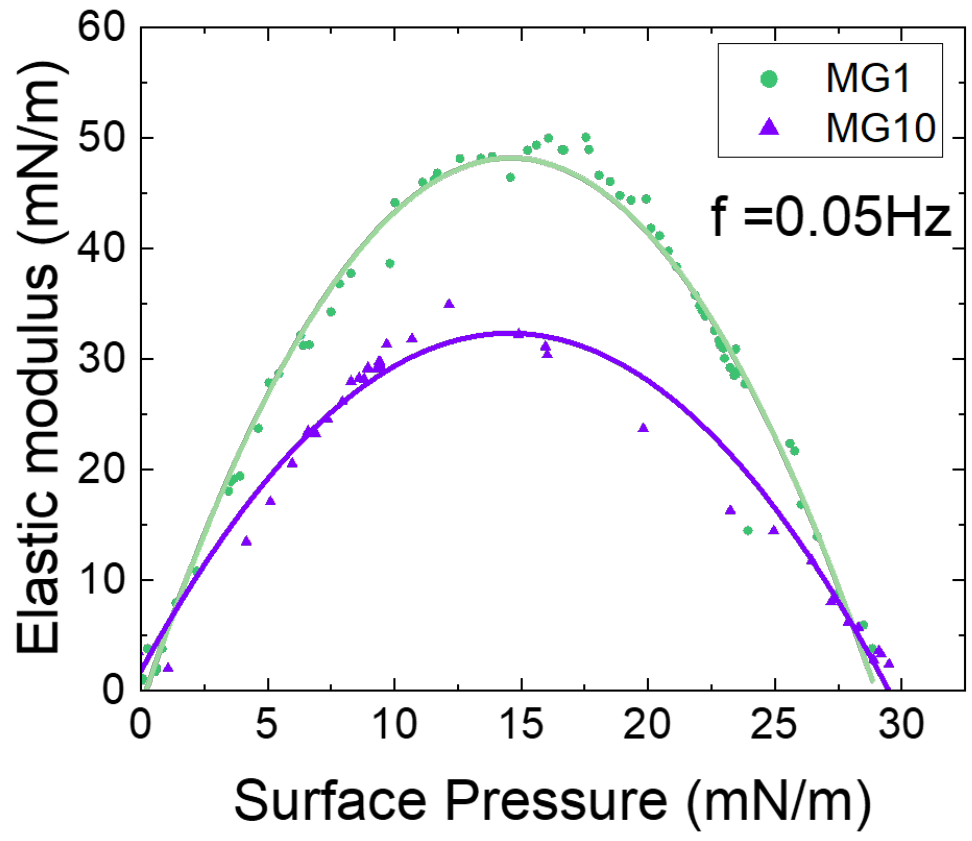

Figure R7: **Apparent dilatational elastic modulus as a function of surface pressure.** The elastic modulus, measured at the air–water interface using the oscillating drop method at a frequency of 0.05 Hz, is higher for the soft microgel (MG1) than the stiffer microgel (MG10).

are already in contact. Core-core contact typically occurs only at higher surface pressures, above 25 mN/m. To further support this, we have included additional AFM images of microgel monolayers prepared at 25 mN/m in the revised Supplementary Information [see Fig. R3]. In this compressed regime, where both shell-shell and core-core interactions are present, it becomes even more evident that individual soft microgels occupy a significantly larger interfacial area, nearly twice that of stiffer microgels, despite the higher particle number density of the latter.

Specifically, stiffer microgels (MG10) exhibit a smaller individual interfacial footprint compared to softer microgels (MG1), due to the greater deformability and lateral spreading of the softer microgels at the interface. Consequently, to maintain the same surface pressure (i.e., the same reduction in surface tension), a larger number of stiffer microgels must adsorb per unit area compared to softer ones. This key observation from the AFM images highlights that softer microgels spread more extensively at the interface and, therefore, require fewer particles to achieve the same reduction in surface tension.

We have included the following discussion in our SI.

'*The choice of a surface pressure of* 5 *mN/m is based on our simulation data, which suggests that, upon sufficient elongation, the surface tension of the air-water interface, including stiff microgels, reaches approximately 65 mN/m [see Fig. R2].*

...To further illustrate this distinction, we have included Langmuir–Blodgett images acquired at approximately 25 mN/m in the revised SI [see Fig. R3], which show the highly compressed interfacial state at the jet's initiation. In this compressed regime, where both shell-shell and core-core interactions are present, it becomes even more evident that individual soft microgels occupy a significantly larger interfacial area compared to stiffer microgels, despite the higher particle number density of the latter.

Specifically, stiffer microgels (MG10) exhibit a smaller individual interfacial footprint compared to softer microgels (MG1), due to the greater deformability and lateral spreading of the softer microgels at the interface. Consequently, to maintain the same surface pressure (i.e., the same reduction in surface tension), a larger number of stiffer microgels must adsorb per unit area compared to softer ones. This key observation from the AFM images highlights that softer microgels spread more extensively at the interface and, therefore, require fewer particles to achieve the same reduction in surface tension. '

> **Referee #1:** Comments 9: Could the authors clarify why a cross-link concentration of 1% can be used in experiments but not in simulations? Furthermore, how do the simulation results compare to the experimental findings?

Reply: Following Ref. [Camerin *et al. ACS Nano*, 13 (4548), 2019], the microgel network in our simulations is generated using patchy particles, where monomers carry two patches and crosslinkers carry four, with all particles otherwise behaving identically. This model is commonly used for simulating microgel systems. The monomers and

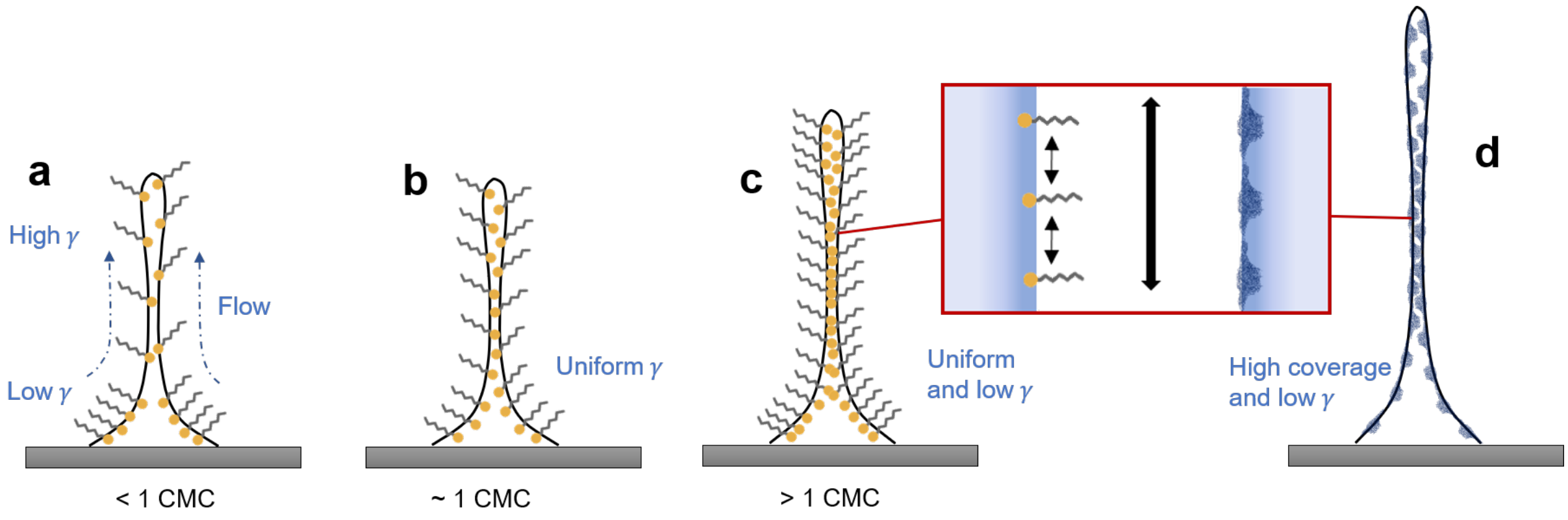

Figure R8: **Schematic representation of jet from surfactant solutions and microgel dispersion.** (a) At low surfactant concentration (sub-CMC), interfacial extension generates gradients in surfactant coverage, driving Marangoni flows that transiently counteract breakup and extend jet length. (b) At concentrations close to 1 CMC, the interface is more uniformly distributed, eliminating surface tension gradients and suppressing Marangoni flows, which leads to reduced jet stability and shorter jets. (c) At surfactant concentrations well above the CMC, the interface is saturated and the equilibrium surface tension—and corresponding Laplace pressure—are reduced. However, the surfactant layer remains molecular and lacks the ability to form a mechanically resilient interfacial network, limiting stabilization under strong extensional deformation. (d) In contrast, microgel dispersions form deformable, polymer-rich interfacial layers that can stretch, rearrange, and maintain a low effective surface tension during rapid jet elongation, leading to significantly enhanced jet stability.

crosslinkers are initially placed randomly, and as a result, the formation of a 1% cross-linked network is slower compared to networks with 5% or 10% crosslinking, due to the lower concentration of crosslinkers, which leads to delayed bond formation.

In response to the Referee's suggestion, we have also prepared microgels with a 1% crosslinker density and repeated our simulations to investigate the effects of lower crosslinker concentrations. We find that while the 1% crosslinker concentration results in a slight reduction in surface tension compared to the 2% crosslinker concentration [see Fig. R2], the overall trend and our conclusions remain valid.

We have added this discussion to our revised manuscript.

'*We have also prepared microgels with a 1% crosslinker density and repeated our simulations to investigate the effects of lower crosslinker concentrations. We find that while the 1% crosslinker concentration results in a slight reduction in surface tension compared to the 2% crosslinker concentration [see Fig. 6], the overall trend and our conclusions remain valid.* '

> **Referee #1:** Comments 10: In Figure 4, the authors show a schematic for the surfactant-laden jet ejection. Can the authors add a schematic to contrast it with the microgel-laden jet ejection, which is the main topic in the current study?

Reply: In response to this valuable suggestion, we have updated Fig. 4 in the revised manuscript (Fig. R8 here) to include a schematic contrasting the surfactant-laden jet ejection with the microgel-laden jet ejection, which is the focus of the current study. This addition enhances the comparison between the two systems.

2. Reply to Referee #2

> **Referee #2:** The manuscript by Razavi et al. describes how soft PNIPAM microgels at liquid–air interfaces suppress capillary breakup in surface-acoustic-wave driven water jets. The authors combine experiments, simulations, and a scaling model to show that low-crosslink density microgels extend jet length by up to $\sim 44\%$ compared to controls. They hypothesize that softer, less crosslinked microgels form entangled interfacial networks that remain cohesive under strong elongational flows, thereby resisting surface tension–driven breakup.
>
> The study is exciting and convincingly demonstrates the potential of engineering liquid interfaces with microgel networks. At the same time, the mechanistic picture remains experimentally not fully complete: while the theoretical model supports the hypothesis, direct validation is limited, and other mechanistic explanations cannot yet be entirely ruled out given the complexity of the problem. Further experiments directly addressing these alternative possibilities will therefore be essential to firmly establish the proposed mechanism.

Reply: We thank the Referee for the positive evaluation of our work and for recognizing the exciting potential of engineering liquid interfaces with microgel networks. We also appreciate the Referee's insightful comments regarding the mechanistic aspects of the study. In response, we have conducted additional experiments and simulations to further validate our hypothesis and explore alternative mechanisms.

> **Referee #2:** Comment 1: Flow profile within the jet: To understand how soft microgels extend jetting, it is necessary to resolve the flow profile inside the jet. Which forces act on the liquid interface, and at which positions do elongational stresses peak? Such measurements or simulations would be critical to link hydrodynamic stresses directly to interfacial stabilization.

Reply: We thank the Referee for the insightful suggestion. To resolve the flow profile near the air-water interface, we conducted explicit solvent simulations using Dissipative Particle Dynamics (DPD). Specifically, we simulated two setups under identical stretching conditions: one with stiff microgels (MG10) and another with soft microgels (MG1) [see Fig. R9]. We divide the xz-plane into small sub-boxes and average the velocity over all particles within each sub-box.

In the MG10 system, where the microgels disentangle from one another, the water interface becomes exposed to air at the center of the simulation box, leading to a significant change in the velocity profile [see Fig. R9c]. We observed that the z-component velocity is roughly twice as high at the center, where the microgels no longer cover the interface, compared to the regions where microgels remain attached. This z-component velocity is also negative, indicating an inward flow of water due to the stretching of the interface. The other velocity components (v_x and v_y) were found to be significantly smaller and close to zero on average.

In contrast, the MG1 system, where the soft microgels remain entangled and fully cover the interface, exhibits a more uniform velocity distribution near the interface [see Fig. R9d]. This uniformity helps stabilize the jet and prevents the onset of instability, as the microgels act to dampen the velocity gradients that would otherwise contribute to the jet's breakdown [see Fig. R9f]. We therefore conclude that a uniform extensional flow field (MG1) allows the jet to maintain its cylindrical shape, whereas a strongly non-uniform z-velocity (MG10) amplifies Rayleigh-Plateau-like instabilities and accelerates jet breakup.

To quantify the elongational stress peak, we calculated the elongational stress via pressure anisotropy, $\Delta p_{xz} = P_{zz} - P_{xx}$, following rheological models [O'Connor *et al.*, *Phys. Rev. Lett.* 121 (047801), 2018]. The elongational stress near the interface is directly related to surface tension [Heitmeier *et al.*, *Phys. Rev. Lett.* 136 (068203), 2026] via $\gamma = L_z \, \Delta p_{xz}$, where L_z is the sub-volume height. We found that when the interface is exposed to air (as in MG10), the local surface tension increases by approximately 10 mN/m compared to the microgel-covered interface [see Fig. R10]. In contrast, with soft microgels (MG1), surface tension remains stable over a wide range of stretching, maintaining a stable interface.

Although direct velocity measurements during jetting are experimentally inaccessible, we quantified instability by measuring the neck diameter evolution (Fig. R11), inspired by the previous experimental work [Utada *et al.* *Phys. Rev. Lett.* 99 (094502), 2007]. For jetting from a pure water droplet, the neck diameter vanishes around 1.75ms, indicating rapid instability, whereas for the soft microgel-laden jet (MG1), breakup is delayed until approximately 4ms [see Fig. R11]. This significant delay highlights that soft microgel-covered interfaces are more resilient to elongational stresses.

We have discussed this in our main manuscript.

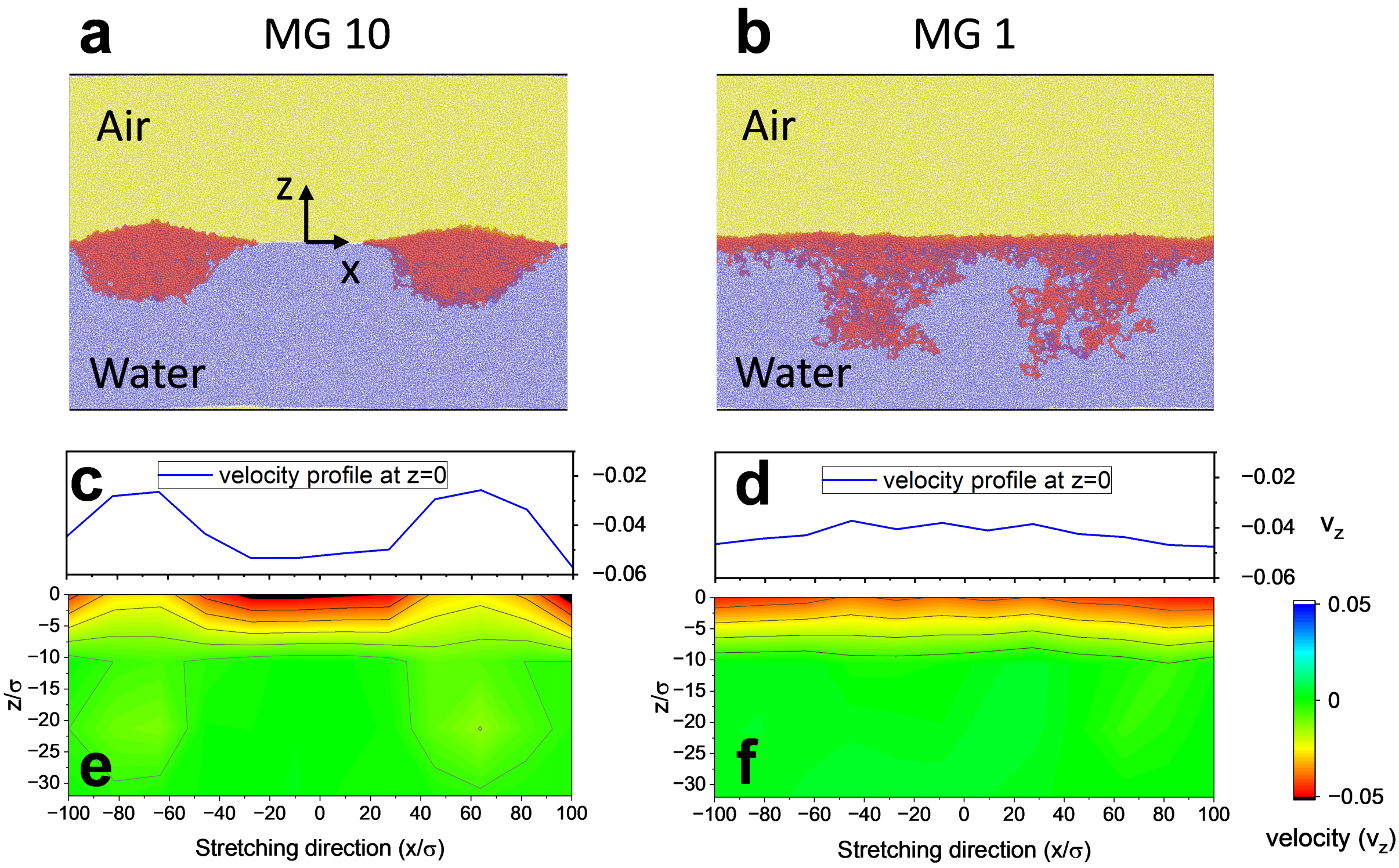

Figure R9: **Flow profiles of stiff (MG10) and soft (MG1) microgels during stretching of the interface.** (a and b) Representative snapshots of stiff (MG10) and soft (MG1) microgels at the air-water interface during stretching at $t/\tau_0 = 46000$. At this stage, stiff microgels (MG10) disentangle, exposing the central region of the interface to the bare air-water surface. In contrast, soft microgels (MG1) remain entangled and fully cover the interface. (e and f) Variation of the z-component velocity of water in the xz-plane. The lines represent regions of constant velocity, where the velocity magnitude remains the same between consecutive lines. The velocity anisotropy along the stretching axis is pronounced near the interface for stiff microgels (MG10) and diminishes with increasing distance from the interface. In contrast, for soft microgels (MG1), the z-component velocity remains position-independent along the stretching axis. The magnitude of the z-component velocity is indicated by the color code. (c and d) Spatial variation of the z-component water velocity along the stretching axis, immediately adjacent to the interface (at z=0). For MG10, the velocity at the center (where water is exposed to air) is approximately twice as large as in regions where the microgels remain attached to the interface. For MG1, the z-component velocity remains uniform across the interface due to the complete coverage by the soft microgels. Consequently, a uniform negative z-velocity (MG1) allows the expanding jets to roughly keep their cylindrical shape, whereas a strongly nonuniform z-velocity (MG10) provokes Rayleigh-Plateau-like instabilities and jet breaking. Simulation Parameters: The flow profile is obtained by time-averaging over 5000 DPD time steps, starting from $t/\tau_0 = 46000$, followed by 500 independent simulation runs.

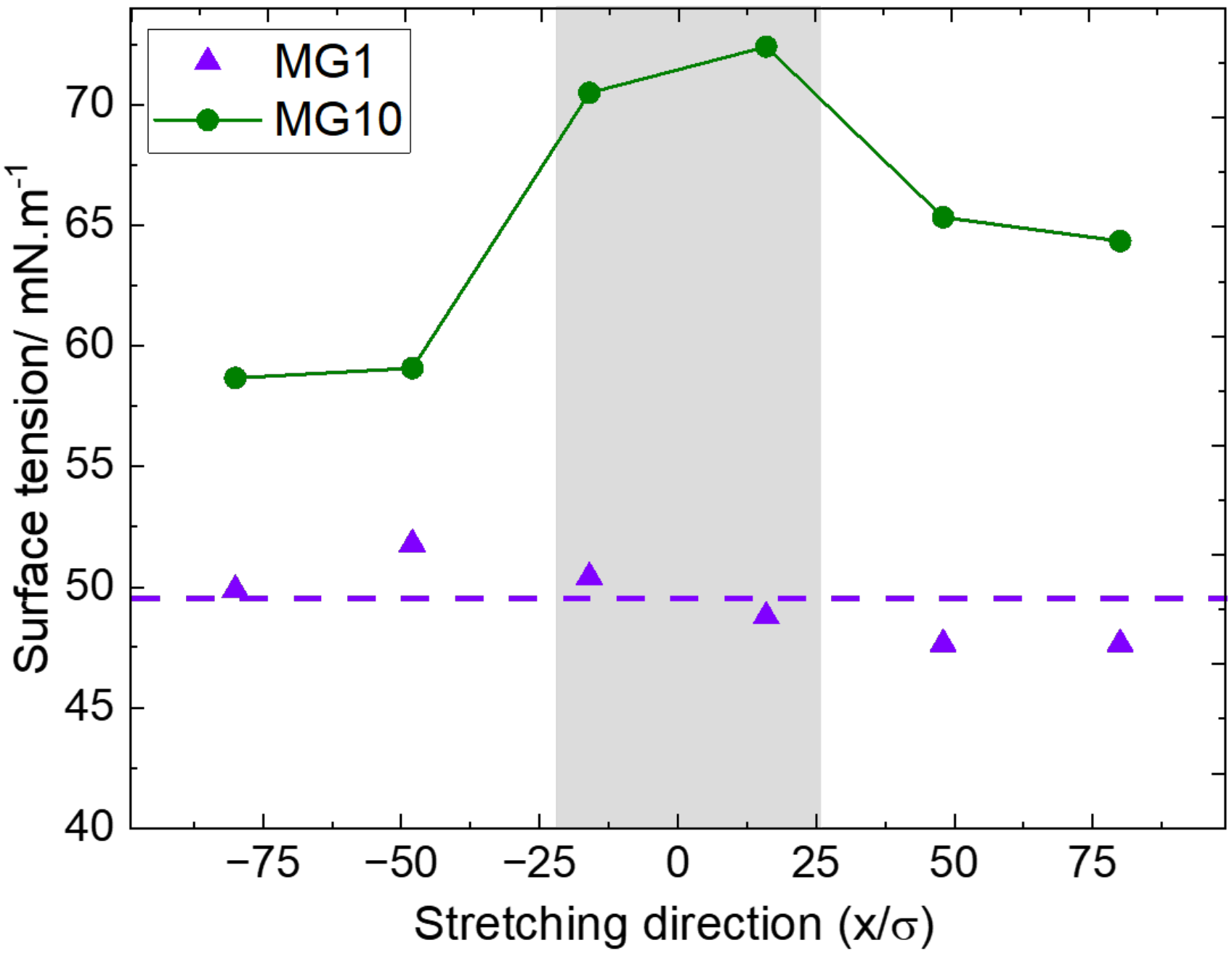

Figure R10: **Surface tension profiles of stiff (MG10) and soft (MG1) microgels during stretching of the interface.** Spatial variation of surface tension along the stretching axis. The grey region indicates the position of the bare air-water interface for stiff microgels. The surface tension at the center reaches the pure air-water interface value ($\gamma \approx 70\mathrm{mN/m}$) for stiff microgels, while for soft microgels, the surface tension remains almost position-independent at $\gamma \approx 49\mathrm{mN/m}$, which is significantly lower than the pure air-water surface tension.

'*Our simulations further reveal that the disentanglement of stiff microgels (MG10) during interfacial extension leads to pronounced inhomogeneities in the interfacial flow .....* '

We have updated our SI accordingly.

'*To quantify the elongational stress peak, we calculated the elongational stress via pressure anisotropy,* $\Delta p_{xz} = P_{zz} - P_{xx}$*, following rheological models [6]. The elongational stress near the interface is directly related to surface tension via* $\gamma = L_z\,\Delta p_{xz}$*, where* L_z

... This significant delay highlights that soft microgel-covered interfaces are more resilient to elongational stresses. '

> **Referee #2:** Comment 2: Interfacial rheology in shear and dilation: Interfacial rheology is the natural experimental method to test the proposed mechanism. Shear and dilatational rheology could determine whether softer microgels increase not only the elasticity of the interface but also its yielding and failure thresholds. Establishing a correlation between rheological parameters (elastic modulus, dilatational modulus, relaxation time, yield stress) and jet extension would strongly support the authors' explanation.

Reply: We agree with the reviewer that interfacial rheology provides an important experimental framework to probe the mechanical response of microgel-laden interfaces. Inspired by the Referee's suggestion to report additional rheological properties, we have conducted further interfacial rheology measurements, including both interfacial shear rheology and interfacial dilatational elasticity, for PNIPAM microgel–laden air–water interfaces (Figs. R6 and R7). Both measurements show that interfaces stabilized by softer microgels exhibit higher dilatational elastic moduli and shear storage moduli than those formed by stiffer microgels. This behaviour indicates that softer microgels form interfacial layers with an enhanced ability to store elastic energy and to resist changes in interfacial area and shear deformations. Furthermore, interfacial shear rheology measurements show that networks formed by softer microgels sustain higher strains before yielding or loss of mechanical integrity. This indicates that softer microgels form more resilient, better-connected interfacial networks, capable of accommodating larger deformations before mechanical failure.

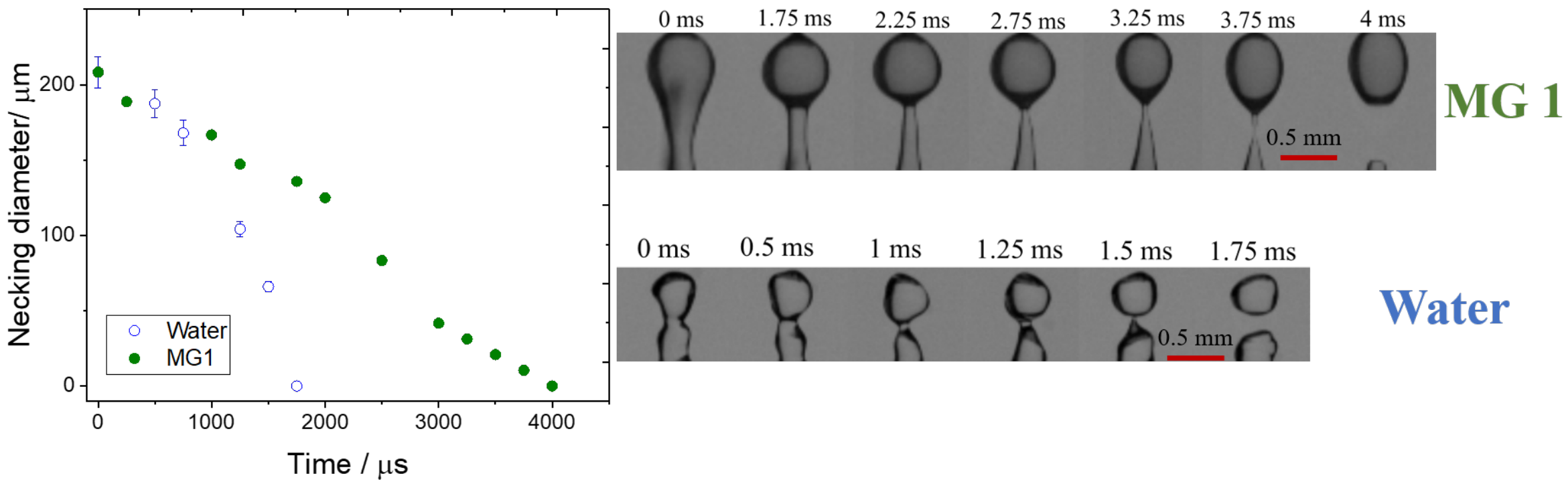

Figure R11: **Necking dynamics at the onset of jet breakup for pure water and soft microgels (MG1).** (Left) Time evolution of the neck diameter measured after the jet reaches its maximum length, illustrating the development of the Rayleigh–Plateau instability. (Right) Corresponding snapshots of the necking process. The pure water jet breaks into droplets when the neck diameter collapses at approximately (≈ 1.75ms), whereas, for soft microgels (MG1), the neck diameter remains nonzero until approximately 4ms. This delayed necking reflects the enhanced resistance of the microgel-stabilized interface to capillary breakup, resulting in a longer jet prior to fragmentation.

However, we note that while these rheological measurements offer valuable insights into network robustness, they are not directly suited to explain the complex dynamics of comparable to the jetting experiments. During SAW-driven jetting, the interface experiences extreme extensional strains, reaching over 200% (see Eq. 3), far beyond the deformation range typically encountered in standard interfacial rheology experiments. At such large strains, surface elasticity is no longer well-defined.Therefore, while interfacial rheology provides important information about the network's stability at smaller strains (below 10%), the jet stability in our system is ultimately governed by the interface's ability to maintain a low effective surface tension during rapid extension, rather than by its elastic moduli.

We have included this discussion in our manuscript.

'*This raises the question of whether jet stability is controlled by the viscoelastic properties [48, 49] of microgel-laden air–water interfaces, which are influenced by the softness of individual microgels. Supplementary interfacial rheology measurements, including interfacial shear [see Fig. S8] and dilatational experiments [see Fig. S9], show that interfaces stabilized by softer microgels exhibit higher dilatational elastic and shear storage moduli and sustain larger strains before yielding, indicating the formation of more resilient interfacial networks. However, these measurements probe the linear viscoelastic regime, typically at strains below 10%, whereas SAW-driven jetting subjects the interface to extreme extensional deformations exceeding 200%. Under such large, rapid extensions, the interfacial layer is driven far beyond the regime where small-strain elastic moduli remain well-defined. Consequently, jet stability in our system is governed primarily by the interface's ability to maintain a low effective surface tension during rapid elongation rather than by its small-strain viscoelastic moduli.* '

> **Referee #2:** Comment 3: Microgel monolayer rupture upon jet formation: The microgel monolayer likely ruptures at the onset of jet formation, as the rapid creation of new interface cannot be sustained solely by a pre-adsorbed layer. It remains an open question whether microgels dynamically adsorb to freshly created interfaces during jetting, and whether differences in adsorption kinetics between soft and stiff particles contribute to the observed stabilization. Addressing this point could help disentangle whether network elasticity or fast adsorption is the dominant factor.

Reply: We thank the Referee for raising this insightful question. Indeed, the rapid area expansion during jet formation may not be fully sustained by a pre-adsorbed microgel monolayer alone. To better understand this process, we estimate the areal strain involved during the jetting process.

In the SAW-driven jetting process, the interfacial area of a sessile droplet undergoes significant expansion as the interface evolves from an initial area of approximately: $A_0 \sim \frac{1}{2}\pi R^2$ to the surface area of a cylindrical jet: $A \sim \pi h L$. The corresponding areal strain can therefore be estimated as:

$$\epsilon = \frac{\Delta A}{A_0} = \frac{A}{A_0} - 1 \approx \frac{2hL}{R^2} - 1. \tag{3}$$

Using typical experimental values for the jet diameter $h \sim 0.2$ mm, droplet base radius $R \sim 1$ mm, and jet length $L/R \approx 8$, the interfacial strain reaches over 200%.

At the same time, our experiments and simulations demonstrate that pre-adsorbed soft microgels undergo substantially larger interfacial deformation than stiff microgels, enabling them to maintain a reduced effective surface tension during rapid stretching. Importantly, the estimated areal strain during jet formation ($\sim 200\%$, see Eq. 3) is compatible with the observed ability of individual soft microgels to laterally stretch by approximately a factor of two, as evidenced by our AFM measurements and consistent with previous reports of center-to-center distance at different surface pressures [Robertson *et al.*, *Soft Matter* 22, 2026]. This agreement supports the conclusion that deformation of pre-adsorbed microgels makes a significant and physically plausible contribution to jet stabilization, even under extreme extensional conditions.

Finally, we emphasize that the term "network elasticity" should be interpreted cautiously in this highly dynamic, non-equilibrium system. Under the extreme extensional strains involved in SAW-driven jetting, the interface does not behave as a static elastic network. Rather, stabilization arises from the ability of the microgel-laden interface to continuously adapt, and deform, while maintaining a low effective surface tension under rapid interfacial deformation.

We have added the following discussions to our manuscript.

'*To better understand the rupture of the microgel monolayer upon jet formation, we estimate the areal strain during the jetting process. The interfacial area of a sessile droplet undergoes significant expansion, reaching roughly* 200%*, as the interface evolves into a cylindrical jet. At the same time, our experiments and simulations demonstrate that pre-adsorbed soft microgels undergo substantially larger interfacial deformation than stiff microgels, enabling them to maintain a reduced effective surface tension during rapid stretching. Notably, the estimated areal strain during jet formation (*$\sim$ 200%*) is compatible with the observed ability of individual soft microgels to laterally stretch by approximately a factor of two, as evidenced by our AFM measurements and consistent with previous reports of center-to-center distance at different surface pressures [51]. Given that jet formation occurs on a rapid timescale, limiting the contribution from diffusive replenishment of particles from the bulk, this agreement supports the conclusion that deformation of pre-adsorbed microgels provides a physically consistent mechanism contributing to jet stabilization under extreme extensional conditions.* '

> **Referee #2:** Comment 4: Surfactants: On page 1, the authors write: "Yet in SAW-driven systems, interfacial deformation occurs so rapidly that surfactants desorb before providing lasting stabilization." This is a central statement, but no citation is provided. References or data supporting this claim should be included to justify the argument.

Reply: We thank the Referee for the insightful suggestion. Our claim that extreme deformation rates driven by surface acoustic waves (SAWs) cause conventional surfactants to desorb is supported by a clear separation of timescales between the interfacial deformation rate ($1/\tau_{\mathrm{Jet}}$) and surfactant adsorption–desorption kinetics (τ_{ad}). Estimating the deformation rate from the jet diameter ($h \sim 0.2\,\mathrm{mm}$) and jetting velocity, we find $\dot{\gamma} \approx u_0/h \approx 5000\mathrm{s}^{-1}$, which yields a deformation timescale $\tau_{\mathrm{Jet}} \sim 1/\dot{\gamma} \approx 0.2\,\mathrm{ms}$.

In contrast, cationic surfactants like C14TAB and CTAB have characteristic adsorption–desorption times (τ_{ad}) typically on the order of seconds to minutes ($\tau_{\mathrm{ad}} \sim 1\mathrm{s}$). Oscillating-drop experiments (Yang *et al.*, *J. Colloid Interface Sci.* 537, 2019) show that while surfactant exchange rates increase with frequency, the timescale for surfactant re-equilibration remains longer than that of rapid interfacial deformation. This mismatch between the adsorption–desorption timescale and the deformation timescale suggests that under SAW-driven jetting conditions, which occur on millisecond timescales, the surfactant monolayer cannot replenish itself fast enough between successive deformation events. As a result, the interface undergoes dynamic surfactant depletion, rather than maintaining a uniformly covered, equilibrated surfactant layer during jet elongation. This behavior is further supported by experimental observations of jet morphology: surfactant-stabilized jets, like those with C_{14}TAB, exhibit more irregular interfacial deformations, indicating that the interface cannot sustain a resistance under rapid stretching [see Fig. R4]. In contrast, microgels exhibit extremely large adsorption (desorption) energies at fluid interfaces, such that their removal from the interface on the millisecond timescale of SAW-driven jetting is highly unlikely compared to molecular surfactants. Therefore, microgel-stabilized jets remain smoother, indicative of a more mechanically robust interface that resists deformation even under extreme strain rates.

Thus, the pronounced mismatch between surfactant adsorption–desorption timescales and SAW-induced deformation rates provides a consistent explanation for why conventional surfactants fail to provide lasting stabilization, while soft microgels remain effective.

We have added the following discussion to our manuscript:

'*Furthermore, C_{14}TAB is a cationic surfactant and is therefore expected to exhibit relatively fast adsorption kinetics compared to neutral or anionic surfactants [47]. The fact that even such a fast-adsorbing surfactant fails to achieve stabilization comparable to that of microgels strongly supports our central conclusion: polymer-based interfacial stabilizers perform better under the rapid, high-strain interfacial dynamics relevant to jet formation [see Fig. S7].* '

We have added the following discussion to our SI:

'*To clarify the separation of timescales between interfacial deformation rates ($1/\tau_{\rm Jet}$) and surfactant adsorption-desorption kinetics ($\tau_{\rm ad}$), we estimate the deformation rate using the jet diameter ($h \sim 0.2$ mm) and jetting velocity, yielding $\dot{\gamma} \approx u_0/h \approx 5000\ s^{-1}$ and a deformation timescale of $\tau_{\rm Jet} \sim 1/\dot{\gamma} \approx 0.2$ ms.*

In contrast, cationic surfactants like C_{14}TAB and CTAB have adsorption-desorption times ($\tau_{\rm ad}$) on the order of seconds to minutes ($\tau_{\rm ad} \sim 1$s). Oscillating-drop experiments [5] show that while surfactant exchange rates increase with frequency, the re-equilibration timescale remains slower than the rapid interfacial deformation. This mismatch suggests that, under SAW-driven jetting occurring on millisecond timescales, surfactant monolayers cannot replenish fast enough between deformation events, leading to dynamic surfactant depletion instead of maintaining a uniformly covered, equilibrated layer. Experimental observations further support this: surfactant-stabilized jets, such as those with C14TAB, exhibit more irregular deformations, indicating that the interface cannot withstand rapid stretching [see Fig. S7] '

> **Referee #2:** Comment 5: Range of tested systems: The authors tested three microgels with crosslinking densities from 1 mol-% to 10 mol-% and used CTAB as a reference. It would be useful to discuss whether the effect could be further enhanced by using ultra-low crosslinked microgels, which might form even more deformable and entangled networks. Similarly, a comparison to other surfactant systems, such as non-ionic surfactants or polymeric stabilizers (e.g. linear PNIPAM), could help distinguish whether the observed trends are unique to microgels or reflect a more general property of polymeric interfaces.

Reply: We thank the Referee for the insightful comment. To address this, we have performed additional experiments with the cationic surfactant C_{14}TAB at concentrations up to 10 times the critical micelle concentration (CMC). Despite the substantial increase in surfactant concentration, C_{14}TAB was still significantly less effective than PNIPAM microgels in extending jet length. This indicates that the limited stabilization provided by molecular surfactants is not due to insufficient surface coverage but rather due to the intrinsic limitations of surfactant layers under extreme extensional deformation. We highlight that C_{14}TAB, as a positively charged surfactant, is expected to exhibit relatively fast adsorption kinetics compared to neutral or negatively charged surfactants. The fact that even such a fast-adsorbing surfactant fails to achieve the stabilization performance of soft microgels supports our conclusion that polymer-based stabilizers offer a fundamentally different and more robust mechanism for stabilizing interfaces under rapid deformation.

Additionally, we have conducted experiments using linear PNIPAM $M = 40000$ as a polymeric stabilizer. These results show that linear PNIPAM can produce jets with lengths reaching approximately 95% of those stabilized by the softest microgels (MG1). This demonstrates that the enhanced jet stability is not unique to microgels as particulate objects but instead arises from the soft, deformable, and extensible nature of polymer chains at the interface, which enables surface tension to remain low during rapid elongation.

While we currently do not have access to ultra-low crosslinked microgels, we anticipate that even more deformable systems, such as ultra-low-crosslinked microgels or linear polymers with longer chain lengths, would further enhance jet stability. These systems would increase interfacial extensibility, thereby delaying capillary breakup and further improving stabilization.

'*We also performed experiments using linear PNIPAM ($M = 40000$) as a polymeric stabilizer, producing jets with lengths approximately 95% of those stabilized by the softest microgels (MG1). This shows that enhanced jet stability is not exclusive to microgels as particulate stabilizers, but arises from the soft, deformable, and extensible nature of polymer chains at the interface, which helps maintain low surface tension during rapid elongation. As such, we anticipate that more deformable systems, such as ultra-low crosslinked microgels or linear polymers with longer chain lengths, could further improve jet stability.* '

3. Reply to Referee #3

> **Referee #3:** The manuscript by Razavi et al. presents acoustically driven nozzle-free jets by tuning the softness of microgels that can control interfacial resilience and extend jet length up to a considerable amount. The work is of potential interest for surfactant-free, biocompatible strategy in case of precision printing and needle-free drug delivery. It is well written with detailed experimental and simulation methods and results.
>
> The manuscript is suitable for publication after the authors address my following comments.

Reply: We thank the Referee for their positive feedback and for recommending our manuscript for publication in Nature Communications. We are happy to address the comments below.

> **Referee #3:** Comment 1: It is mentioned, microgels are constructed from a self-assembled network using patchy particles. Equilibration of the 1% cross-linked network is slow. The authors should explain a bit more how the microgels are self-assembled and why equilibration of 1% network takes long time.

Reply: Following Ref. [61], the microgel network in our simulations is generated using patchy particles, where monomers carry two patches and crosslinkers carry four, with all particles otherwise behaving identically. This model is commonly used for simulating microgel systems. The monomers and crosslinkers are initially placed randomly, and as a result, the formation of a 1% cross-linked network is slower compared to networks with 5% or 10% crosslinking, due to the lower concentration of crosslinkers, which leads to delayed bond formation.

In response to the Referee's suggestion, we have also prepared microgels with a 1% crosslinker density and repeated our simulations to investigate the effects of lower crosslinker concentrations. We find that while the 1% crosslinker concentration results in a slight reduction in surface tension compared to the 2% crosslinker concentration [Fig. R2], the overall trends observed in the simulations remain consistent.

We have added this discussion in our revised manuscript.

'*We have also prepared microgels with a 1% crosslinker density and repeated our simulations to investigate the effects of lower crosslinker concentrations. We find that while the 1% crosslinker concentration results in a slight reduction in surface tension compared to the 2% crosslinker concentration [see Fig. R2], the overall trend and our conclusions remain valid.* '

> **Referee #3:** Comment 2: Considering Fig. 6, if the authors simulate long time, will MG2 reach to the results of MG5 and MG10, the bare air-water tension?

Reply: Thank you for raising this question. Indeed, if we simulate MG2 for a longer time, the microgels will eventually disentangle from each other, leading to a reduction in coverage of the air-water interface. As a result, the surface tension is expected to approach the value observed for MG5 and MG10 at long times [see Fig. R12].

> **Referee #3:** Comment 3: Comparing Fig. 6 and S2, Fig. 6 shows low tension to bare tension with time, whereas Fig S2 shows opposite. If simulation is done in reverse directions from lateral stretched box to backwards (Fig. 5, from b to a and d to c), what will be the surface tension dynamics?

Reply: We thank the Referee for this insightful question. The apparent discrepancy between Figs. S2 and 6 arises from the fundamentally different experimental and simulation protocols, initial conditions, and timescales.

Fig. S2 shows pendant-drop measurements in which surface tension decreases over time as microgels adsorb, spread, and reorganize at the interface, ultimately reaching equilibrium after several hundred seconds. In contrast, Fig. 6 shows simulation results starting from a fully equilibrated, microgel-covered interface, which is actively stretched. This stretching exposes bare air-water regions, leading to an increase in surface tension.

Additionally, we performed reverse simulations, where microgels are forced to entangle from a disentangled state, showing finite hysteresis [see Fig. R13]. This is consistent with recent experimental work Abgarjan, *et al.*, Soft Matter, 21 (5030-5044) (2025)].

We have added this clarification in our figure caption.

'***Surface tension of microgel dispersions measured via pendant drop experiments.*** *Surface tension of PNIPAM dispersions at different cross-linker content (i.e., stiffness) and various concentrations of a) 0.01 wt%, b) 0.1 wt%,*

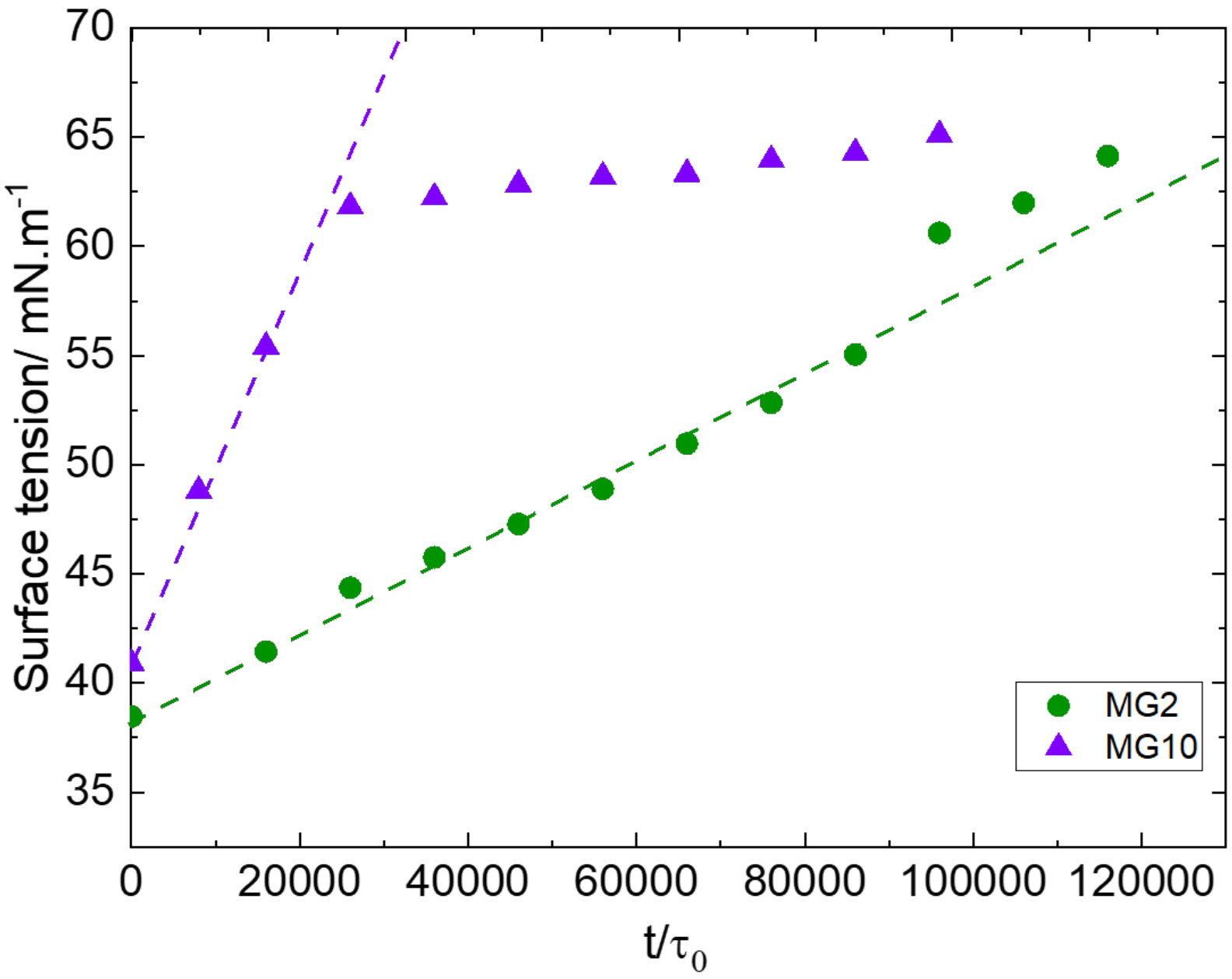

Figure R12: **Simulated Surface Tension Dynamics at Long Times.** The time evolution of dynamic surface tension for microgels with different cross-linker densities (MG1 and MG10). The plot demonstrates how varying the cross-linker concentration affects the rate of surface tension evolution, emphasizing the contrasting behaviors of soft (low cross-linker density) and stiff (high cross-linker density) microgels during interfacial stretching. Notably, due to their smaller size, these microgels approach the equilibrium air-water surface tension at long times.

and c) 1 wt%. The time scale varies due to different kinetics at various concentrations. Jetting occurs at around 20 ms. '

We have also added the following text in the SI.

'*The surface tension of microgel dispersions with varying cross-linker content was measured using a drop-shape analyzer. A pendant droplet was formed, and the surface tension was recorded at different dispersion concentrations of 0.01, 0.1, and 1 wt%, corresponding to Fig. S2a, S2b and S2c, respectively. The results show that softer microgels (MG1) reduce the surface tension more rapidly than the stiffer microgels (MG5 and MG10). Furthermore, increasing the microgel concentration accelerates the reduction in surface tension.*'

> **Referee #3:** Comment 4: The time scales in Fig. 6 and S2 are different. Is it possible to map the time-scale for simulation and experimental results?

Reply: Thank you for your question. As previously discussed, the timescales in Fig. S2 and Fig. 6 correspond to fundamentally different physical processes and therefore cannot be directly mapped onto one another.

Inspired by pioneering simulation works [Camerin *et al.* *ACS Nano* 13 (4548–4559), 2019, Bochenek *et al.* *Nature Commun.* 13 (3744), 2022], we studied 10 nm microgels up to a time of 0.1 μs, which is the current computational limit. This simulated microgel size is approximately 50 times smaller than the experimental microgels (500 nm). This size difference leads to two key consequences: (1) Experimental microgels cover 50^2 larger surface area compared to the simulated microgels, and (2) larger microgels, with longer dangling polymers, exhibit stronger entanglement effects. According to de Gennes' Nobel-winning work [de Gennes *Macromolecules* 9 (587-593), 1976], the disentanglement time scales with the cube of the polymer length. Therefore, for our 50 times larger experimental microgels, the disentanglement time would increase by a factor of 50^3. Consequently, if we could simulate microgels 50 times larger, the disentanglement time would be approximately:

$$0.1\,\mu\text{s} \times 50^3 \approx 12\,\text{ms}. \tag{4}$$

Notably, our experimental jetting experiments were conducted for 10 ms, suggesting that the timescales of our simulations align well with the experimental results, providing a consistent comparison between the two.

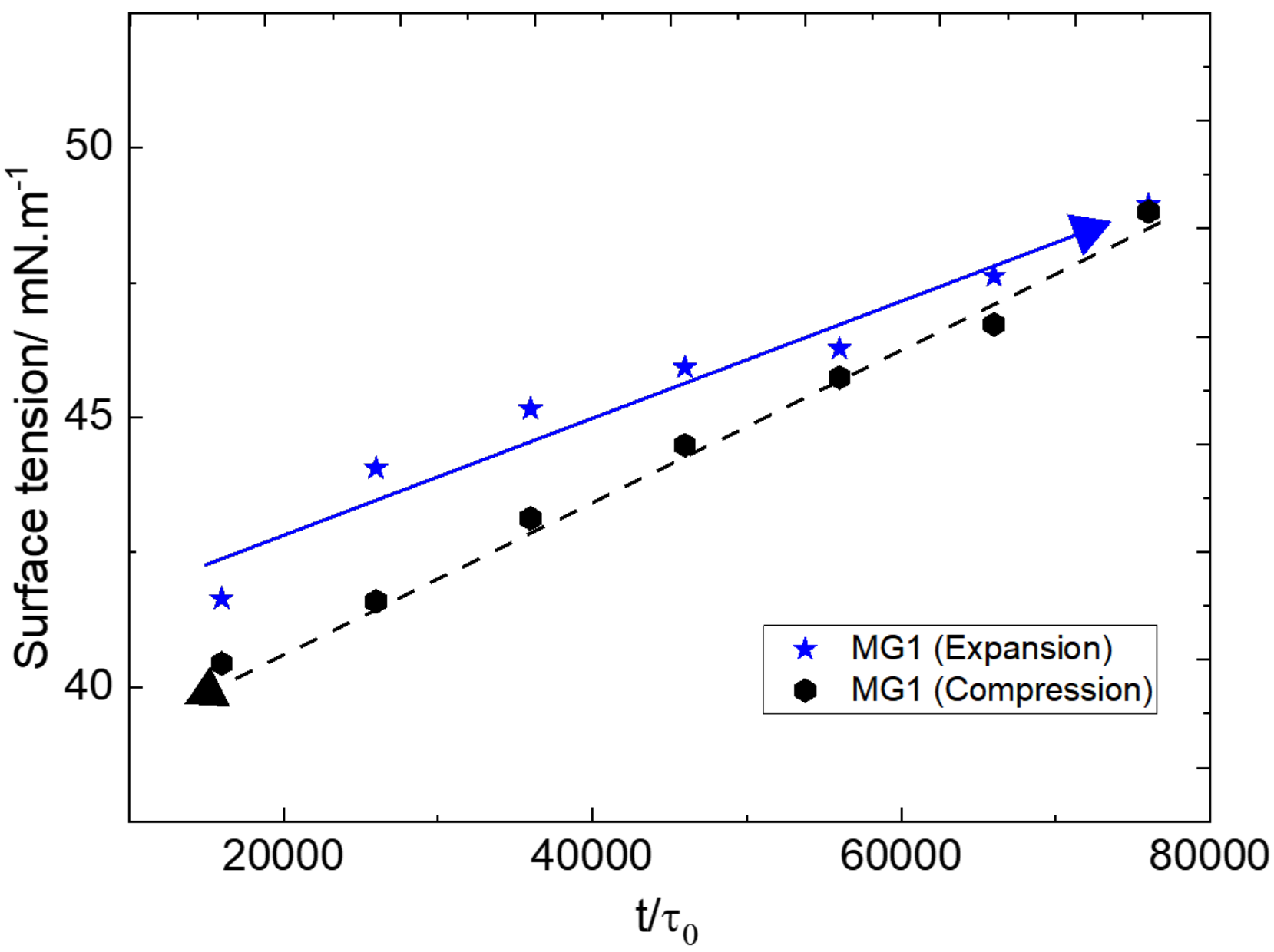

Figure R13: **Simulated Surface Tension Dynamics of MG1 at both Expansion and Compression.** The time evolution of the dynamic surface tension for microgels with the lowest cross-linker density (MG1) shows a slight discrepancy between compression and expansion.

We have included the following discussion in our SI.

'*Inspired by pioneering simulation works [10, 11], we studied 10 nm microgels up to a time of 0.1 μs, which is the current computational limit. This simulated microgel size is approximately 50 times smaller than the experimental microgels (500 nm). This size difference leads to two key consequences: (1) Experimental microgels cover* 50^2 *larger surface area compared to the simulated microgels, and (2) larger microgels, with longer dangling polymers, exhibit stronger entanglement effects. According to de Gennes' Nobel-winning work [12], the disentanglement time scales with the cube of the polymer length. Therefore, for our 50 times larger experimental microgels, the disentanglement time would increase by a factor of* 50^3. *Consequently, if we could simulate microgels 50 times larger, the disentanglement time would be approximately:*

$$0.1\,\mu s \times 50^3 \approx 12\,ms. \tag{5}$$

Notably, our experimental jetting experiments were conducted for 10 ms, suggesting that the timescales of our simulations align well with the experimental results, providing a consistent comparison between the two. '

> **Referee #3:** Comment 5: The authors should mention these works, Höfken et al. Rheologica Acta, 2025, doi.org/10.1007/s00397-025-01503-0 and Ruiz-Franco et al. Adv. Mater. Interfaces 2025, 12, 2500242, DOI: 10.1002/admi.202500242, regarding the change of viscoelastic properties of the interface through adsorption of microgels.

Reply: Thanks for suggesting these references. We are glad to add these references with discussions.

'*This raises the question of whether jet stability is controlled by the viscoelastic properties [48, 49] of microgel-laden air–water interfaces..* '

> **Referee #3:** (Remarks on code availability): There is no code available in that link.

Reply: We sincerely apologize for the inconvenience. The link provided was valid for only one month. We have now ensured that the link remains active throughout the revision process. Upon final acceptance, we will make the code publicly available in accordance with the standard requirements for Nature Communications.

1. Reply to Referee #1

> **Referee #1:** I thank the authors taking time to address my concerns, and I appreciate the authors' additional clarification regarding the interfacial rheology and the proposed scaling law. The analysis of the elastic modulus and interfacial properties of the microgels is substantial and helpful. I recommend publication of the manuscript as it is.

We are grateful for the Referee's recommendation for publication in Nature Communications.

2. Reply to Referee #2

> **Referee #2:** The revised manuscript provides additional experiments and simulations that strengthen the overall presentation. Nevertheless, several aspects of the mechanistic interpretation remain insufficiently resolved. In particular, the current discussion largely attributes jet stabilization to interfacial effects associated with deformable microgel networks. However, alternative mechanisms related to bulk polymer physics are not fully considered and should be addressed more carefully.
>
> The dimensional analysis assumes that viscous effects are negligible based on a small Capillary number calculated using the shear viscosity of water. However, this argument may not fully capture the rheology of polymer-containing systems. It is well established that dilute polymer solutions can exhibit substantially enhanced extensional viscosities while maintaining shear viscosities close to that of the solvent (Anna and McKinley, J. Rheol. 45, 115–138, 2001; Campo-Deaño and Clasen, J. Non-Newtonian Fluid Mech. 165, 1688–1699, 2010). Under strong elongational flows, such as those occurring during jet formation and thinning, even small concentrations of polymer can generate elastic stresses that delay capillary breakup. In this context, considering only the shear viscosity may not fully exclude potential viscoelastic contributions arising from extensional stresses in polymer-containing systems.
>
> A related aspect concerns the experiments with linear PNIPAM. Linear polymer chains typically exhibit comparatively weak interfacial viscoelastic responses relative to particle-laden interfaces such as microgel monolayers. Nevertheless, the manuscript reports that linear PNIPAM produces jets with lengths reaching approximately 95% of those obtained with the softest microgels. This observation raises the question of whether the observed jet stabilization can be attributed solely to interfacial network mechanics, or whether contributions from polymeric material present in the bulk phase—such as dissolved polymer chains or microgels dispersed in the bulk—might also play a role.
>
> The comparison to pure water and a molecular surfactant provides a useful reference point. However, given the similar jet lengths observed for linear PNIPAM and soft microgels, it may also be helpful to consider and discuss additional polymeric controls. In particular, a clearer description of the linear PNIPAM conditions (e.g., concentration) and their relation to the microgel experiments could help place these observations in context. Clarifying the relative contributions of interfacial microgel mechanics and possible bulk polymer effects would substantially strengthen the mechanistic interpretation of the results.

Reply: We thank the Referee for raising the point regarding the role of bulk polymer physics, in particular elastic properties, in jet formation.

As mentioned by the Referee, dilute high-molecular-weight linear polymers (e.g., $\sim$1 MDa PEO, as studied by Anna and McKinley, J. Rheol., 45, 115–138, 2001, and Campo-Deaño and Clasen, J. Non-Newtonian Fluid Mech., 165, 1688–1699, 2010) can exhibit significantly enhanced extensional viscosities under elongational flow. This behavior arises because polymer chains undergo a coil-to-stretch transition when the applied strain rate exceeds a critical value $\dot{\gamma}_c \sim 1/\tau$, where τ is the molecular relaxation time of the polymer chains [de Gennes, J. Chem. Phys. 60, 5030–5042 (1974), Perkins et al. Science 276, 2016 (1997), Calabrese et al. Phys. Rev. X 15, 021025 (2025)]. This transition generates elastic stresses that can eventually dominate over viscous stresses, leading to a pronounced increase in extensional viscosity. Hence, for significant polymer stretching and a considerable extensional viscosity, the Weissenberg number $\mathrm{Wi} = \dot{\gamma}\tau$ must reach $O(1)$.

We use linear PNIPAM as a limiting case that represents maximum softness relative to cross-linked PNIPAM microgels. However, the linear PNIPAM employed here, with a relatively low molecular weight ($M_w \approx 40$ kDa), has a very short relaxation time (on the order of microseconds in the dilute limit). At the applied strain rates ($\dot{\gamma} \sim 5000\mathrm{s}^{-1}$, as discussed in our supporting information), this corresponds to a Weissenberg number of Wi ~ 0.005, which is well below $O(1)$. Under these conditions, the polymer chains remain close to their equilibrium coil state, and no appreciable coil-to-stretch transition is expected. As a result, extensional viscosity does not play a significant role. Microgels, however, due to their internal cross-linked network, are unable to undergo the coil-to-stretch transition seen in linear polymers. Thus, the development of significant bulk extensional stresses is also limited under the present experimental conditions (1 wt%). Instead, the dynamics of jet formation are primarily influenced by interfacial effects, such as dynamic surface tension, which are much more prominent in these systems.

Compared to PEO (Gaillard et al., J. Fluid Mech., 1005, A10, 2025), which has a surface tension of around 65 mN/m, both linear PNIPAM and PNIPAM microgels exhibit a steady-state surface tension of about 43 mN/m due to their greater amphiphilicity. Additionally, the linear PNIPAM control experiments were performed at the same

diluted concentrations (1 wt%) as the microgel dispersions to enable direct comparison. The observation that jets formed by linear PNIPAM reach $\sim 95\%$ of that observed for the softest microgels stems from their similar surface activity and their fast surface tension kinetics. This indicates that bulk viscoelastic effects are not dominant in either of the studied systems.

We have revised the conclusion accordingly:

'*Furthermore, in the present study, the low-molecular-weight linear PNIPAM ($M_w = 40$ kDa) at dilute concentrations (1 wt%) suggests that jet dynamics are governed primarily by interfacial effects, similar to the case of microgels. In contrast, surface-active high-molecular-weight linear polymers can introduce additional bulk contributions, such as extensional viscosity [52–56], alongside their interfacial effects.*'

3. Reply to Referee #3

> **Referee #3:** The Authors addressed all points I mentioned. Therefore I recommend the manuscript for publication.

Thank you very much for the recommendation for publication in Nature Communications.

1. Reply to Referee #2

> **Referee #2:** I thank the authors for their detailed response and for the additional discussion of possible bulk extensional polymer stresses. This has improved the manuscript. However, I remain concerned that the main mechanistic issue is not fully resolved.
>
> Suitable polymeric reference systems are important for the interpretation, particularly given indications that non-crosslinked PNIPAM can produce jet lengths comparable to those obtained with the softest microgels. This directly bears on whether the effect is specific to soft microgel network mechanics or instead reflects a broader polymer-mediated interfacial stabilization mechanism.
>
> The current comparison between microgels and molecular surfactants is valuable, but it does not provide a sufficiently complete reference framework to separate the roles of polymer chemistry, interfacial adsorption, possible bulk contributions, and microgel architecture. Additional polymeric reference systems would be needed to support the broader mechanistic conclusion.
>
> I therefore remain unconvinced that the manuscript, in its present form, supports its central mechanistic conclusion that soft microgel networks are the key mechanism of jet stabilization.

Reply: We thank the Referee for the continued careful assessment of our manuscript. Suitable polymeric reference systems are essential for disentangling the respective roles of polymer chemistry, interfacial activity, bulk polymer effects, and microgel architecture, and for clarifying the broader design principle underlying our work. To address this concern, we have performed additional control experiments using linear polyethylene glycol (PEG) solutions and compared them directly with linear PNIPAM (LPNIPAM) and the soft microgel system (MG1). The corresponding results are presented in Fig. R1. These controls do not isolate every contribution independently; however, they provide an important framework for distinguishing the role of PNIPAM-mediated interfacial activity from the mere presence of dissolved polymer chains.

PEG was selected as a polymeric reference because, unlike PNIPAM, it exhibits only weak surface activity. The 35 kDa PEG is particularly relevant because its molecular weight is comparable to that of the LPNIPAM used in this study ($M_w \approx 40$ kDa). Previous studies [L. T. Lee, B. Jean, and A. Menelle, *Langmuir* 1999, 15 (9), 3267-3272 and T. Gilányi, I. Varga, M. Gilányi, R. Mészáros, *J. Colloid Interface Sci.* 2006, 301, 2, 428-435] indicate that linear PNIPAM exhibits an interfacial adsorption (surface excess) at the air–water interface approximately three times greater than that of PEG, owing to its greater amphiphilicity.

As shown in Fig. R1(a), 35 KDa PEG solution at a concentration of 1 wt% exhibits equilibrium surface tensions of approximately 62 mN/m, whereas both LPNIPAM and MG1 reduce the surface tension to approximately 43 mN/m. Correspondingly, Fig. R1(b,c) shows that the PEG solution produces substantially shorter jets than either LPNIPAM or MG1. Despite having a molecular weight comparable to that of LPNIPAM, PEG-35 kDa behaves much more similarly to water than to the PNIPAM-based systems. These observations demonstrate that a dissolved polymer with comparatively weak surface activity does not reproduce the pronounced jet stabilization observed for the PNIPAM-based systems. They further establish that polymer chemistry and the associated interfacial activity play an essential role in the observed stabilization.

We further note that the Weissenberg-number analysis presented in our previous response indicates that the 40 kDa LPNIPAM chains remain far from the coil-to-stretch transition under the experimental conditions (Wi $\ll 1$). The same conclusion applies to PEG-35 kDa. Consequently, generic bulk-polymer effects, such as enhanced extensional viscosity, are not expected to dominate the jet dynamics in either system.

The similar jet lengths obtained for LPNIPAM and MG1 indicate that PNIPAM-mediated interfacial activity plays an important role in the stabilization mechanism, consistent with the similarly low surface tensions ($\sim$ 43 mN/m) measured for both systems. However, the two systems are expected to maintain this low surface tension through different interfacial processes during jetting. Based on the microgel concentration (1 wt%) and diffusion coefficient ($D_0 \sim 10^{-12}\ m^2/s$), the characteristic time for microgels to travel its average interparticle distance ($\sim 1\mu$m) is on the order of seconds, several orders of magnitude longer than the jetting timescale ($\sim$1 ms), whereas 40 kDa LPNIPAM chains reach newly created interface on microsecond timescales (Fig. R2). Thus, microgel stabilization is governed predominantly by the pre-adsorbed interfacial layer, whereas LPNIPAM can additionally stabilize the interface through rapid adsorption during extension.

Our DPD simulations further show that the deformable, crosslinked microgel architecture maintains a compliant interfacial layer during stretching through persistent interactions between the dangling chains of neighboring

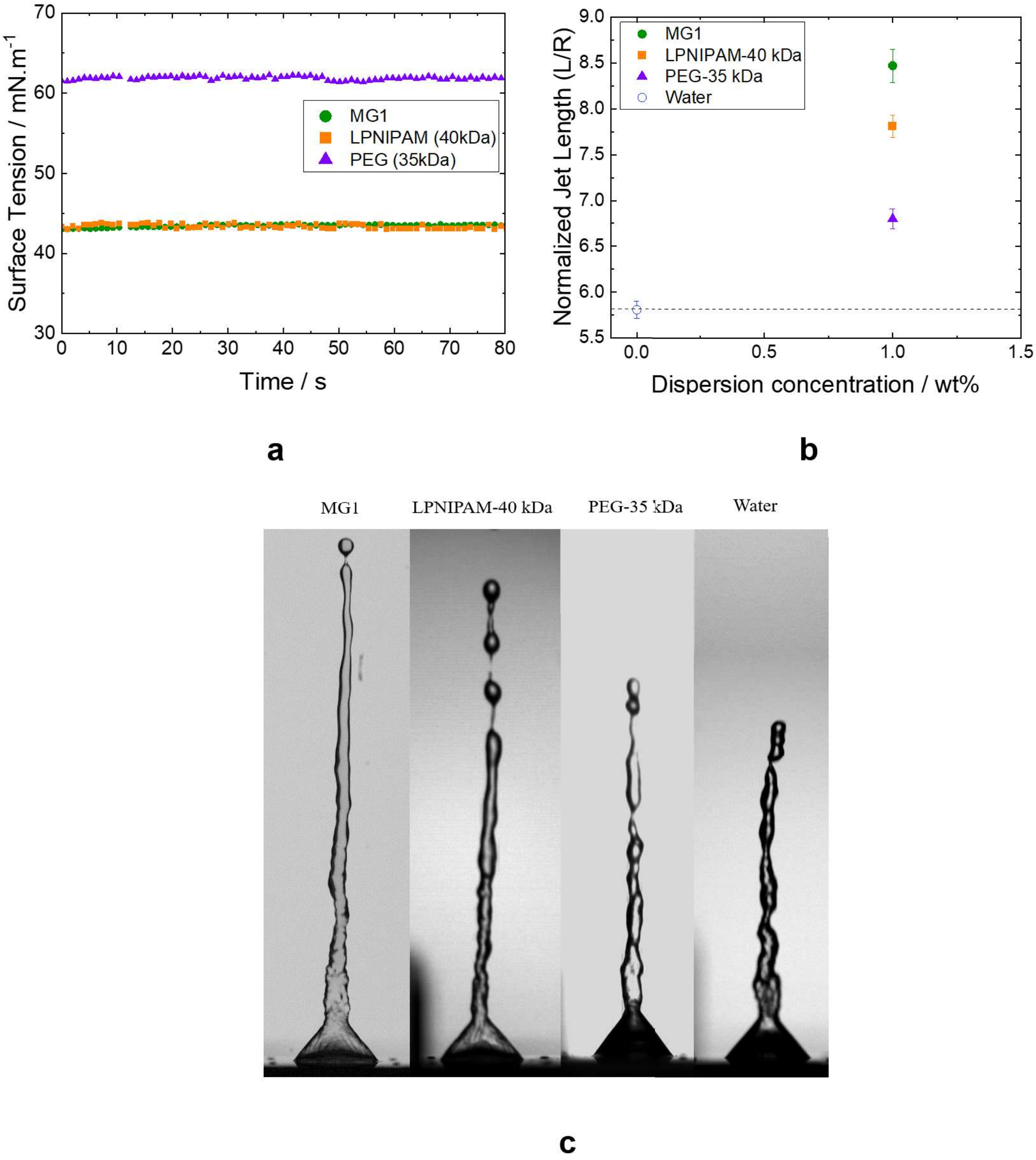

Figure R1: **Additional polymeric reference systems.** (a) Surface tension kinetics of softest microgel (MG1), Linear PNIPAM (M_w=40 kDa) as well as PEG (M_w=35 kDa) solutions at 1 wt% solution concentration. (b) The comparison of the normalized jet length across these systems. (c) The snapshot of the maximum jet length for MG1, linear PNIPAM and PEG solutions as well as water.

microgels. This additional structural contribution is absent in PEG and differs fundamentally from the dynamic replenishment mechanism available to LPNIPAM [see Fig. R2]. We recognize, however, that the present experiments cannot completely isolate network mechanics from all other microgel-specific interfacial properties. Accordingly, we have revised the manuscript to distinguish more clearly between the experimental observations and their mechanistic interpretation. Rather than attributing the stabilization solely to soft microgel network mechanics, we now conclude that jet stabilization reflects PNIPAM-associated interfacial effects together with an additional contribution associated with microgel-specific architecture.

Finally, we have clarified the broader message of the manuscript. The significance of the microgel system is not that it necessarily produces longer jets than every linear-polymer formulation. Rather, it provides a distinct route to jet control: the nanometer-scale softness and crosslinking of a pre-adsorbed microgel layer can be tuned to regulate centimeter-scale jet dynamics through interfacial mechanics. This interpretation is consistent with previous work showing that microgels can adsorb, spread, and form mechanically responsive layers at fluid interfaces [F. Camerin *et al.*, *Phys. Rev. X* 2020, 10, 031012]. In contrast, linear-polymer-mediated jet stabilization may also involve bulk extensional stresses, depending on polymer molecular weight and flow conditions [Anna and McKinley, *J. Rheol.* 2001, 45, 115–138, and Campo-Deaño and Clasen, *J. Non-Newtonian Fluid Mech.* 2010, 165, 1688–1699]. We therefore emphasize in the revised Abstract and Discussion that soft microgels offer a tunable, interfacially mediated design strategy for controlling nozzle-free jetting.

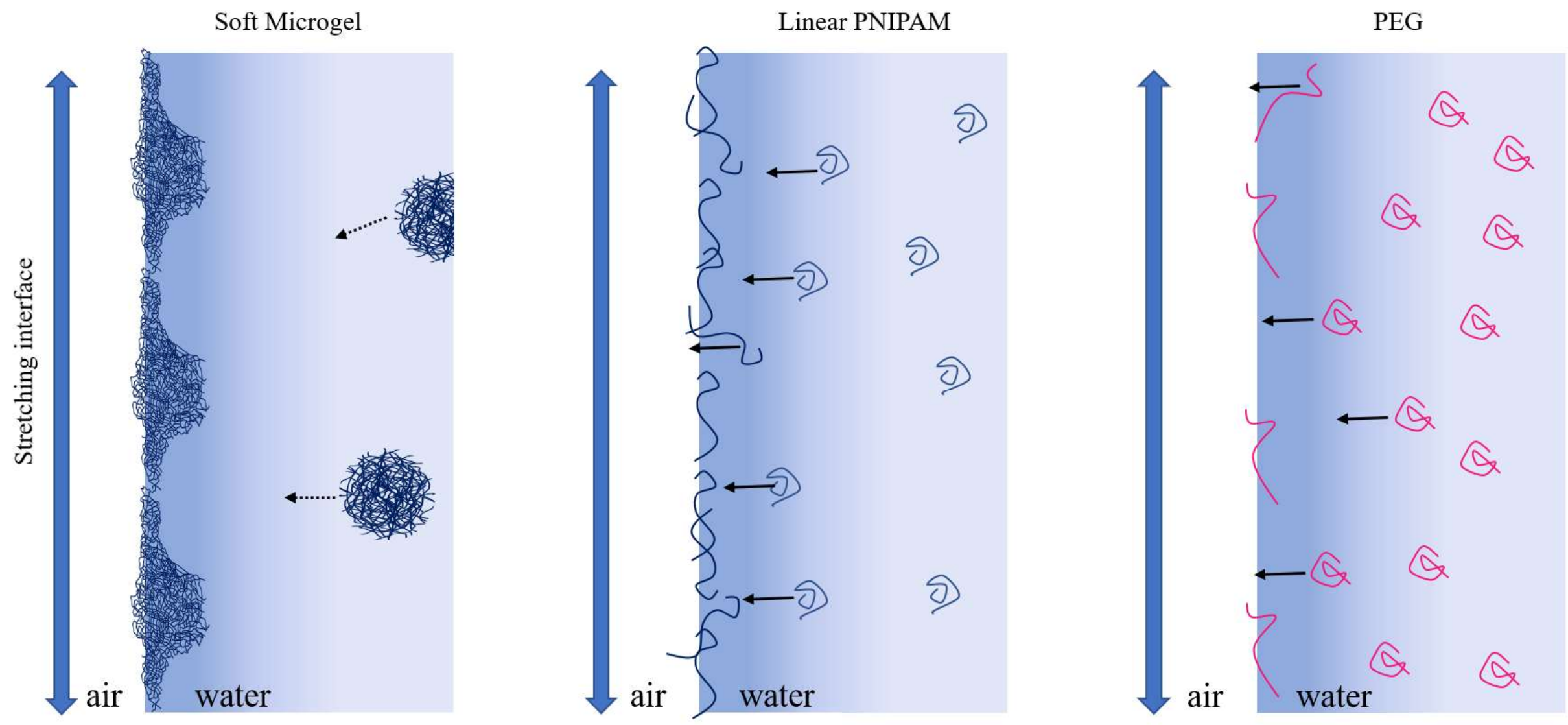

Figure R2: **Schematic illustration of the proposed stabilization mechanism.** The left panel shows a soft PNIPAM microgel adsorbed at the air–water interface. Owing to its deformability, the microgel spreads extensively at the interface, maintaining a low surface tension, while its large size results in relatively slow diffusion from the bulk (dotted arrows). The middle panel depicts linear PNIPAM chains, which diffuse rapidly from the bulk (solid arrows) and exhibit high surface activity, enabling efficient adsorption at the interface. In contrast, linear PEG chains (right panel) also diffuse rapidly but possess substantially lower surface activity, resulting in reduced adsorption and lower surface density than linear PNIPAM chains at the air–water interface.

We added these figures to the supporting information and revised the conclusion accordingly:

'*To separate the roles of polymer chemistry, interfacial activity, bulk polymer effects, and microgel architecture, we compared soft microgels with linear PNIPAM ($M_w = 40$ kDa) and PEG ($M_w = 35$ kDa) (Supplementary Fig. S13). PEG, despite its comparable molecular weight, exhibits a higher surface tension and produces much shorter jets than PNIPAM-based systems, showing that dissolved polymer chains alone are insufficient for stabilization. Instead, PNIPAM-mediated interfacial activity is essential. However, linear PNIPAM and microgels stabilize newly created interfaces differently. Microgels diffuse over their average interparticle spacing on second timescales, far slower than the millisecond jetting process, whereas 40 kDa PNIPAM chains can access newly created interface within microseconds (Supplementary Fig. S14). Thus, microgel stabilization is governed mainly by the pre-adsorbed interfacial layer, while linear PNIPAM can additionally adsorb during extension. Although the present experiments do not fully isolate network mechanics from other microgel-specific interfacial properties, our results indicate that the deformable microgel architecture contributes beyond PNIPAM-mediated surface activity. This is consistent with*

microgels forming mechanically responsive layers at fluid interfaces [57]. More broadly, soft microgels provide a distinct route to jet control, where nanometer-scale crosslink density and softness of a pre-adsorbed layer regulate centimeter-scale jet dynamics through interfacial mechanics, in contrast to high-molecular-weight linear polymers, where bulk extensional stresses may also contribute[52–56].'
